# Burden-aware feedback control of microbial consortia

**DOI:** 10.1038/s41467-026-72389-6

**Published:** 2026-05-06

**Authors:** Alice Boo, Harman Mehta, Rodrigo Ledesma-Amaro, Guy-Bart Stan

**Affiliations:** 1 Imperial College Centre for Excellence in Synthetic Biology, Imperial College London, London, SW7 2AZ UK; 2 Department of Bioengineering, Imperial College London, London, SW7 2AZ UK; 3 Bezos Centre for Sustainable Protein, Imperial College London, London, SW7 2AZ UK; 4 UKRI Engineering Biology Mission Hub on Microbial Food, Imperial College London, London, SW7 2AZ UK

**Keywords:** Synthetic biology, Synthetic biology, Synthetic biology

## Abstract

Engineered microbial consortia are emerging as programmable systems capable of sensing and responding to their environment. However, maintaining defined community composition over time remains challenging, particularly in bioprocesses where growth conditions and metabolic burdens continuously shift. Here, we develop a burden-aware multicellular RNA-based feedback control system that stabilises coculture composition by coupling gene expression burden to growth regulation. The system integrates three modules: quorum sensing-based communication, an RNA-based comparator computing deviations from a target ratio, and tuneable growth regulation via heterologous expression burden or CRISPRi-mediated knockdowns. In a two-strain *E. coli* coculture, this architecture maintains stable coculture ratios over 24-hour batch cultures, recovers growth rates by up to 90% following burden-induced growth reduction, and increases protein production yields by up to 81% in the slower-growing strain. We achieve tuneability by adjusting RNA binding strength and quorum-sensing signal production. This work demonstrates that burden-driven growth control can be used to stabilise and tune synthetic microbial consortia.

## Abstract (web summary)

Maintaining a defined community composition of engineered microbial consortia over time remains challenging. Here the authors develop a multicellular RNA-based feedback control system that stabiliszes coculture composition by coupling gene expression burden to growth regulation.

## Introduction

Over the past decade, there has been a growing interest in the interactions between microbes and their environment, leading to an explosion of publications on the microbiome. In 2020, over 20,000 articles were published on the microbiome, driven by the quest to uncover the causality between disease and microbiota for the development of microbiome-based therapeutics^1–6^. Beyond the human microbiome, microbial communities have gained attention for their potential in diverse sectors, including agriculture^7–11^, bioremediation^12–14^, food production and waste valorisation^15–19^. Synthetic biology has enabled the engineering of microbial communities, which can be created or manipulated to perform enhanced or new functions by exploiting the strengths and specificities of each microbe in the community. By dividing complex tasks into smaller ones, the energy expenditure of each microbe is minimised, allowing community microbes to grow better and achieve higher production yields of complex compounds than monocultures^20–28^. It also enables the bioproduction of high-value molecules from waste products such as plastic, methane or waste derived from the agriculture and food industries^29–31^. Additionally, compartmentalising genetic circuits and pathways across different microbial species increases the modularity and reusability of genetic parts and modules, thus opening new avenues for distributed biocomputing and multicellular control strategies^32–40^. These features of engineered microbial communities position them ideally to be explored for the development of sustainable innovations as the bioeconomy continues to grow.

However, establishing and maintaining stable microbial communities remains challenging because competitive interactions can drive one member to dominate, particularly in well-mixed environments and long-term cultures^41,42^. This challenge is magnified in fed-batch and continuous processes, where subtle differences in growth rate, nutrient access, or burden can progressively shift community structure over time^43,44^. To address this, a broad set of strategies has been developed to stabilise coculture composition. A major class relies on auxotrophic or syntrophic cross-feeding, where strains exchange essential metabolites to enforce mutual dependence and promote coexistence. Engineered auxotrophic yeast and bacterial communities have been shown to form stable mutualisms across nutrient regimes and to enhance pathway efficiency and product titres^45–52^. Ecology-guided community design and rational strain-selection strategies leveraging large-scale experiments or computational modelling have also been used to identify strain combinations that self-organise into desired compositions^50,53–56^. In parallel, manipulation of environmental conditions such as carbon-source switching, substrate pulsing, modulation of nutrient limitation, or temperature has been used to steer coculture composition by periodically shifting the fitness advantage between partners^44,57–60^. Beyond genetic or metabolic control, recent modelling shows that physical traits such as cell size or shape, when combined with spatial confinement, can create emergent competitive advantages and influence strain composition over time^61^.

In parallel, synthetic biology has enabled the engineering of microbial consortia through modular interaction circuits and rational community design. Quorum-sensing-based population control circuits link cell density to regulated growth or killing, enabling autonomous control of population size in monocultures and multi-strain communities^41,42,62–69^. Many implementations rely on toxin-mediated lysis, which has been particularly effective in therapeutic contexts, where synchronised lysis controls bacterial load and enables drug delivery in vivo^64,70–72^. Beyond lysis strategies, quorum sensing has also been used to modulate relative growth rates between subpopulations, enabling multiple stable community states through signal-dependent fitness advantages^73^. More broadly, quorum-sensing-regulated genetic circuits and modular design frameworks have enabled the construction of microbial consortia with programmable ecological relationships and predictable compositional outcomes^68,74^.

Cybergenetic and optogenetic control frameworks have introduced external, feedback-based strategies to achieve precise compositional control. In these systems, online measurements of strain abundance are used to compute control actions such as light intensity or nutrient input that modulate growth in real time, enabling robust tracking of user-defined strain ratios^65,75–79^. In contrast, alternative approaches based on programmed differentiation or recombinase-mediated branching can generate defined coculture ratios from a single founder population but typically lack continuous feedback to maintain composition under changing conditions^80,81^. Together, interaction engineering, environmental modulation, and genetic or cybergenetic control provide a diverse toolbox for stabilising and steering microbial consortia. Collectively, these approaches either fix community ratios through circuit design or initial conditions or rely on external monitoring and actuation to adjust coculture ratios over time. This creates an opportunity for genetic controllers that continuously sense population imbalance and adjust growth from within the community.

The concept of metabolic burden arising from heterologous gene expression was first recognised in metabolic engineering, where overexpression of recombinant pathways was observed to reduce host growth and productivity due to competition for cellular resources^82,83^. Subsequent work formalised this phenomenon in the context of cellular resource allocation, linking gene expression to growth through competition for transcriptional and translational machinery^84–86^. In engineered strains, heterologous expression diverts ribosomes and RNA polymerase away from native processes, creating metabolic burden that slows growth, alters host physiology, and places a hard limit on genetic circuit performance^86–90^. Resource competition models show that this coupling drives coordinated changes in growth rate and gene expression through ribosome allocation and cellular capacity^85,91–96^. In microbial consortia, unequal burden carried by different strains can progressively shift coculture ratios and destabilise community structure over time, particularly under high expression loads or in long-duration cultures. Burden-aware feedback controllers have successfully restored growth and increased productivity in monocultures by sensing this cost and adjusting expression accordingly^97^. These advances highlight burden as both a central factor influencing community stability and a promising internal signal that could be leveraged to autonomously regulate coculture composition.

**Fig. 1:**
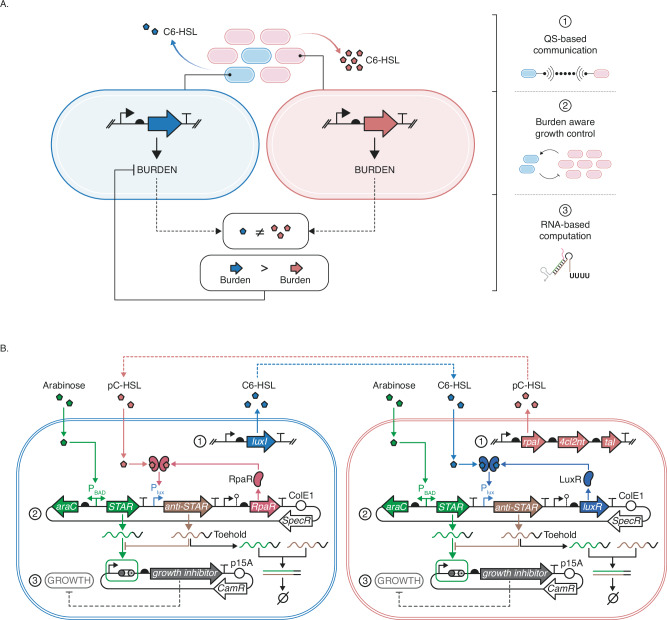
A hybrid quorum and RNA-based control system for stabilising population composition in a microbial coculture system. **A** Three modules were built using a bottom-up part assembly strategy to engineer a system controlling population composition in a two-strain *E. coli* consortium. The communication module propagates information about the population density of each of the two strains. The RNA-based comparator built from a set of STAR and anti-STAR parts is designed to compare bacterial population density to an inducible reference signal. The output signal of the comparator is used by the growth module to determine whether the cell density of each strain should be up- or down-regulated to maintain a stable coculture composition. **B** Proposed circuit for controlling community composition. Each bacterial population produces a specific quorum-sensing molecule (e.g., C6-HSL or pC-HSL) that reflects population density and can be detected by the other population. A synthetic RNA (anti-STAR) is produced upon detection of quorum molecules in each population and is compared to a reference signal (STAR). STAR and anti-STAR are designed to bind to each other and form an inactive complex. Free STAR can bind to the termination hairpin to allow transcription of the growth regulation gene. Anti-STAR acts as a STAR-sequestration buffer, preventing STAR from binding to the termination hairpin.

To address this need, we developed an RNA-based comparator that autonomously evaluates population imbalance and directs growth regulation within a coculture. Here, we define the RNA comparator as a molecular module that evaluates differences in population density through RNA sequestration (STAR/anti-STAR) and converts this information into a regulatory output. This module computes the density difference between two *E. coli* strains and adjusts their relative burden from heterologous gene expression to rebalance coculture ratios, which enables dynamic composition control without relying on cell death or growth arrest. Our strategy uses the inherent burden associated with the expression of the protein of interest as a direct mechanism for growth control. The design includes three modules: a communication module using quorum sensing to track the density of the cocultured strains, a comparator module to evaluate differences from a desired ratio and a growth module that tunes exogenous gene expression to modulate growth and achieve the target population composition (Fig. 1).

Our comparator module relies on small transcription activating RNA (STAR), a cis-acting RNA-based transcription regulation device that uses small RNAs to activate gene transcription^98–100^. STAR operates by targeting a sequence designed to form a strong termination hairpin, which ordinarily halts transcriptional elongation. Upon transcription, STAR binds to this hairpin, which prevents terminator formation and allows transcription of the downstream gene. By integrating this mechanism, we constructed a comparator that detects deviations in relative cell density and corrects them by dynamically adjusting the burden imposed on each strain. RNA-based regulatory systems offer a dynamic complement to protein-based regulators, as they enable predictable Watson-Crick base pairing for high orthogonality and can respond rapidly through direct transduction at the RNA level^101–103^. Such systems have been used to implement tuneable gene expression and feedback control in bacteria, including through transcriptional and translational regulation, antisense RNA interactions, and molecular sequestration mechanisms^69,99,104–110^. By leveraging the rapid action of RNA and using expression burden as a growth regulatory mechanism, the consortium can maintain a defined coculture composition while sustaining high productivity.

## Results

### A three-module architecture for tuning population composition in a microbial coculture

Inspired by protein-based sequestration mechanisms such as sigma and anti-sigma factors^111^, we designed a general architecture using RNA-RNA interactions to control population composition of a two-member *E. coli* coculture. Commonly, synthetic gene circuits are assembled using a bottom-up approach that consists of connecting characterised parts together to form modules, which, when combined, form genetic devices^112,113^. From a selected set of parts used to express quorum-sensing molecules, regulatory RNAs and growth regulators, we constructed three modules: a communication module, a comparison module and a growth module (Fig. 1A). The communication module relies on quorum-sensing molecules to estimate the density of bacterial populations. This information is used by the comparison module to estimate the differences between actual and desired ratios of the two cell types. Using this information, the growth module controls cell growth in both populations to minimise the ratiometric error. In Fig. 1B, we present the general architecture of the RNA-based sequestration mechanism that can be used to regulate coculture composition. C6-HSL and pC-HSL AI-1 quorum-sensing molecules were selected to convey information about population densities as they were previously reported to be signal orthogonal, i.e., they are orthogonal if their cognate regulator proteins LuxR and RpaR are physically compartmentalised^114–116^. Additionally, Scott et al. showed that the LLL quorum-sensing system, i.e., the system in which C6-HSL binds to the regulator LuxR and activates the lux promoter (pLux), exhibits similar properties to the LRR system, for which pC-HSL binds to the RpaR regulator to activate pLux^115^. C6-HSL production by C6-HSL sender strains requires the expression of a single enzyme, HSL synthase LuxI from *Vibrio fischeri*^117–119^, while pC-HSL production by pC-HSL sender strains requires the expression of three enzymes: tyrosine ammonia lyase (*TAL*) from *Saccharothrix espanaensis*, 4-coumarate-CoA ligase from *Nicotiana tabacum* (*4CL2nt*) and HSL synthase RpaI, from *Rhodopseudomonas palustris*^120^. The comparator relies on the sequestration of two RNA species: STAR and anti-STAR. STAR expression is controlled by an inducible promoter, which sets the desired ratio of the coculture. For example, to stabilise the coculture composition around a 1:1 ratio, we can control STAR expression in both strains by the same inducible promoter, e.g., the arabinose promoter (pBAD). Anti-STAR is controlled by the quorum-sensing signal representing the cell density of the opposite strain. Therefore, as anti-STAR sequesters STAR into a complex degraded by the cell’s native RNase E, less STAR is available to bind to the STAR target, thus reducing the expression of the downstream growth regulator or gene of interest. To minimise the toxicity of the multicellular feedback, we decided to either regulate the cell growth rate through an RNA-mediated essential gene knockdown or by modulating the burden of the gene(s) of interest, thus the circuit did not require an additional gene to regulate growth rate beyond the exogenous gene(s) required to perform the function of interest in the microbial consortium. To validate whether burden could be successfully used for regulating coculture compositions, we constructed a mechanistic mathematical model describing the three modules and their impact on the growth of two bacterial species sharing a single growth compartment (Supplementary Fig. 1, Supplementary Note 1). As opposed to previously described coculture control strategies involving the expression of a toxin that negatively impacts bacterial growth rates, burden slows down growth proportionally to the concentration of the burdensome protein expressed by the bacterial populations^41,66,121^. We found that our strategy successfully stabilised the coculture composition when different burdens were imposed on the two populations. In addition, the coculture ratio was tuneable by varying the expression of STAR and anti-STAR in the system.

### Designing an RNA-based molecular sequestration system to stabilise coculture composition

**Fig. 2:**
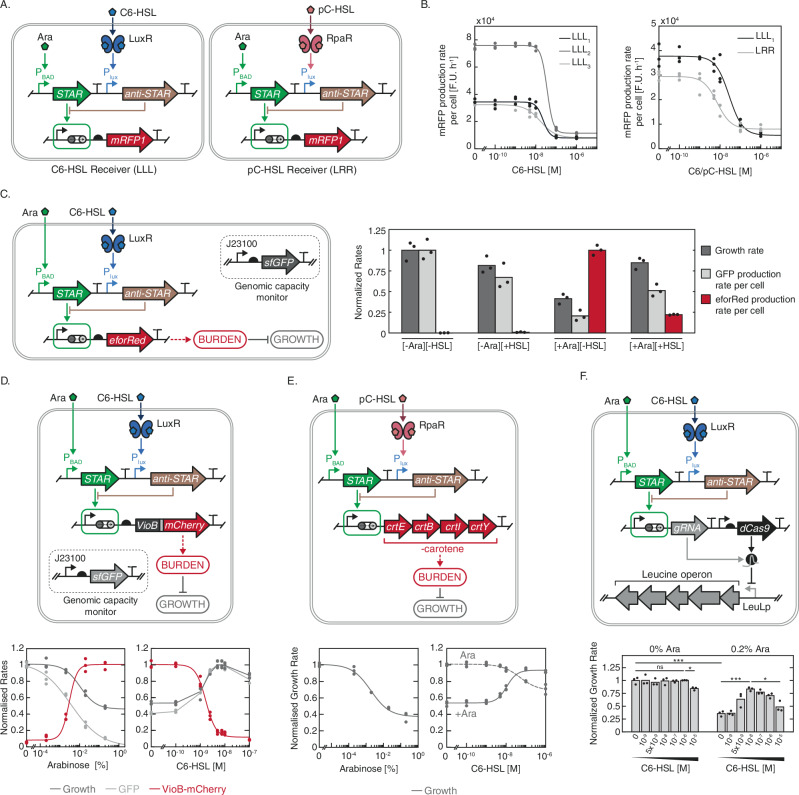
Building a STAR-based comparator for bacteria growth control. **A** Genetic circuits of the LLL and LRR STAR-based comparators. The LLL and LRR comparators compare a L-arabinose inducible reference signal to C6-HSL and pC-HSL signals, respectively. **B** mRFP production rate of the LLL_1_, LLL_2_, LLL_3_, and LRR comparators supplemented with 0 M to 10^−7^ M of C6-HSL or pC-HSL. **C** Normalised growth rate (dark grey), GFP production rate (light grey), and eforRed production rate (red) in the DH10B-GFP capacity monitor strain. Cultures were supplemented with 0% (−Ara) or 0.2% (+Ara) L-arabinose, and 0 M (−HSL) or 10^−7^ M (+HSL) C6-HSL. **D** The comparator controls growth by expressing VioB-mCherry in the DH10B-GFP capacity monitor strain. Normalised growth rate (dark grey), GFP production rate (light grey) and VioB-mCherry production rate (red) with increasing concentrations of L-arabinose and C6-HSL. Growth rate and GFP production were normalised against condition: 0 M C6-HSL and 0% L-arabinose. VioB-mCherry production rate was normalised against condition: 0 M C6-HSL and 0.2% L-arabinose. **E** The comparator controls growth by expressing β-carotene in DH10B. Normalised growth rate with increased concentrations of L-arabinose and pC-HSL. Growth rate was normalised against condition: 0 M of pC-HSL and 0% of L-arabinose. **F** The comparator controls growth by expressing a gRNA targeting the LeuLp genomic promoter in BW25113. Normalised growth rates with 0% or 0.2% L-arabinose and increasing concentrations of C6-HSL. Growth rate was normalised against condition: 0 M of C6-HSL and 0% of L-arabinose. For all panels, OD and fluorescence data were collected using a microplate reader. The data points represent 3 biological replicates, and the bars represent the means of these points. The curves were fitted to the means of these points using MATLAB’s four-parameter nonlinear regression fit; when possible, otherwise the means were used to plot the lines. Statistically significant differences were determined using a two-tailed Student’s *t*-test (*** represents *p* < 0.001, ** represents *p* < 0.01, * represents *p* < 0.1, ns represents not significant). Plasmids used in this figure are recorded in Supplementary Data 2 and strains in Supplementary Table 1. Source data are provided as a Source Data file.

The RNA-based comparator forms the core of the multicellular feedback loop that regulates coculture composition in our two-strain consortia. Each strain should be equipped with an analogous, yet orthogonal version of the comparator to avoid crosstalk when evaluating the density of either population. For this, two versions of the comparator were implemented, one producing anti-STAR in response to C6-HSL (LLL comparator) and the other in response to pC-HSL (LRR comparator) (Fig. 2A). To test the comparator, STAR was placed under the control of the strong arabinose-inducible araBAD promoter and controlled the activation of mRFP1 expression. We built a small library of STARs and anti-STARs with six different toehold sequences from Green et al.^107^ (Supplementary Fig. 2). We also tested comparator variants with varying RBS strength driving LuxR. Hereafter, the LLL_1_ comparator expresses STAR and anti-STAR with toehold 2 (pAB300, Supplementary Data 2), LLL_2_ expresses STAR and anti-STAR without a toehold (pAB317), and LLL_3_ is a variant of LLL_1_ with *luxR* expressed with a stronger RBS (pAB545) (Supplementary Data 2). We observed that changing the hybridisation energy between STAR and anti-STAR changed the ON/OFF properties of the comparator as demonstrated by the LLL_1_ and LLL_2_ comparators results presented in Fig. 2B. When only expressing STAR from the LLL_1_ and LLL_2_ designs, mRFP production rate was 2-fold higher for LLL_2_ than for LLL_1_ (Fig. 2B, Supplementary Fig. 3). The properties of the comparator can also be tuned by increasing the expression of the LuxR regulator, which changes the slope of the input-output response curve as shown by results from the LLL_1_ and LLL_3_ comparators, which share the same toehold sequence (Fig. 2B). Introducing a tandem STAR termination hairpin decreased both the OFF and ON states, limiting the dynamic range of the comparator (Supplementary Fig. 4). As the ON state of this design was low, we did not use it further in this work, but we hypothesised that this design could be suitable for specific applications such as for expressing highly toxic products which require very tight and low gene expression. The LRR comparator, which uses the same STAR and anti-STAR design as the LLL_1_ comparator, has a similar operating range as that of LLL_1_ (Fig. 2C, Supplementary Fig. 5). However, the deactivation of STAR by anti-STAR was more efficient for the LLL_1_ comparator than for the LRR comparator, with a deactivation percentage of 93% for LLL_1_ compared to 78% for LRR (Supplementary Fig. 6). It is therefore possible to tune the output of the comparator by playing with the comparator’s RNA–RNA hybridisation energy properties as well as by tuning the level of anti-STAR expression by using weaker or stronger HSL producers.

### Controlling growth rates with the output of the RNA-based comparator

Having demonstrated that the RNA-based comparator can be used to modulate the level of expression in response to the concentration difference of two signal-orthogonal quorum-sensing molecules, we investigated how to control bacterial growth rate while using a minimal amount of host resources. For this, we showed that the comparator can be coupled to the expression of a gene of interest, which in turn impacts cell growth rate. We investigated four types of growth regulators: (1) a small heterologous protein: eforRed, (2) a large heterologous protein: VioB, (3) a metabolic pathway to express β-carotene, and (4) CRISPRi targeting *E. coli*’s native leucine operon.

First, we used the capacity monitor strain, an *E. coli* strain genomically integrated with a constitutively expressed GFP previously developed by Ceroni et al.^90^, to monitor the burden caused by expressing a small heterologous gene of interest, eforRed. As expression of eforRed increases, cellular resources are pulled away from other processes, thus decreasing GFP expression from the capacity monitor. We observed that inducing eforRed expression reduced GFP capacity by 80% and growth rate by 60%. GFP capacity and growth rate were calculated from fluorescence and OD measurements as described in the Methods. In addition, when coupling eforRed expression to our comparator, in the presence of C6-HSL, anti-STAR is maximally expressed and GFP capacity is recovered up to 60% of its original value, while growth rate is recovered up to 90% of the original value observed when eforRed is not expressed (Fig. 2C). We extended this analysis to three additional chromoproteins (gfasPurple, cjBlue and fwYellow), each imposing varying degrees of growth inhibition (Supplementary Fig. 7). When coupled to the LLL_1_ controller, all three recovered up to 90% or more of the growth rate observed in the absence of chromoprotein expression, without external addition of L-arabinose. We note that GFP capacity is not fully recovered when anti-STAR is expressed, and that GFP capacity is impacted by anti-STAR expression (Fig. 2C). This reflects the cost of expressing the controller species, STAR and anti-STAR (Supplementary Fig. 8). To further explore if burden could be used to regulate growth rate, we expressed VioB-mCherry, a large fusion protein previously shown to impose burden on *E. coli* (Fig. 2D). Cellular growth rate was reduced by 53% when expressing VioB-mCherry. Sequestration by anti-STAR led to restoring the host growth rate up to 90% of the original value measured when STAR is not expressed, i.e., in the absence of the STAR inducer, L-arabinose (Fig. 2D, Supplementary Fig. 9). Effective growth regulation required sufficiently high expression of VioB-mCherry to impose a measurable burden on the cell (Supplementary Fig. 9)^90,97^. The comparator could also tune burden, and by extension growth rate, caused by the expression of metabolic pathways such as the β-carotene pathway (Fig. 2E and Supplementary Fig. 10)^122^. Expressing the β-carotene pathway resulted in a maximum decrease in growth rate of about 50 to 60% when inducing the circuit with 0.2% L-arabinose. Expressing anti-STAR by inducing the system with 1 µM pC-HSL was able to restore the growth rate up to 90% of the original value measured when no STAR was expressed (0% L-arabinose). Finally, we linked the output of the comparator to a CRISPRi system targeting the leucine operon to build a tuneable amino acid knockdown (Fig. 2F). We designed a gRNA targeting the native LeuLp promoter driving *E. coli* BW25113’s leucine operon (Supplementary Fig. 11) and repress cellular growth (Supplementary Fig. 12). A similar strategy targeting the HisLp promoter controlling the histidine operon of *E. coli* BW25113 also repressed growth (Supplementary Fig. 12). However, repression of the leucine operon with a fully complementary guide sequence to the LeuLp promoter gave rise to extended lag-phases of over 20 h but no reduction in growth rate (Supplementary Fig. 13). As an extended lag phase is not a desirable property to build our multicellular feedback controller, we introduced a single base-pair mutation in the guide sequence^97^ to tune CRISPRi inhibition level of the leucine pathway and found that a mismatch preceding the PAM sequence could alleviate CRISPRi repression and shorten the lag-phase (Supplementary Fig. 14). By regulating the expression level of the gRNA, the comparator could reduce cellular growth rate by 64%, and STAR sequestration by anti-STAR was able to recover growth rate up to 80% of its original value when no gRNA was expressed, i.e., in the absence of L-arabinose.

Taken together, these approaches demonstrate that the STAR and anti-STAR sequestration system can successfully up- and down-regulate growth rate following a quorum-sensing input. We envision that the controller could be tuned further via the addition of external molecules, such as L-leucine, which inhibits *E. coli* K-12 strains’ growth in the absence of L-isoleucine (Supplementary Fig. 15). This would give an additional level of control to temporally adjust the population composition regulated autonomously by the STAR-based controller.

### Stabilising the ratio of an unbalanced *E. coli* coculture using gene expression burden

To realise the multicellular nature of the circuit, it was then necessary to build a set of quorum sender/receiver strains that would enable each comparator to respond to population density by adjusting growth rates at the population level. To this aim, we engineered a set of sender strains that produce either C6-HSL or pC-HSL by expressing *luxI* or the 3-gene *rpa* operon, respectively, under varying promoter and RBS strengths. These genes were genomically integrated to minimise the impact of their expression on the growth of the *E. coli* DH10B strain (Supplementary Fig. 16). We designed an assay to characterise the transcription activation profile of the quorum-sensing lux promoter in response to varying concentrations of homoserine lactone (HSL) molecules produced by the sender strains. To do so, we used dBroccoli, a fluorescent RNA aptamer that was previously used to characterise mRNA expression in *E. coli* cells and that does not impact cell growth upon induction (Supplementary Fig. 17)^123^. To quantify C6-HSL when expressing the LuxR regulator (or pC-HSL when expressing RpaR), we created receiver plasmids where dBroccoli is placed downstream of the lux promoter (Supplementary Fig. 18). To test whether the receiver strains could detect the concentrations of HSL produced by the sender strains, we grew the sender strains separately for 1–6 h and collected their supernatants by centrifugation. We then mixed the supernatants with the LLL and LRR receiver strains and measured the fluorescence emitted by dBroccoli. All receiver strains could detect a significant difference in HSL concentration between the supernatants collected at 1 and 6 h for all strains, except for the Lux5 and Rpa6 strains which already produced saturating amounts of HSL after 1 h of growth (Supplementary Fig. 19). Temporal responses of the receiver strains to the quorum sensing produced by the Lux1 and Rpa5 strains at every hour are shown in Supplementary Fig. 20. The strain libraries would therefore serve as a basis to tune the strength of the communication signals between the strains grown as a coculture (Supplementary Fig. 21).

**Fig. 3:**
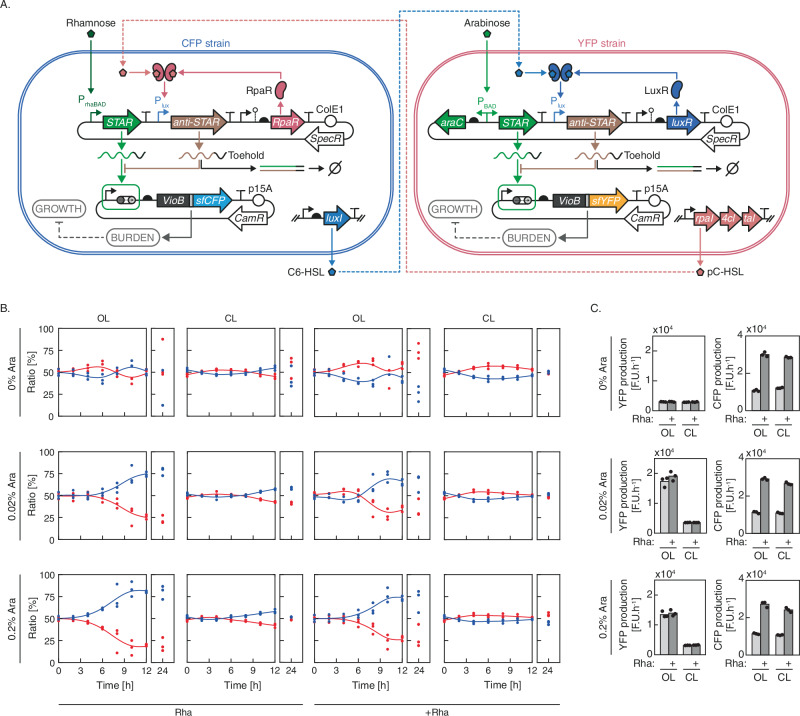
Stabilisation of coculture composition using a burden-driven growth control strategy. **A** Genetic circuit of the closed-loop two-member *E. coli* coculture. The CFP strain produces C6-HSL that inhibits VioB-YFP expression in the YFP strain, while the YFP strain produces pC-HSL that inhibits VioB-CFP expression in the CFP strain. Growth rate is controlled by the STAR-based comparator by tuning gene expression burden caused by VioB-YFP and VioB-CFP expression. **B** Population composition of the open-loop (OL) and closed-loop (CL) cocultures when induced with combinations of L-rhamnose (0%, 0.2%) and L-arabinose (0%, 0.02%, 0.2%). The OL design was composed of DH10B carrying the pAB537 and pAB519 plasmids and DH10B-mScarlet carrying the pAB317 and pAB518 plasmids, and the CL design was composed of the *E. coli* Lux5 strain carrying plasmids pAB537 and pAB519 and the *E. coli* Rpa5 carrying plasmids pAB317 and pAB518. In the presence of L-arabinose, the CL coculture corresponds to the circuit depicted in panel (**A**). The OL coculture corresponds to the circuit in panel A in which neither the CFP strain nor the YFP strain produces quorum-sensing molecules. **C** VioB-YFP and VioB-CFP production rates of the cocultures from panel B. “−Rha” and “+Rha” correspond to 0% and 0.2% L-rhamnose, respectively. Coculture compositions were determined by flow cytometry. OD and fluorescence data were collected using a microplate reader. The data points represent three biological replicates, and the bars represent the means of these points. The curves were fitted to the means of these points using a smoothing spline. Plasmids used in this figure are recorded in Supplementary Data 2 and strains in Supplementary Table 1. Source data are provided as a Source Data file.

Next, we connected the three modules (communication, comparison and growth regulation) to demonstrate how an RNA-based sequestration controller can stabilise the coculture composition of an unbalanced coculture system around a desired ratio. For this, we used a coculture expressing VioB-CFP and VioB-YFP under the control of comparators expressing STAR from the rhaBAD and the araBAD promoters, respectively (Fig. 3A). To assess the performance of our multicellular feedback, we built two versions of the controller: a closed-loop and an open-loop version. In the closed-loop coculture (CL), the CFP strain produces C6-HSL while the YFP strain produces pC-HSL. The open-loop coculture (OL) carries the same circuit as that of the CL, neither strain produces quorum-sensing molecules, and, as a result, the controller does not receive information about the strains’ densities.

In Fig. 3B, we showed that for the OL cocultures, as we externally supply neither, either or both L-rhamnose and L-arabinose to the coculture, expression of VioB in either or both strains drives the coculture composition out of equilibrium. However, in the CL system, exchange of quorum-sensing molecules leads to the stabilisation of the coculture composition around a 1:1 ratio. This is achieved through downregulation of the expression of VioB in the slowest growing strain, which in this case is the YFP strain. In the CL, VioB-sfCFP is not downregulated, while VioB-sfYFP is always downregulated (Fig. 3B). The YFP strain growth rate is more affected by the expression of VioB than the CFP strain for two reasons. First, the araBAD promoter is stronger than the rhaBAD promoter (Supplementary Fig. 22) and second, VioB-sfYFP expression is more burdensome than VioB-sfCFP expression (Supplementary Fig. 23). To gain more insight on the response of the CFP and YFP strains to the C6-HSL and pC-HSL sender cells, we collected the supernatants of the Lux5 and Rpa5 strains every hour for 6 hours, then measured the decrease in expression of VioB-sfYFP and VioB-sfCFP respectively and correlated growth rate recovery (Supplementary Fig. 24). The C6-HSL sender strain Lux5 and the pC-HSL sender strain Rpa5 were chosen as hosts for the CL circuit as weaker production of quorum-sensing molecules did not lead to the stabilisation of the coculture composition around a 1:1 ratio (Supplementary Figs. 25 and 26). We note, however, that tuning the expression of the quorum-sensing molecules is a way to stabilise the coculture composition around a wider range of ratios beyond the 1:1 ratio demonstrated in Fig. 3. These results demonstrate that our CL circuit can stabilise coculture composition when the expression of two heterologous genes causes different levels of burden, thus differentially affecting the growth of the cocultured strains.

### Demonstrating community composition tuneability and protein yield improvement

**Fig. 4:**
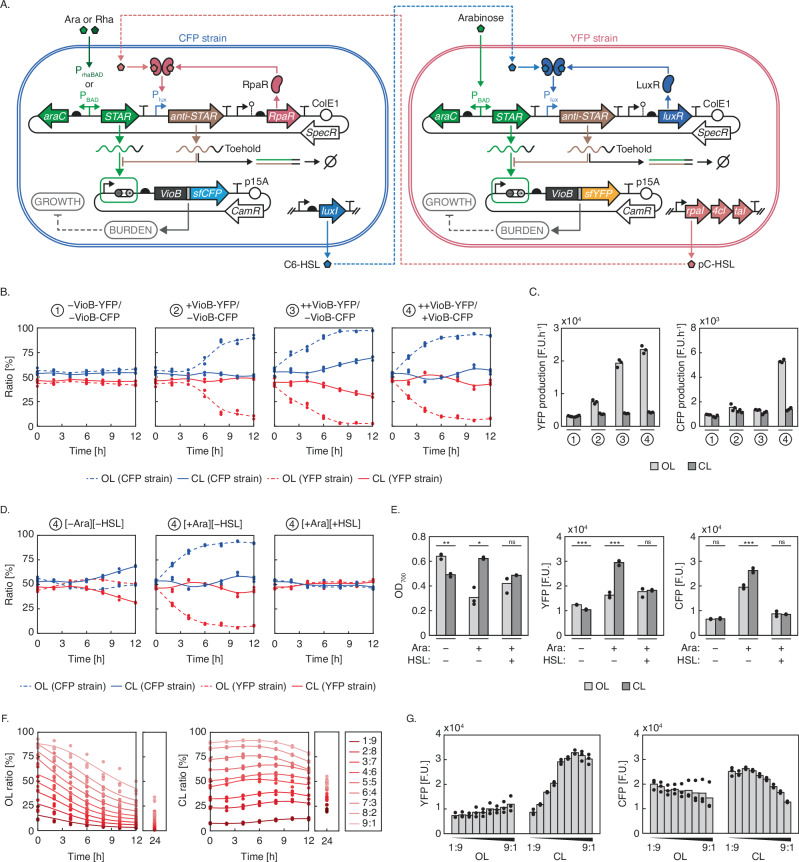
Demonstrating coculture composition tuneability. **A** Genetic circuit used to demonstrate coculture composition tuneability. **B** Effect of varying STAR expression in the CFP strain (DH10B-mScarlet for OL or Lux5 for CL) and YFP strain (DH10B-mScarlet for OL or Rpa5 for CL) on coculture composition. (1) CFP strain (carrying plasmids pAB519 and pAB537) and YFP strains (carrying plasmids pAB518 and pAB300) induced with 0% L-arabinose and 0% L-rhamnose. (2) CFP strain (carrying plasmids pAB519 and pAB537) and YFP strains (carrying plasmids pAB518 and pAB300) induced with 0% L-rhamnose and 0.2% L-arabinose. (3) CFP strains (carrying plasmids pAB519 and pAB537) and YFP strain (carrying plasmids pAB518 and pAB317) induced with 0% L-rhamnose and 0.2% L-arabinose. (4) CFP strain (carrying plasmids pAB519 and pAB401) and the YFP strain (carrying plasmids pAB518 and pAB317) induced with 0.2% L-rhamnose and 0.2% L-arabinose. “−”, “+”, “++” denote no, moderate and strong expression of VioB, respectively. **C** VioB-YFP and VioB-CFP production rates of panel **B** cocultures. **D** Coculture from panel B4 induced with: (1) 0% L-arabinose (−Ara), 0 M C6/pC-HSL (−HSL); (2) 0.2% L-arabinose (+Ara), 0 M C6/pC-HSL (−HSL); (3) 0.2% L-arabinose (+Ara) and 10^−7^ M C6/pC-HSL (+HSL). **E** Final OD, VioB-YFP and VioB-CFP fluorescence of panel **D** cocultures at 24 h. **F** OL and CL YFP strain fraction panel B4 coculture for different initial inoculation ratios. Both the OL and CL are induced with 0.2% L-rhamnose and 0.2% L-arabinose. **G** Final VioB-YFP and VioB-CFP fluorescence of panel **F** cocultures at 24 h. For all panels, coculture composition was determined by flow cytometry. OD and fluorescence data were collected using a microplate reader. The data points represent three biological replicates, and the bars represent the means of these points. The curves were fitted to the means of these points using a smoothing spline (OL, dashed; CL, solid; CFP, blue; YFP, red). Statistically significant differences were determined using a two-tailed Student’s *t*-test (*** represents *p* < 0.001, ** represents *p* < 0.01, * represents *p* < 0.1, ns represents not significant). Plasmids used in this figure are recorded in Supplementary Data 2 and strains in Supplementary Table 1. Source data are provided as a Source Data file.

Finally, we explored key parameters of the sequestration-based controller that could be tuned to modulate the composition of our two-strain cocultures (Fig. 4). Key parameters of the STAR-based comparator include the production rates of both STAR and anti-STAR, as well as tuning the sequestration dynamics using different toehold domains. As previously mentioned, we explored the impact of HSL production by one of the sender strains on coculture behaviour. We selected the pC-HSL-producing strains Rpa2, Rpa4 and Rpa5 from Supplementary Figs. 19 and 21 to tune sender production of pC-HSL, thus modulating the gain of anti-STAR production in the pC-HSL receiver strain (Supplementary Fig. 25). The Rpa2 strain is designated as a weak pC-HSL sender, Rpa4 as a medium-strength pC-HSL sender and Rpa5 as a strong pC-HSL sender. As predicted, increasing the production of pC-HSL by the CFP sender strain progressively decreased VioB-sfYFP expression (Supplementary Fig. 26). As a result, coculture composition was brought closer to a 1:1 ratio as pC-HSL production increased, upregulating anti-STAR production in the YFP strains and thus sequestering more STAR to prevent the production of VioB-sfYFP that destabilises the coculture composition. Another parameter that is interesting to tune is the maximal output of the STAR-based comparator by using different toehold domains. To this end, we used the LLL_1_ and LLL_2_ comparator designs from Fig. 2B to increase the output of the STAR comparator. As the output of the LLL_2_ comparator is 2-fold higher than that of LLL_1_, we used LLL_1_ for medium-strength STAR expression and LLL_2_ for high STAR expression. Doing so, we observed that as STAR expression increases, the YFP strain is more rapidly outcompeted by the CFP strain as VioB-sfYFP expression increases (Fig. 4B, C). If anti-STAR is not present in high enough concentrations, STAR is not fully sequestered, leading to stabilisation of the coculture at a 2:1 ratio. The composition stabilises around a 1:1 ratio, which is the same as the initial seeding ratio. The results confirm that tuning the input-output properties of the RNA-based comparator by changing the binding affinity of STAR and anti-STAR or by tuning anti-STAR expression, we can modify the composition of a two-member *E. coli* coculture.

To assess whether the controller could simultaneously balance burden and improve productivity, we examined its effect on biomass accumulation and protein yield. Looking at case 4 of Fig. 4B, for which both VioB-sfYFP and VioB-sfCFP are being expressed, we showed that when no L-arabinose is added, the CFP strain does not outcompete the YFP strain as no protein of interest is being produced (Fig. 4D, E). Interestingly, the CL coculture diverges from its equilibrium composition after 6 hours, which can be explained by the difference in burden caused by the different anti-STAR designs from the CFP and YFP strains (Supplementary Fig. 8). Next, in the presence of L-arabinose only, we observed that the OL coculture ratio is driven out of its equilibrium as the CFP strain quickly outcompetes the YFP strain. The CL coculture, however, can remain around a 1:1 ratio, keeping a stable coculture composition over time. When the system is induced with both L-arabinose and HSLs, the OL and CL cocultures both stabilise around a 1:1 ratio, demonstrating that the comparator can compensate for the difference in density of the two strains when quorum-sensing molecules are present in sufficiently large amounts. When looking at the final density of the cocultures, we observe that when externally inducing the system with L-arabinose, the CL coculture achieves a final density that is twofold higher than that of the OL coculture and reaches a similar density (0.6 ABS_700_) to the non-induced OL (Fig. 4E). This increase of biomass accumulation for the CL coculture translates to an 81% increase in total VioB-sfYFP produced and a 35% increase in total VioB-sfCFP produced after 24 hours compared to the performance of the OL coculture (Fig. 4E). By repressing VioB-sfYFP and VioB-sfCFP expression to balance the coculture composition, the controller effectively allowed both strains to grow better, thus improving biomass accumulation, which in turn led to higher production yields of the protein of interest. When both L-arabinose and HSLs were externally added into the medium, VioB-sfCFP and VioB-sfYFP expression were inhibited, resulting in little VioB-sfCFP and VioB-sfYFP being produced. Finally, we tested whether the STAR-based controller could stabilise population composition when the initial starting ratio was different from 1:1 (Fig. 4F). For this, we inoculated our OL and CL cocultures over a range of seeding ratios (1:9 to 9:1). After 12 h, the YFP strain fraction in all OL cocultures had dropped by at least 25% and as much as 55%. For the CL coculture, however, the YFP strain fractions appear to stabilise around their initial seeding ratio, not deviating from it more than 12%. Importantly, the CL coculture consistently reached higher final cell densities across all initial ratios except for 1:9, with an average increase of 50-60% over the OL condition (Supplementary Fig. 27). These results show that the closed-loop controller not only stabilises population composition but also enhances overall biomass accumulation, even when starting from highly skewed inoculation ratios.

## Discussion

Here, we present a multicellular RNA-based feedback control system capable of autonomously stabilising microbial coculture compositions. *E. coli* DH10B strains were engineered to express three low-burden genetic modules for bottom-up assembly: (a) a quorum-sensing-based communication module to sense coculture densities, (b) an RNA-based comparator module that evaluates and responds to strain coculture ratios, and (c) a growth regulation module that modulates cellular growth through gene expression burden. This integrated architecture dynamically corrects deviations from desired strain ratios using RNA sequestration responsive to quorum-sensing signals, enabling tuneable population-level control.

Our RNA-based comparator can modulate growth rates via several mechanisms, including burden regulation through expression of a single protein (chromoproteins), a biosynthetic pathway (beta-carotene), or essential gene knockdown using CRISPRi (leucine, histidine operons). The comparator RNAs themselves can also cause measurable growth burden, which means growth can be tuned through RNA expression alone. This feature allows the system to use the burden of expressing its own components as both the input and output for growth regulation, creating a feedback loop with minimal added complexity.

We demonstrated that the circuit, when split across two microbial strains, can stabilise coculture composition despite differences in growth rate caused by differential burden. The controller improved total product yield by 81% in the slowest-growing strain and by 35% in the faster-growing strain. Compared to strategies relying on metabolic cross-feeding, spatial organisation, or optogenetics, our RNA-based controller avoids irreversible biomass loss or complex hardware requirements. Instead, it uses burden, a ubiquitous feature of heterologous expression, as a generalisable and tuneable signal for feedback control.

Multiple circuit parameters offer opportunities for tuning coculture composition: quorum-sensing molecule production rates, transcription rates of genes of interest, promoter strengths, and binding affinities between STAR and anti-STAR species. In our current implementation, the STAR comparator is controlled by inducible promoters such as arabinose- or rhamnose-inducible systems. This choice allowed us to monitor dynamic responses during circuit testing and tune the system across a range of conditions. Moving forward, context-responsive promoters could replace chemical inducers to better suit fed-batch or continuous cultures^97,124–132^. These promoters could be designed to respond to stress, nutrient limitation, or product accumulation, enabling endogenous adjustment of growth. Hierarchical regulatory circuits could integrate these internal and external signals for more precise, context-aware control.

While the system is modular and tuneable, assessing its reliability under fluctuating environments will be important. Because the comparator operates through burden and RNA sequestration, performance may vary with factors that influence resource availability or RNA stability, including temperature, media composition or host-specific RNA turnover rates. The comparator also relies on differential quorum-sensing signal production and detection, so small variations in signal accumulation or binding affinity may affect ratio-sensing accuracy. Long-term stability and resistance to mutation remain key considerations for deployment in fed batch or continuous bioprocesses, and circuit chromosomal integration will require ensuring sufficient RNA expression and sequestration capacity at lower copy numbers^133^. Incorporating additional sensing modalities, such as metabolite or stress-responsive inputs, may further enhance robustness across hosts and process conditions, enabling the controller to maintain precise ratio control over extended time scales.

Our mathematical model helped capture the essential dynamics of burden-dependent growth and informed parameter selection. While simplified, the model provides a foundation for translation to continuous cultures or more complex environments. Future modelling efforts could incorporate dynamic RNA stability, resource allocation, or metabolite interactions to support predictive tuning. For example, in continuous cultures, higher levels of quorum-sensing signal production would likely be required to compensate for dilution. In parallel, theoretical studies have proposed that distributed feedback architectures could provide robust ratiometric control even when strains compete for shared resources^73,134–136^. The success of our burden-aware controller provides experimental support for these principles, and it suggests that expanding to additional feedback layers or additional species may further improve stability in fluctuating environments^137^.

The use of AI-1 derivative quorum-sensing molecules provided effective feedback, but their limited orthogonality may constrain expansion to additional strains or non-model organisms. Peptide-based autoinducers, such as those produced by Bacillus species, offer a promising alternative for broadening communication capabilities. Testing their compatibility with new hosts, including yeast, could further expand the range of applications. Yeast is a valuable chassis for metabolic engineering due to its ability to produce complex molecules such as terpenoids, alkaloids, and polyketides. Its slower RNA degradation could support more stable RNA-based circuits, but differences in splicing, capping, polyadenylation, and translation initiation mean bacterial RNA regulators are unlikely to function without redesign. RNA export from the nucleus and distinct decay pathways may also affect circuit performance.

Burden-aware, multicellular feedback control could be extended to non-model chassis such as *E. coli* BL21, *Bacillus subtilis*, or *Pseudomonas putida*, demonstrating its utility for industrial bioproduction. For instance, Zhu et al. (2022) engineered a coculture in which *E. coli* converted xylose into acetate and free fatty acids, while *P. putida* consumed these intermediates to synthesise medium-chain-length polyhydroxyalkanoates (mcl-PHAs)^138^. Precise timing and seeding ratios were required to maintain cooperation and obtain the best yields of mcI-PHAs. A dynamic RNA-based controller could replace this static tuning by autonomously adjusting strain growth based on coculture ratios, improving stability and yield. Similar principles could be applied to other functional consortia to distribute biosynthesis across chassis optimised for distinct tasks.

As synthetic biology increasingly relies on multi-strain and multi-species systems, burden-aware feedback circuits offer a practical solution to managing growth imbalances that limit productivity and stability. When combined with pathway engineering and quantitative monitoring, these systems could support the deployment of engineered consortia in contexts where load balancing is essential, such as distributed bioproduction or metabolism of complex feedstocks.

## Methods

### Bacterial strains and plasmids

All cloning was done in DH10B (K-12 F-λ-araD139 ∆(araA-leu)7697 ∆(lac)X74 galE15 galK16 galU hsdR2 relA rpsL150(StrR) spoT1 deoR ϕ80dlacZ∆M15 endA1 nupG recA1 e14-mcrA ∆(mrr hsdRMS mcrBC)) obtained from the National BioResource Project Japan. BW25113 (K-12 acI+rrnBT14 ∆lacZWJ16 hsdR514 ∆araBADAH33 ∆rhaBADLD78 rph-1 ∆(araB–D)567 ∆(rhaD–B)568 ∆lacZ4787(::rrnB-3) hsdR514 rph-1) and JW5807 (BW25113 ∆leuB) were obtained from the Keio Collection. pC-HSL and C6-HSL producing strains were built by integrating *luxI* and the rpa operon (*TAL, 4CL2nt, rpaI*) into the λ phage attachment locus of DH10B by CRIM integration^139^. We also inserted *mScarlet-I* and *sfGFP* as fluorescence markers for tracking coculture ratios. Genes were cloned into the CRIM integration vector plasmids pAH63 and propagated in *pir-116* electrocompetent *E. coli* cells (Lucigen). Integration, curation and validation of the integrated strains were done following the protocol from Haldimann et al.^139^. The bacterial strains used in this work are detailed in Supplementary Table 1. Gibson assembly (NEBuilder HiFi DNA Assembly Master Mix, NEB) and Golden Gate assembly based on the START/STOP toolkit were used to assemble plasmids^140^. All plasmid sequences were verified using Sanger sequencing. VioB and CRISPRi plasmids were adapted from Ceroni et al.^97^. STAR target plasmids were derived from plasmid pJBL5939^99^. STAR and anti-STAR sequences were synthesised by Integrated DNA Technologies. Genetic parts used to construct the plasmids generated in this study are available in Supplementary Data 1. The plasmids created in this study are detailed in Supplementary Data 2. Plasmid maps of the plasmids (Genbank) generated in this study are publicly available on Zenodo (10.5281/zenodo.18666365).

### Time-course fluorescence assays

Time-course experiments were performed in clear flat-bottom 96-well plates (Costar) with three biological replicates using a Tecan Spark microplate reader. Cells transformed with the constructs of interest and control plasmids were inoculated in 5 mL of M9 (M9 salts, 0.25 mg/mL thiamine hydrochloride, 2 mM MgSO_4_, 0.1 mM CaCl_2_, 0.4% fructose and 0.8% casamino acids unless otherwise stated) supplemented with the appropriate antibiotics and grown overnight at 37 °C with aeration in a shaking incubator. In the morning, cultures were diluted by 1:4 with fresh M9 in 1 cm cuvettes to measure the OD_700_ of each sample in the spectrophotometer (WPA Biowave II). Each sample was diluted to OD_700_ 0.01 in 2 mL of fresh M9 supplemented with the appropriate antibiotics (kanamycin: 50 μg/mL; ampicillin: 100 μg/mL; spectinomycin: 50 μg/mL; streptomycin: 100 μg/mL; chloramphenicol: 34 μg/mL; tetracycline: 10 μg/mL). For dBroccoli measurements, DFHBI-1T dye (Bio-Techne) was added to a final concentration of 100 μM unless otherwise stated. 200 μL of each sample was then transferred into a sterile 96-well plate and covered with a Breath-Easy membrane (Sigma). The plate was placed into a microplate reader and incubated at 37 °C for 1 h (Tecan Spark: Double orbital shaking, 1.5 mm amplitude). Measurements of OD700 and fluorescence (sfCFP: 430(20) nm ex./465(35) nm em.; sfYFP/sfGFP/dBroccoli: 485(20) nm em./535(25) nm em.; mScarlet-I/mRFP1/mCherry/mKate: 560(20) nm ex./ 620(20) nm em.) were taken every 15 min. One hour into the incubation, we briefly removed the microplate from the plate-reader, carefully removed the Breath-Easy membrane and added the appropriate inducers to each well in the appropriate concentrations. We then covered the microplate with a new Breath-Easy Membrane and introduced it back into the plate-reader, and set this time point as “time 0” by creating a “new plate” in the experiment. OD700 and fluorescence were taken every 15 min. Cells were grown for 6–24 h, depending on the experiment. Data were exported into an Excel Spreadsheet and analysed using MATLAB.

OD and fluorescence raw data were first subtracted from the mean of M9 media well replicates over time. Data were then smoothed using MATLAB smoothing spline function (smoothing parameter: 0.8648426188005848). Growth rate and fluorescence production rate per cell were calculated as described in Ceroni et al.^90^: Growth rate at *t*2 = (ln(OD(*t*3)) − ln(OD(*t*1)))/(*t*3 − *t*1), GFP production rate at *t*2 = ((Total GFP(*t*3) − Total GFP(*t*1))/(*t*3 − t1))/OD(*t*2), and mCherry production rate at *t*2 = ((Total mCherry(*t*3) − Total mCherry(*t*1))/(*t*3 − *t*1))/OD(*t*2), where *t*1 corresponds to 0 min after induction, *t*2 to 15 min after induction and *t*3 = 30 min after induction. Rates are reported at the time of maximum growth rate of the host cell (*μ*_max_). Mean values and standard deviations were calculated from the three biological replicates of each sample. For figures representing input-output response curves, we used the nlinfit and fitnlm MATLAB functions to fit a four-parameter logistic regression model (4PL model) to the data and determine the R-squared and *p*-value of the model:

*a* + ((*b* − *a*)/(1 + (*x*/*c*)^d^)).

### Flow cytometry

Flow cytometry was used to measure coculture composition by counting the number of red fluorescent cells and non-fluorescent cells. Cell fluorescence was measured in the Attune NxT (Thermo Scientific) flow cytometer using the following parameters: FSC 660 V, SSC 500 V, violet laser VL1 (405 nm ex./440(50) nm em.) 420 V, blue laser BL1 (488 nm ex./530(30) nm em.) 450 V, yellow laser YL2 (561 nm ex./620(15) nm em.) 560 V. The following thresholds were used: AND FSC 0.5 × 1000, AND SSC 4 × 1000. Five mixing cycles were used between each sample. Ten thousand cells were collected for each sample, and data were analysed using FlowJo and plotted with MATLAB. In FlowJo, we first gated *E. coli* cells from dust and cell debris by plotting SSC-A vs FSC-A. Then we gated the *E. coli* population to find the singlets population by plotting FSC-H vs FSC-A. Finally, the red and non-red singlet populations were determined by plotting BL1-H vs YL2-H. The gating strategy is demonstrated in Supplementary Fig. 29. For graphs representing fluorescence intensity, points represent the mean of the median fluorescence of three biological samples. Deactivation percentage of the STAR-based comparator was calculated using the following formula: deactivation % =0 ((*F*_STAR_ − *F*_STAR,anti-STAR_)/(*F*_STAR_ − *F*_neg._))*100, where *F* represents the average median fluorescence obtained by the flow cytometer of three biological samples. F_STAR_ is the fluorescence of the comparator when STAR is expressed, F_STAR,antiSTAR_ is the fluorescence of the comparator when both STAR and anti-STAR are expressed, and F_neg._ is the fluorescence of the comparator when neither STAR nor anti-STAR are expressed.

### Quorum-sensing sender-receiver assay

Sender strains and receiver strains were inoculated in 1 mL of rich M9 medium supplemented with the appropriate antibiotics in a 2 mL deep-well 96-well plate (VWR), covered with a “Breathe Easier” membrane (Sigma), and incubated overnight at 30 °C in a plate shaker incubator (Infors HT Multitron) shaking at 700 rpm overnight. In the morning, sender strains were diluted to 1:4 in fresh M9 medium in a 1 cm cuvette, and OD_700_ was measured in the spectrophotometer. Cells were diluted to an OD_700_ 0.05 in 1 mL of fresh M9 medium with antibiotics in a new 2 mL deep-well 96-well plate, covered with a “Breathe Easier” membrane (Sigma), and incubated at 700 rpm, 30 °C in the plate-shaker incubator. Every hour for 6 h, the deep-well plate was taken out of the incubator, and new wells were inoculated with 1 mL of OD_700_ 0.05 of each culture. Receiver strains were diluted 1:200 in fresh M9 with antibiotics in a new deep-well plate and incubated at 700 rpm, 30 °C in the plate-shaker incubator. At 6 hours, 250 μL of each sample from the sender strains deep-well plate was transferred to 1 cm cuvette to measure OD_700_ of each sender strain culture. The sender’s deep-well plate was then centrifuged at 3434×*g* (4000 rpm) for 5 min (Centrifuge Eppendorf 5810 R), and the supernatants were transferred by pipetting into a new 2 mL deep-well plate, diluted 1:1 with fresh M9 with antibiotics, and the pellets were discarded. The OD_700_ of the receiver strains were measured in the spectrophotometer by diluting 1:2 with fresh M9 in 1 cm cuvettes. Receiver strains were then diluted to OD_700_ 0.01 in 1 mL of the diluted sender strains’ supernatants. In total, 200 μL of each sample was transferred to a clear flat-bottom 96-well plate, and inducers (and DFHBI-1T dye if dBroccoli was expressed) were immediately added to the appropriate wells. The microplate was covered with a “Breathe-Easy” membrane (Sigma) and incubated in the Tecan Spark for 12 h. OD and fluorescence were measured every 15 min as previously described in the above “plate-reader assay” section.

### Coculture assay

Cells carrying the open-loop and closed-loop versions of the composition controller circuits were grown as monocultures in 1 mL of rich M9 medium supplemented with the appropriate antibiotics in 2 mL 96-well deep-well plates (VWR) at 30 °C in a plate shaker incubator (Infors HT Multitron) shaking at 700 rpm overnight. In the morning, cells were diluted 1:4 with fresh rich M9 medium and transferred to 1 cm cuvettes to measure OD700 in the spectrophotometer. Subsequently, each sample was diluted to an OD_700_ of 0.01 in a volume of 2 mL (adjusted according to the experiment’s needs). In a transparent flat-bottom 96-well plate (Costar), which we will refer to as the “experiment 96-well plate”, 100 μL of each monoculture that will compose the two-strain coculture is mixed in the appropriate wells (total volume of 200 μL in each well). Cocultures and monoculture controls were immediately induced with the appropriate inducers. The 96-well plate was covered with its plastic lid, inserted into a Tecan Spark plate-reader and incubated at 37 °C with shaking. OD_700_ and fluorescence were measured every 15 min. Every 2 h for 12 h and at 24 h, we paused the TECAN Magellan programme running the plate-reader experiment and, under sterile conditions, we transferred 1–6 μL of cells (0–2 h: 6 μL; 4–6 h: 3 μL; 8–10 h: 1 μL, 12–24 h: 0.5 μL) into a clear round-bottom 96-well plate (Costar) pre-filled with 200 μL of 1× PBS (Sigma) supplemented with tetracycline (10 μg/mL). We call this plate the “flow plate,” as it will be used to measure cell fluorescence in the flow cytometer to determine coculture composition. The “experiment 96-well plate” was then covered with its lid and placed back into the plate-reader, where the TECAN Magellan programme was resumed. The “flow plate” was then immediately stored on ice in the fridge at 4 °C. When the “flow plate” was entirely filled with coculture samples diluted in PBS, it was then run in the flow cytometer as previously described in the above “Flow cytometry assay” section.

### β-Carotene assay

The protocol was adapted from Borkowski et al^122^. For quantification of β-Carotene production, cells carrying the β-Carotene producing plasmids and controls were grown in 5 mL of rich M9 media in a 15 mL culture tube overnight at 37 °C in shaking conditions. In the morning, cells were diluted 1:4 in fresh M9 media and 1 mL of diluted cultures was transferred to 1 cm cuvettes to measure OD_700_ in the spectrophotometer. Each sample was diluted to an OD_700_ of 0.01 in a volume of 5 mL in a sterile 15 mL culture tube and induced with the appropriate inducers. After 6 h of growth at 37 °C in the shaking incubator, 0.5 mL of each culture was diluted 1:1 with fresh rich M9 media and its OD_700_ was measured in the spectrophotometer. The remaining 4.5 mL of each culture were spun down at 3434×*g* (4000 rpm) for 10 min (Centrifuge Eppendorf 5810 R), and the remaining supernatant was discarded by pipetting. Pellets were resuspended in 300 μL of acetone in 1.5 mL Eppendorf tubes, homogenised by vortexing for 10 min and incubated at 55 °C (Eppendorf ThermoMixer C) for 15 min. Tubes were centrifuged for 1 min at 9391×*g* (10,000 rpm) (Thermo Scientific Heraeus Fresco 17 Centrifuge). In total, 100 μL of the supernatants were collected by pipetting and transferred to a new 1.5 mL Eppendorf tube. A volume of 100 μL of water was added to the 100 μL of supernatants in each tube and mixed by pipetting. The total 200 μL was then transferred to a clear flat-bottom 96-well plate (Costar), and OD_450_ of the microplate was measured in the plate-reader (Tecan Spark). To compare the production of β-Carotene between the different samples, the OD_450_ of each sample was divided by its corresponding OD_700_. We note that β-Carotene does not absorb at OD_700_.

### Statistics and reproducibility

No statistical method was used to predetermine sample size. At least three biological replicates were performed for all experiments, which were sufficient to ensure consistent and reproducible results supporting the conclusions of this study. No data were excluded from the analyses. All experimental claims were tested in independent biological replicates performed on the same day, and results were found to be consistent across replicates. The experiments were not randomised. Bacterial colonies were randomly selected from freshly transformed cells, where a single colony represents one biological replicate. The investigators were not blinded to allocation during experiments and outcome assessment. Blinding was not considered necessary, as data collection relied on objective measurements, including flow cytometry and microplate reader assays.

### Reporting summary

Further information on research design is available in the Nature Portfolio Reporting Summary linked to this article.

## Supplementary information

Supplementary Information

Description of Additional Supplementary Files

Supplementary Data 1

Supplementary Data 2

Reporting Summary

Transparent Peer Review file

## Source data

Source Data

## Data Availability

Plasmid maps (Genbank files) are publicly available on Zenodo^141^. Source data are provided with this paper.

## Code availability

The Simbiology model is publicly available on Zenodo^141^.
