# Supplementary material for: Burden-aware feedback control of microbial consortia
Source: Nat Commun. 2026 May 6;17:6100. doi: 10.1038/s41467-026-72389-6 (PMC13357841; doi:10.1038/s41467-026-72389-6)
Supplement: Supplementary file 1 — Supplementary Information [file 41467_2026_72389_MOESM1_ESM.pdf]

Supplementary information for:

Burden-aware feedback control of microbial consortia

Alice Boo [1,2], Harman Mehta [1,2], Rodrigo Ledesma-Amaro [1,2*†], Guy-Bart Stan [1,2*†]

[1] Imperial College Centre for Excellence in Synthetic Biology, Imperial College London, SW7 2AZ, London, UK

[2] Department of Bioengineering, Imperial College London, SW7 2AZ, London, UK

[†] Joint supervision
[*] to whom correspondence should be addressed g.stan@imperial.ac.uk, r.ledesma-amaro@imperial.ac.uk

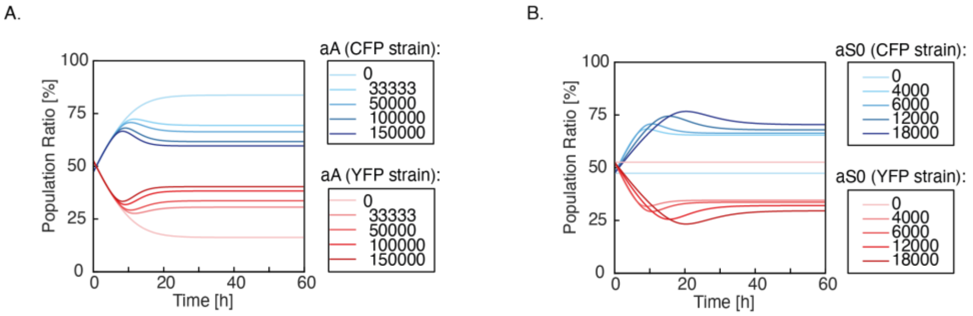

<u>Supplementary Figure 1:</u> **Simulations of the microbial composition.** Simulations of the mathematical model from **Supplementary Note 1** that describes the RNA-based comparator that controls growth rate through the burden caused by expressing VioB. (A) Tuning the anti-STAR rate of transcription. The rate of STAR transcription is set as aS0 = 6,000. When aA is set to 0, no anti-STAR is expressed in the cell, and as a result the growth controller is inactive. As the values of aA increase, more anti-STAR is expressed in the cells. As a result, more STAR is sequestered which prevents the expression of the burdensome proteins that slow down cellular growth rate. (B) Tuning the STAR transcription rate. The rate of anti-STAR transcription is set as aA = 200,000. When aS0 is set to 0, no burdensome protein is expressed in either the CFP or YFP strain of the microbial consortia, thus both strains grow at the same rate. However, as the transcription rate of STAR, as0, increases, each strain expresses more of their respective burdensome protein. As the burdensome proteins have different burden, they impact growth rate differently and the population ratio diverges from the initial inoculation ratio (50:50). The divergence from the initial inoculation ratio is more pronounced as the rate of STAR increases as it, in turn, increases production of the burdensome proteins. Simulations were performed in the MATLAB SimBiology toolbox.

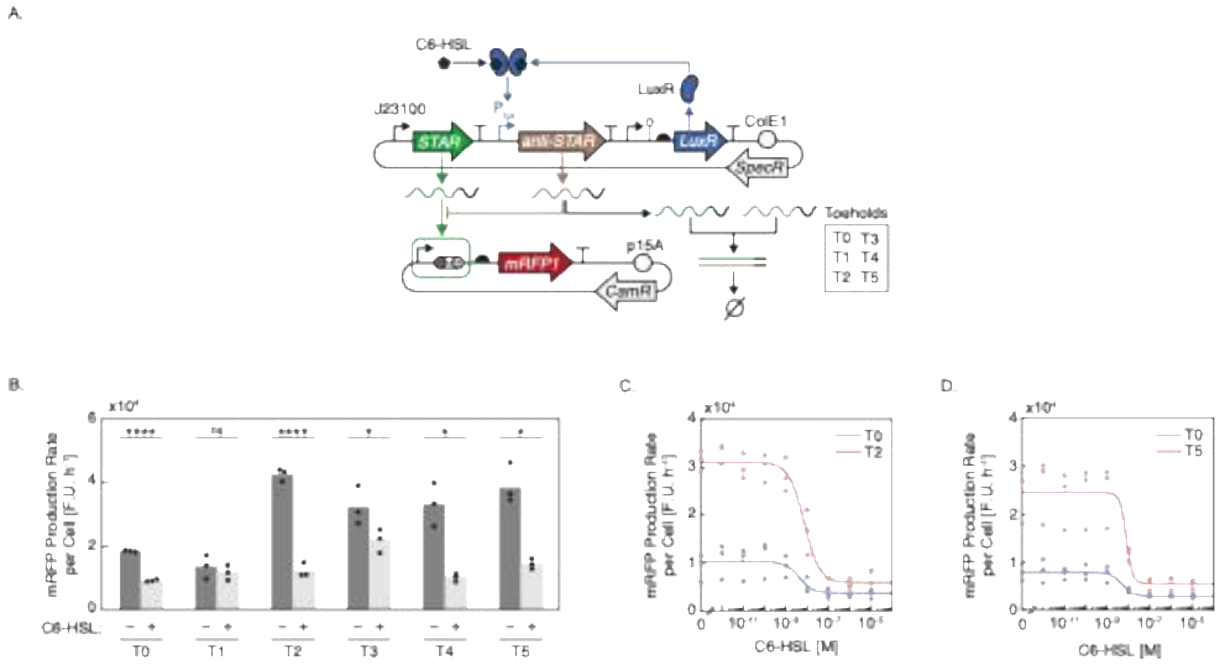

Supplementary Figure 2: **STAR-based comparator toehold library.** (A) Diagram of the J23100-pLux STAR-based comparator with the toehold library. The target plasmid pJBL5939 carries a single STAR target driving expression of mRFP. (B) Bar graph representing the mRFP production rate of the strains carrying the comparators with the toeholds T0, T1, T2, T3, T4 and T5. Plasmids pAB161, pAB232, pAB233, pAB234, pAB235, pAB236 are the plasmids carrying the comparator designs with toeholds T0 to T5 respectively. "-HSL" represents a concentration of 0 M while "+HSL" represents a concentration of 10^{-7} M of C6-HSL. (C) T0 and T2 comparators' normalised mRFP production rate per cell as a function of C6-HSL concentration. (D) T0 and T5 comparators' normalised mRFP production rate per cell as a function of C6-HSL concentration. Experiments were carried out in BW25113. Growth and fluorescence were monitored in a time-course plate-reader assay. Data points represent three biological replicates, the bars represent the means of these points, and the curves were fitted to the means of these points using MATLAB four-parameter nonlinear regression fit. Statistically significant differences were determined using two-tailed Student's t-test (**** represents $p<0.0001$, *** represents $p<0.001$, ** represents $p<0.01$, * represents $p<0.1$, ns represents not significant). Plasmids used in this figure are recorded in Supplementary Data 2 and strains in Supplementary Table 1. Source data are provided as a Source Data file.

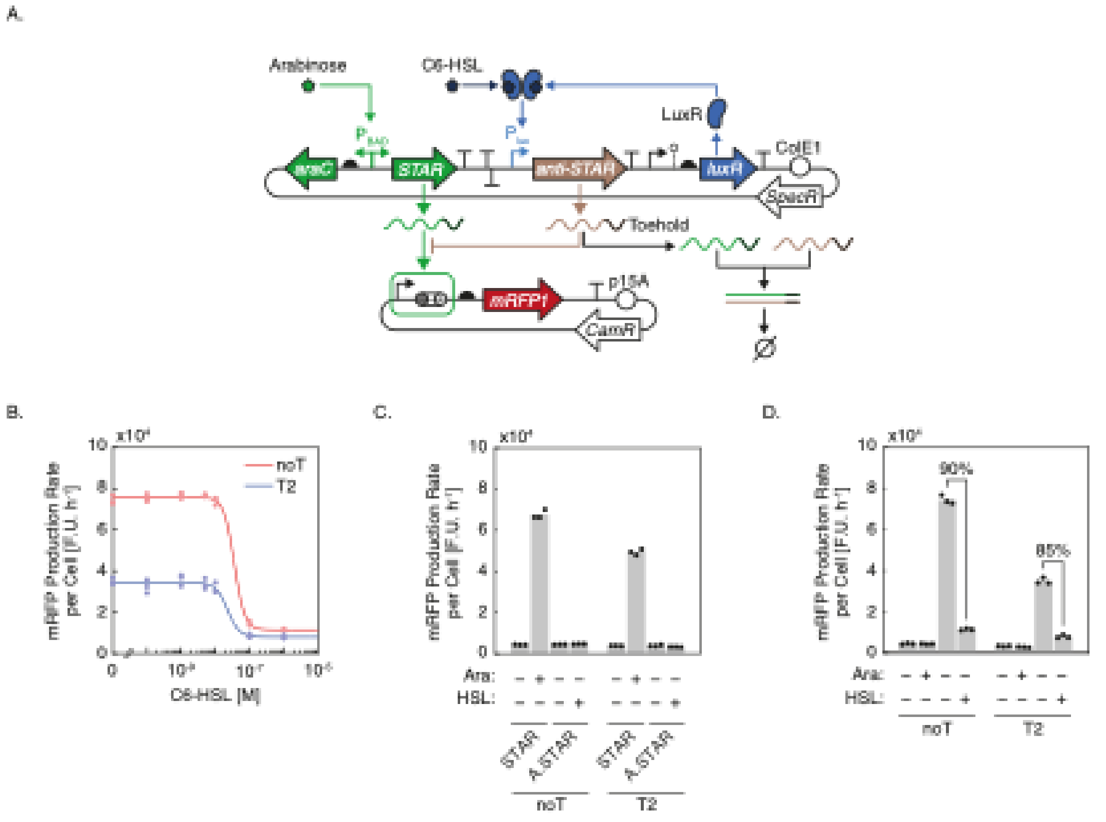

Supplementary Figure 3: Characterisation of the STAR-based comparator with and without toehold. (A) Diagram of the circuit used to compare the STAR-based comparator with and without toehold. DH10B carrying plasmids pJBL5939 and pAB317 was used to test the comparator design without a toehold (noT), and DH10B carrying pJBL5939 and pAB300 was used to test the comparator design with toehold T2. (B) NoT and T2 comparators' mRFP production rate per cell as a function of C6-HSL concentration. (C) mRFP production rate per cell of the STAR and anti-STAR controls. The STAR controls only carried STAR with (T2) or without toehold (noT) on the comparator plasmid and were induced by L-arabinose ("Ara-" for 0% and "Ara+" for 0.2%). The "A.STAR" controls only carried anti-STAR with (T2) or without toehold (noT) on the comparator plasmid and were induced by C6-HSL ("HSL-" for 0 M and "HSL+" for 10^{-6} M). (D) The deactivation percentage of the NoT and T2 comparators was calculated as $(mRFP_{[+Ara][-HSL]} - mRFP_{[+Ara][+HSL]})/(mRFP_{[+Ara][-HSL]} - mRFP_{[-Ara][-HSL]})$, where mRFP is the mRFP production rate per cell. The comparators were induced with L-arabinose ("Ara-" for 0% and "Ara+" for 0.2%) and C6-HSL ("HSL-" for 0 M and "HSL+" for 10^{-6} M). Growth and fluorescence were monitored in a time-course plate-reader assay. Data points represent three biological replicates, the bars represent the means of these points, and the curves were fitted to the means of these points using MATLAB four-parameter nonlinear regression fit. Plasmids used in this figure are recorded in Supplementary Data 2 and strains in Supplementary Table 1. Source data are provided as a Source Data file.

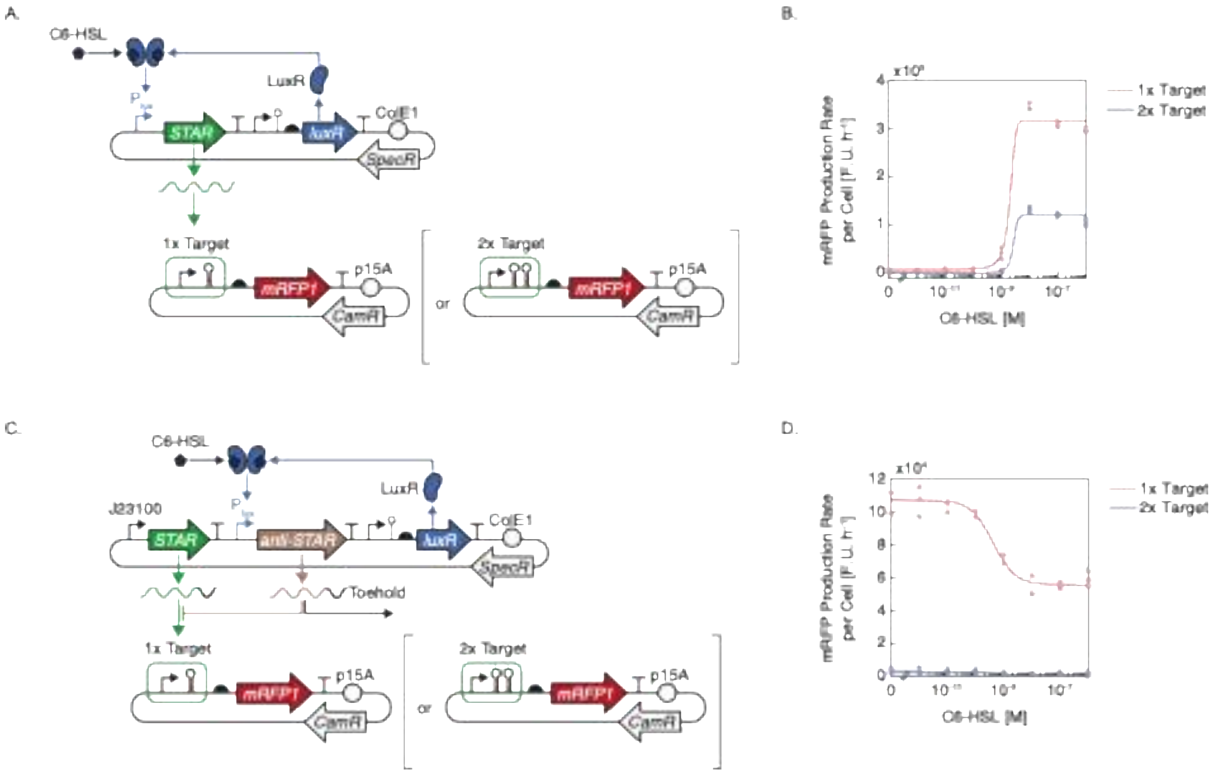

<u>Supplementary Figure 4:</u> **Effect of STAR-double-target.** (A) Diagram of the plasmids used to characterise the effect of the STAR-double-target on mRFP expression. BW25113 carrying plasmid pAB127 expressing STAR and with either plasmids pJBL5939 or pAB262 carrying one- or two-STAR target sites respectively. (B) Normalised mRFP production rate per cell as a function of C6-HSL concentration when the gene of interest (mRFP) is controlled by either a single-STAR target or a double-STAR target. (C) Diagram of circuit used to characterise the effect of the STAR-double-target on the behaviour of the STAR-based comparator. BW25113 carrying plasmid pAB161 expressing the comparator with toehold T0 and with either pJBL5939 or pAB262 carrying one- or two-STAR target sites respectively. (D) Normalised mRFP production rate per cell as a function of C6-HSL concentration when the STAR-based comparator's gene of interest (mRFP) is controlled by either a single-STAR target or a double-STAR target. Growth and fluorescence were monitored in a time-course plate-reader assay. Data points represent three biological replicates, and the curves were fitted to the means of these points using MATLAB four-parameter nonlinear regression fit. Plasmids used in this figure are recorded in Supplementary Data 2 and strains in Supplementary Table 1. Source data are provided as a Source Data file.

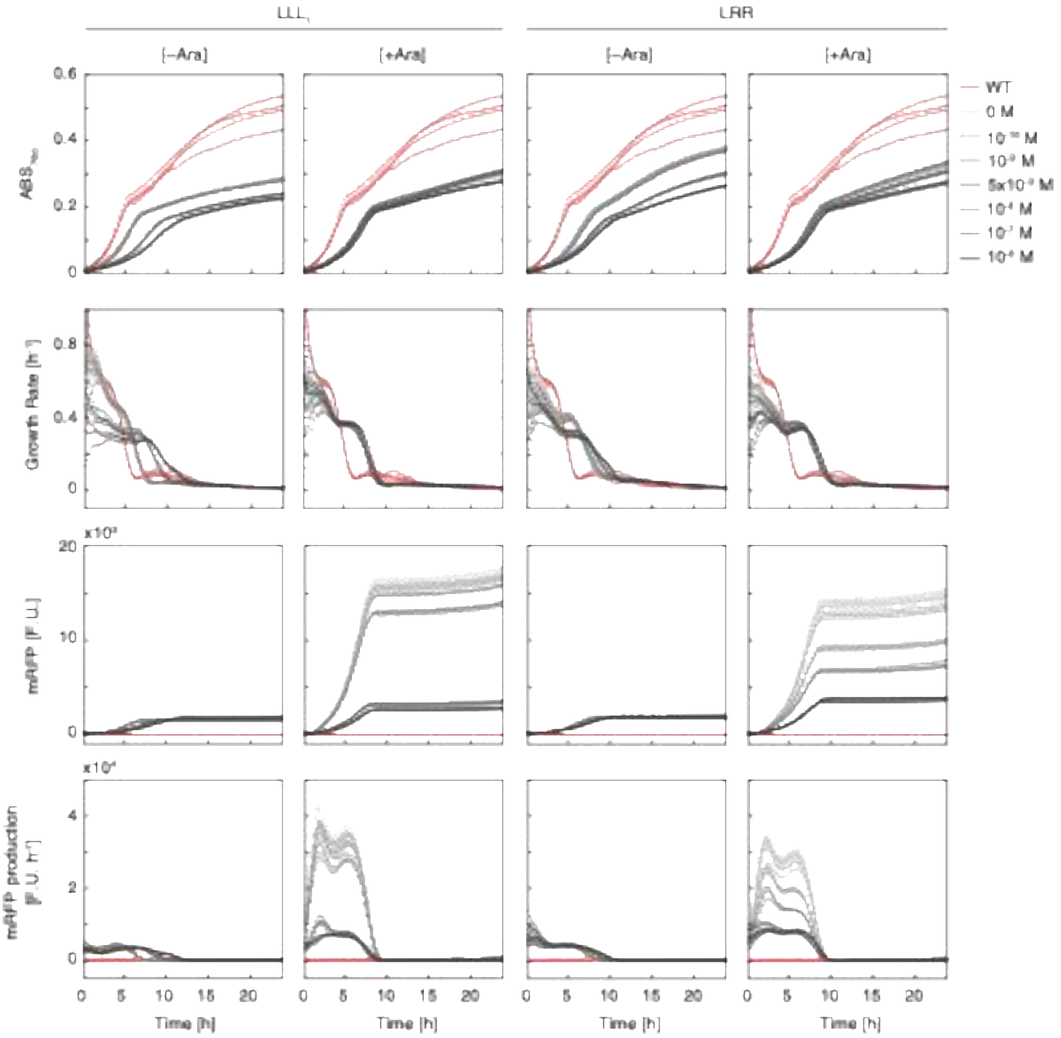

<u>Supplementary Figure 5:</u> Growth and mRFP production of the LLL_1 and LRR STAR-based comparators. OD, instantaneous growth rate, mRFP fluorescence and mRFP production rate per cell of the LLL_1 and LRR comparators from Figure 2B when induced with 0% of L-arabinose (-Ara) or 0.2% of L-arabinose (+Ara). The LLL_1 and LRR comparators are also induced with a range of C6-HSL or pC-HSL concentrations, respectively (0 M, 10^{-10} M, 10^{-9} M, $5x10^{-9}$ M, 10^{-8} M, 10^{-7} M, 10^{-6} M). Growth and fluorescence were monitored in a time-course plate-reader assay. The data points represent 3 biological replicates, and the curves were fitted to the means of these points using a smoothing spline. Plasmids used in this figure are recorded in Supplementary Data 2 and strains in Supplementary Table 1. Source data are provided as a Source Data file.

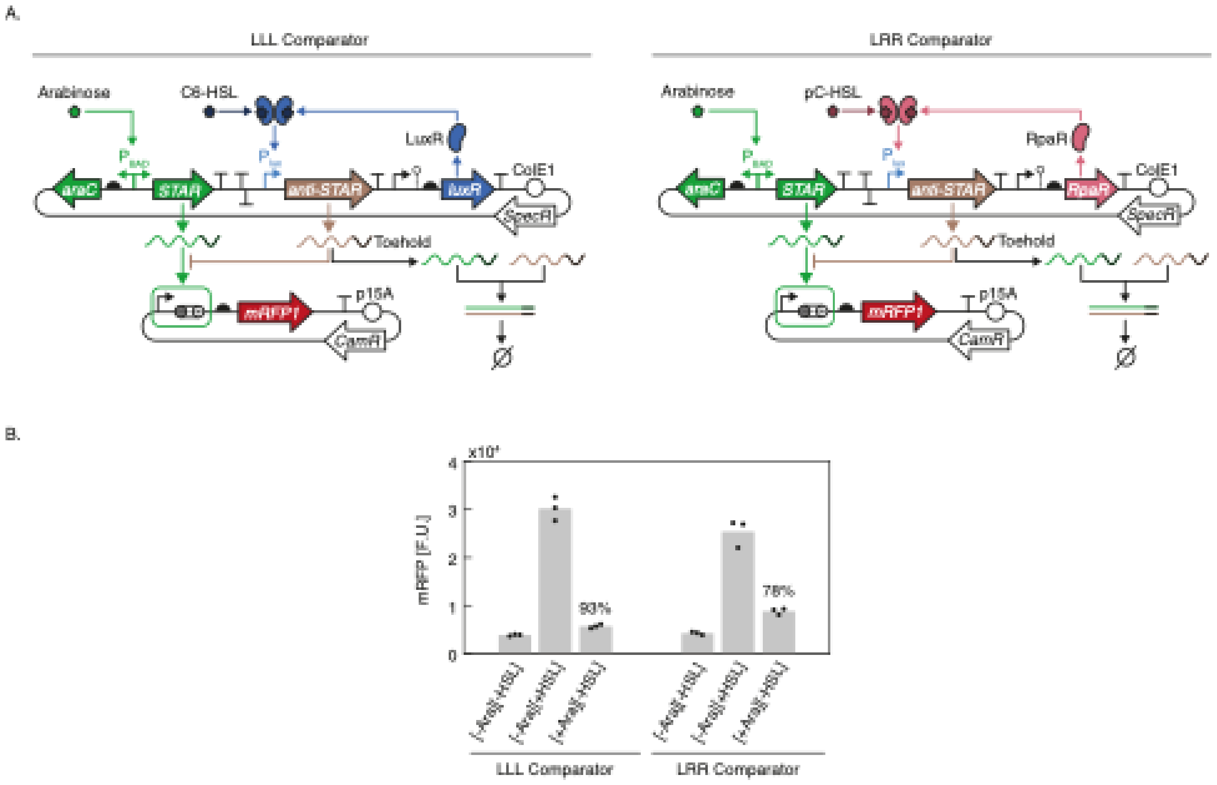

<u>Supplementary Figure 6:</u> **Deactivation of the LLL_1 and LRR STAR-based comparators.** (A) Design of the LLL comparator (DH10B carrying plasmids pJBL5939 and pAB300) and LRR comparator (DH10B carrying plasmids pJBL5939 and pAB401). (B) The deactivation percentage is calculated for the LLL and LRR STAR-based comparators. The comparator is externally induced with three conditions: (1) "[-Ara][-HSL]" represents induction with 0% of L-arabinose and 0 M of C6-HSL or pC-HSL for the LLL or LRR comparator respectively; (2) "[+Ara][-HSL]" represents induction with 0.2% of L-arabinose and 0 M of C6-HSL or pC-HSL for the LLL or LRR comparator respectively; (3) "[+Ara][+HSL]" represents induction with 0.2% of L-arabinose and 10^{-7} M of C6-HSL or pC-HSL for the LLL or LRR comparator, respectively. The deactivation percentage is calculated as $((F_{STAR} - F_{STAR,anti\text{-}STAR})/(F_{STAR} - F_{neg.})) \times 100\%$, where F_{STAR} corresponds to the fluorescence of the comparator when only STAR is expressed ("[+Ara][-HSL]"), $F_{STAR,anti\text{-}STAR}$ corresponds to the fluorescence of the comparator when both STAR and anti-STAR are expressed ("[+Ara][+HSL]"), and $F_{neg.}$ corresponds to the fluorescence of the comparator when neither STAR nor anti-STAR are expressed ("[-Ara][-HSL]"). Fluorescence was measured by flow cytometry. The data points represent 3 biological replicates, and the bars represent the means of these points. Plasmids used in this figure are recorded in Supplementary Data 2 and strains in Supplementary Table 1. Source data are provided as a Source Data file.

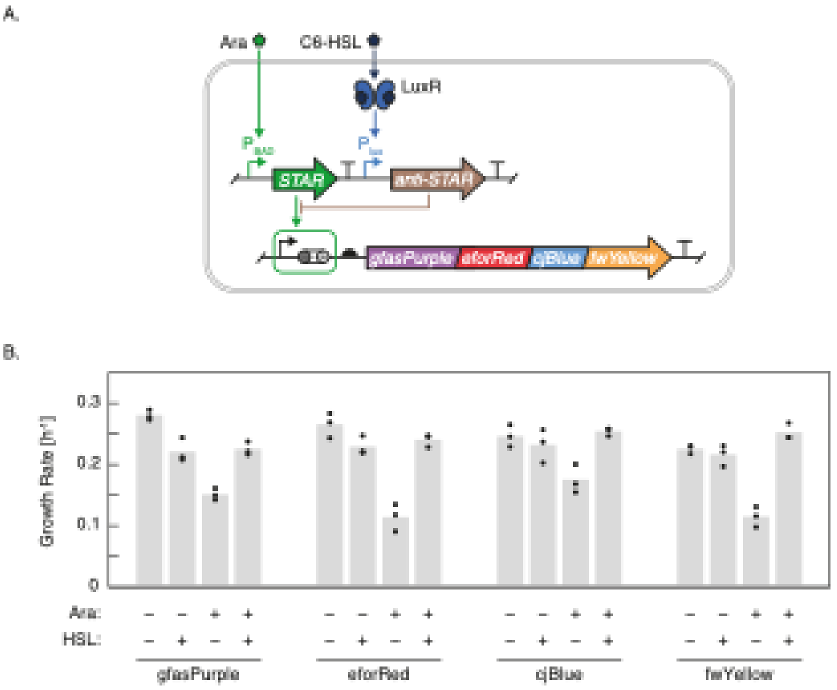

<u>Supplementary Figure 7:</u> A burden-based control system using the STAR-based comparator controlling growth rate through the expression of chromoproteins. (A) The LLL comparator is linked to production of four different chromoproteins to control growth rate. DH10B-GFP is carrying the pAB300 plasmids and pAB367 (gfasPurple), pAB368 (eforRed), pAB369 (cjBlue) or pAB370 (fwYellow) plasmids. (B) Maximum growth rate of the comparator controlling the expression of gfasPurple, eforRed, cjBlue or fwYellow. Two concentrations of L-arabinose were used: 0% ("Ara−") and 0.2% ("Ara+"), and two concentrations of C6-HSL used were: 0 M ("HSL−") and 10^{-7} M ("HSL+"). Growth was monitored in a time-course plate-reader assay. The data points represent 3 biological replicates, and the bars represent the means of these points. Plasmids used in this figure are recorded in Supplementary Data 2 and strains in Supplementary Table 1. Source data are provided as a Source Data file.

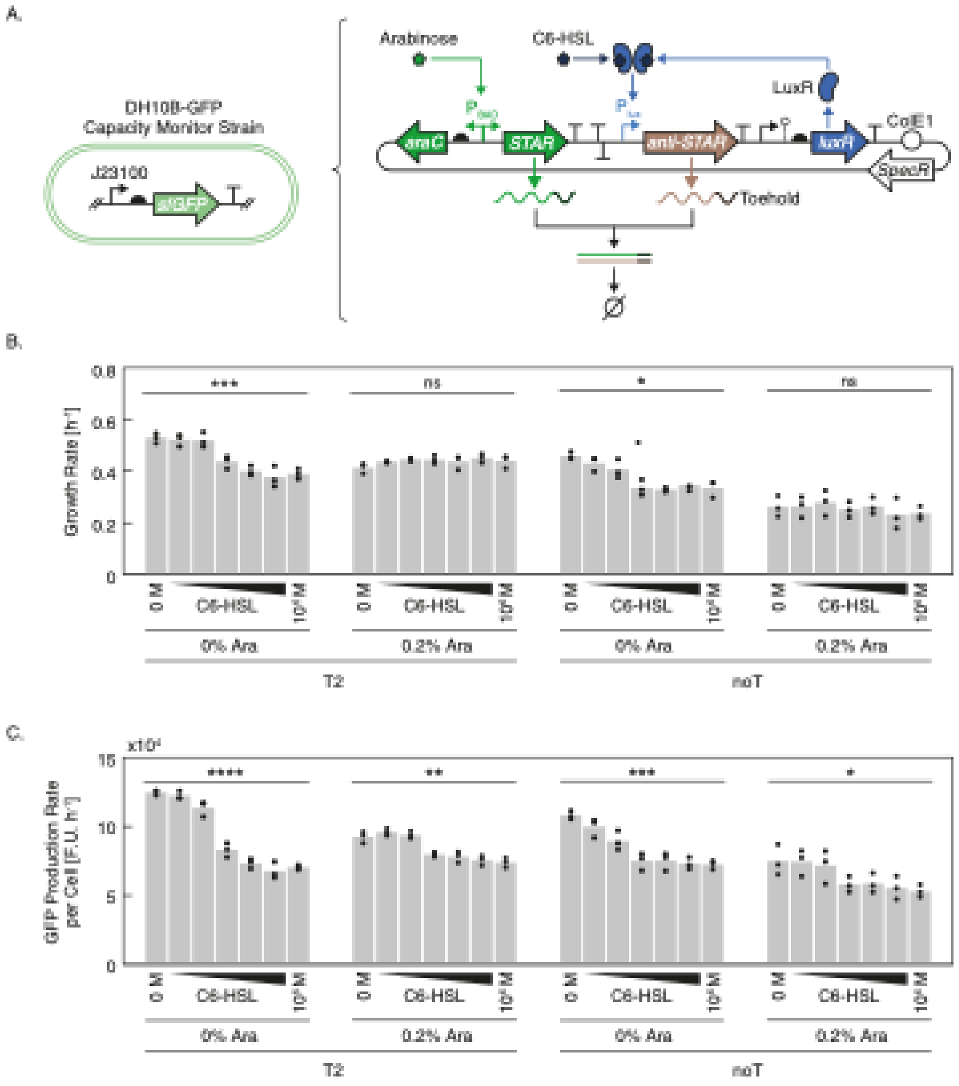

Supplementary Figure 8: Burden characterisation of the STAR-based comparator with and without toehold. (A) Diagram of the comparator plasmid tested in the DH10B-GFP strain carrying the GFP capacity monitor to assess the burden caused by expression of STAR and anti-STAR. STAR expression is enabled by L-arabinose, while anti-STAR expression is enabled by C6-HSL. DH10B-GFP carrying plasmid pAB300 was used to study the impact of expressing the comparator with toehold T2 and DH10B-GFP carrying plasmid pAB317 was used to study the impact of expressing the comparator without toehold (noT). (B) Growth rate of the comparator plasmids with and without toehold. (C) GFP capacity is the GFP production rate per cell of the DH10B-GFP capacity monitor strain transformed with the comparator plasmids with and without toehold. Two concentrations of L-arabinose were used: 0% and 0.2%. The concentrations of C6-HSL used were: 0 M, 10^{-10} M, 10^{-9} M, 5×10^{-9} M, 10^{-8} M, 10^{-7} M, 10^{-6} M. Growth and fluorescence were monitored in a time-course plate-reader assay. The data points represent 3 biological replicates, and the bars represent the means of these points. Statistically significant differences were determined using two-tailed Student's t-test (**** represents $p<0.0001$, *** represents $p<0.001$, ** represents $p<0.01$, * represents $p<0.1$, ns represents not significant). Plasmids used in this figure are recorded in Supplementary Data 2 and strains in Supplementary Table 1. Source data are provided as a Source Data file.

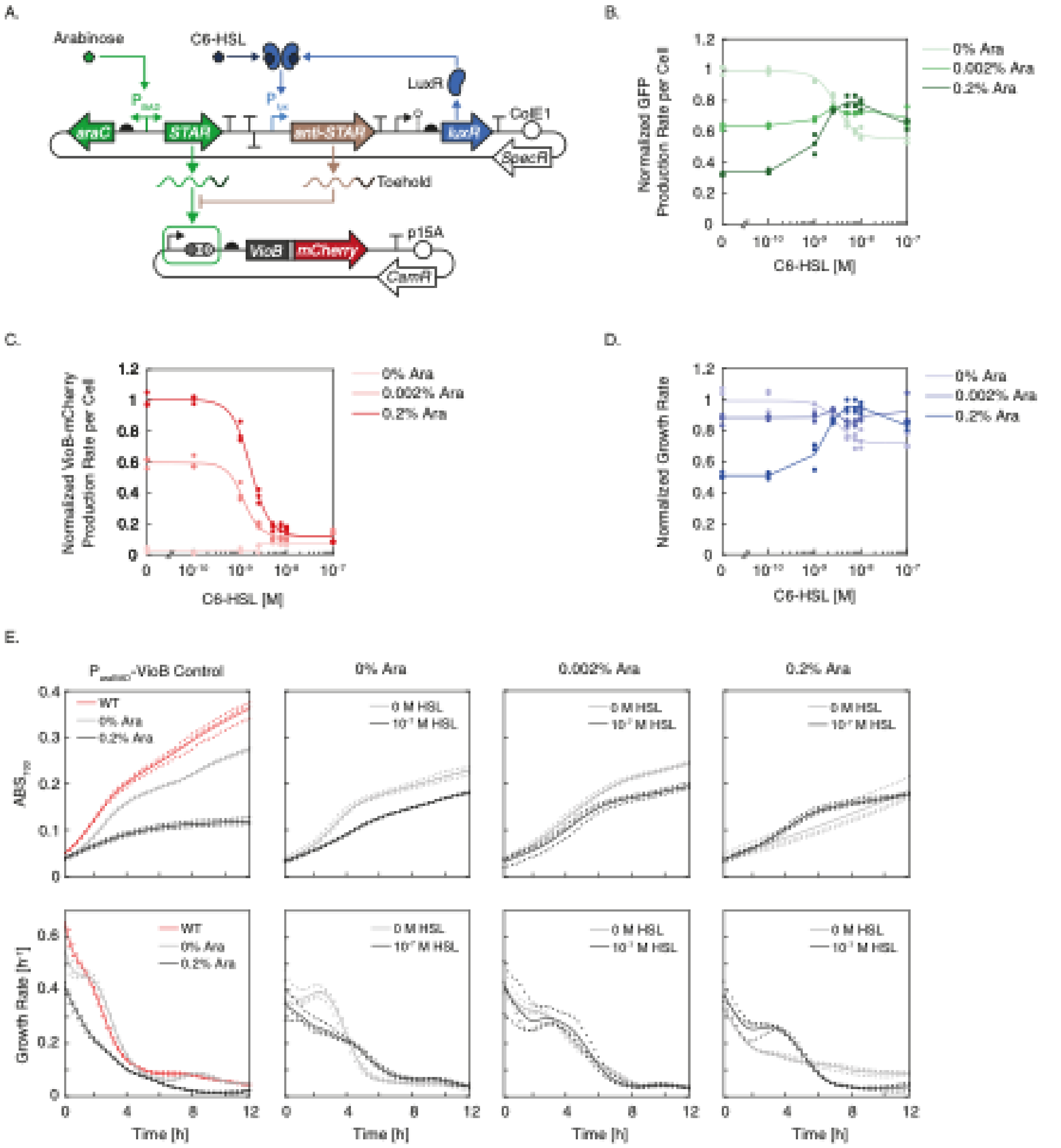

<u>Supplementary Figure 9:</u> A burden-based control system using the STAR-based comparator controlling growth rate through VioB-mCherry expression. (A) The LLL comparator is linked to VioB-mCherry production to control growth rate. DH10B-GFP is carrying plasmids pAB300 and pAB517. (B) Effect of increasing C6-HSL concentration on GFP capacity (GFP production rate per cell) for three concentrations of L-arabinose (0%, 0.002%, and 0.2%). The system was induced with a range of C6-HSL concentration from 0 M to 10^{-7} M. GFP capacity was normalised to the GFP production rate in the 0% L-arabinose and 0 M C6-HSL condition. (C) Effect of increasing C6-HSL concentration on VioB-mCherry production rate per cell for three concentrations of L-arabinose (0%, 0.002%, and 0.2%). VioB-mCherry expression was normalised to the VioB-mCherry production rate per cell in the 0.2% L-arabinose and 0 M C6-HSL condition. (D) Effect of increasing C6-HSL concentration on growth rate for three concentrations of L-arabinose (0%, 0.002%, and 0.2%). Growth rate was normalised with the growth rate in the 0% L-arabinose and 0 M C6-HSL condition. (E) OD and instantaneous growth rate of DH10B-

GFP carrying plasmid pAB271 expressing VioB-mCherry under the control of the araBAD promoter and DH10B-GFP carrying plasmids pAB300 and pAB517 from the previous panels. Growth and fluorescence were monitored in a time-course plate-reader assay. Data points represent three biological replicates, and the curves were fitted to the means of these points using MATLAB four-parameter nonlinear regression fit, when possible, otherwise the means were used to plot the lines. Plasmids used in this figure are recorded in Supplementary Data 2 and strains in Supplementary Table 1. Source data are provided as a Source Data file.

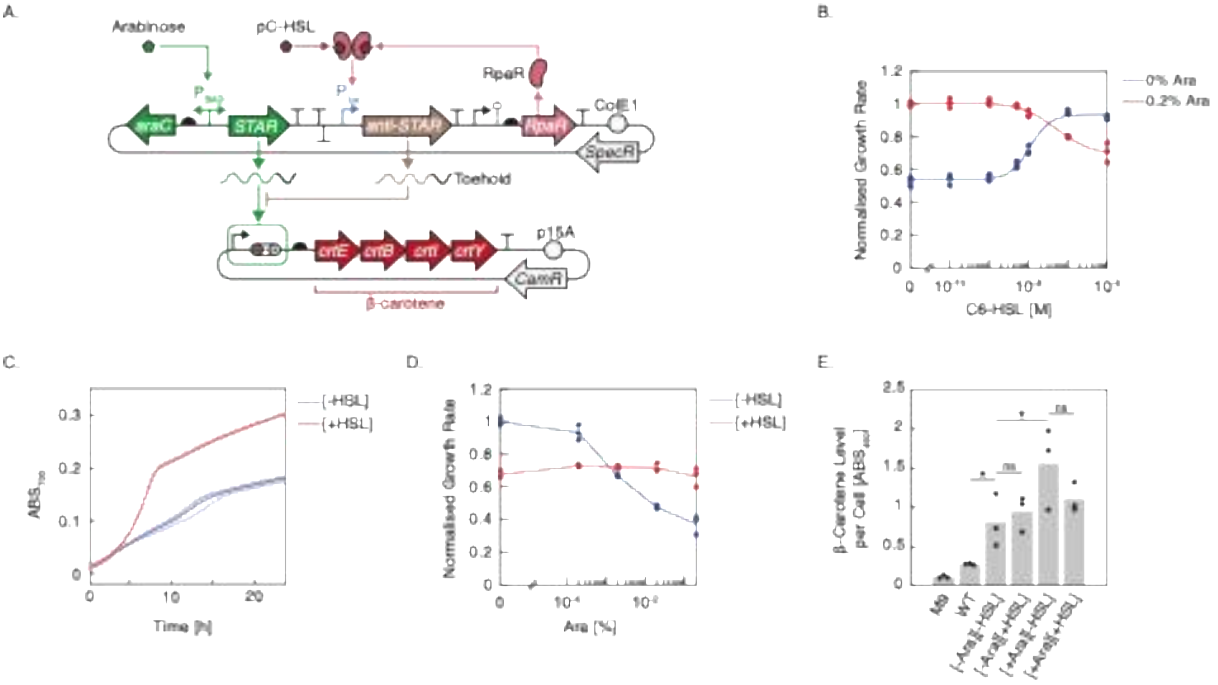

<u>Supplementary Figure 10:</u> A burden-based control system using the STAR-based comparator controlling growth rate through β-carotene expression. (A) The STAR-based comparator (with Toehold 2) based on the LRR quorum sensing system is linked to β-carotene production to control growth rate in DH10B carrying plasmids pAB401 and pAB550. β-carotene is produced through the expression of four enzymes: CrtE, CrtB, CrtI and CrtY organised in an operon as described by Borkowski et al. (2018) [1]. (B) Effect of increasing pC-HSL concentration on growth rate when the cells are induced with either 0% L-arabinose or 0.2% L-arabinose. Cells were induced with a range of pC-HSL from 0 M to 10^{-6} M. Growth rate was normalised with the growth rate of the system induced with 0% L-arabinose and 0 M of pC-HSL. (C) Effect of adding pC-HSL on the growth profile of the cells expressing maximal levels of STAR. Cells were induced with 0.2% L-arabinose. In the "-HSL" conditions, 0 M of pC-HSL was added to the media, while in the "+HSL" condition, 10^{-7} M of pC-HSL was added. (D) Effect of increasing L-arabinose concentration on normalised growth rate with 0 M or 10^{-7} M of pC-HSL was added to the media ("-HSL" and "+HSL" respectively). Growth rate was normalised with the growth rate of the system induced with 0% L-arabinose and 0 M of pC-HSL. (E) β-carotene production per cell measured as the absorbance at 450 nm divided by the absorbance at 700 nm at 24 h. The conditions refer to the concentration of inducer added to the media. "-Ara": 0% of L-arabinose, "+Ara": 0.2% of L-arabinose, "-HSL": 0 M of pC-HSL, "+HSL": 10^{-7} M of pC-HSL. Experiments were carried out in DH10B. Growth was monitored in a time-course plate-reader assay. Data points represent three biological replicates, the bars represent the means of these points, and the curves were fitted to the means of these points using MATLAB four-parameter nonlinear regression fit, when possible, otherwise the means were used to plot the lines. Statistically significant differences were determined using two-tailed Student's t-test (**** represents p<0.0001, *** represents p<0.001, ** represents p<0.01, * represents p<0.1, ns represents not significant). Plasmids used in this figure are recorded in Supplementary Data 2 and strains in Supplementary Table 1. Source data are provided as a Source Data file.

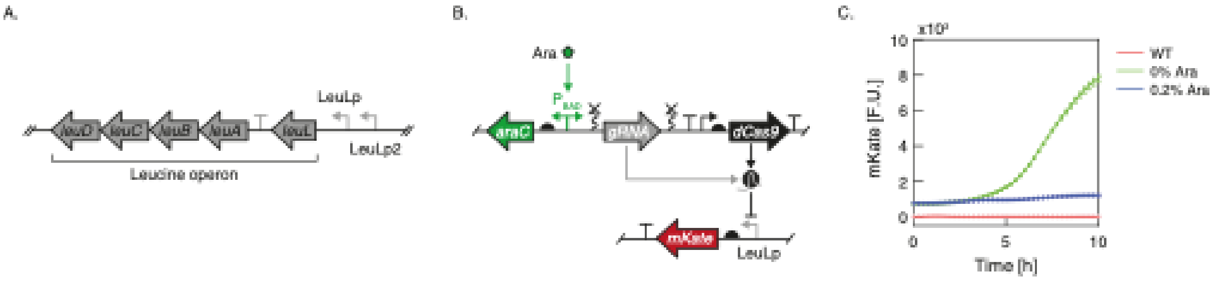

<u>Supplementary Figure 11:</u> **Designing an sgRNA to build a leucine knockdown using CRISPRi.** (A) Structure of the native leucine operon in BW25113. (B) gRNA expression is driven by the araBAD promoter. The gRNA sequence is flanked by the Hammerhead ribozyme (HH) on its 5' side and by the hepatitis delta virus ribozyme (HDV) on its 3' side. dCas9 is expressed using a weak constitutive promoter and RBS sequences. gRNA and dCas9 bind to form a CRISPRi complex that targets the LeuLp promoter controlling mKate expression. (C) Without L-arabinose, the LeuLp promoter drives mKate expression; with 0.2% L-arabinose, gRNA is produced and complexes with dCas9. The complex binds to the LeuLp promoter and inhibits its activity. Experiment was carried out in BW25113 carrying the pAB81 and B0034_mKate plasmids. Growth and fluorescence were monitored in a time-course plate-reader assay. Data points represent three biological replicates, and the curves were fitted to the means of these points using a smoothing spline. Plasmids used in this figure are recorded in Supplementary Data 2 and strains in Supplementary Table 1. Source data are provided as a Source Data file.

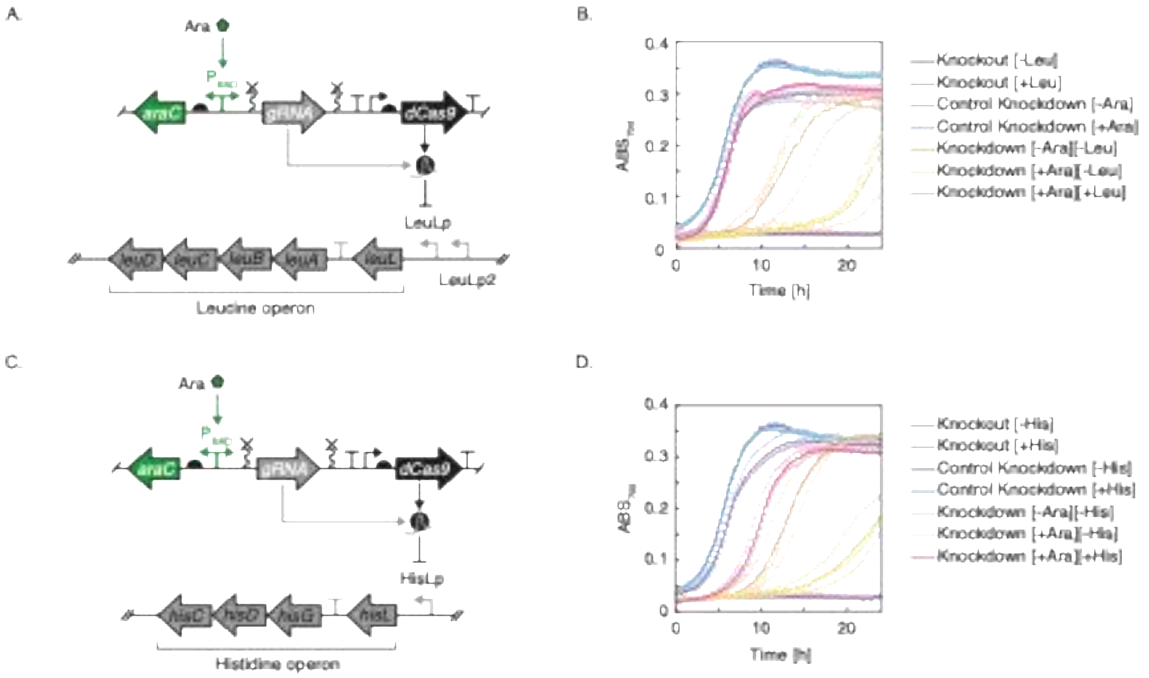

Supplementary Figure 12: **Targeting the LeuLp and HisLp promoters with CRISPRi**. (A) The construct knocking down leucine production in BW25113 carrying plasmid consists of a constitutively expressed dCas9, and an L-arabinose inducible gRNA targeting the genomic LeuLp promoter. (B) OD of the leucine knockdown, the ΔLeuB knockout from the Keio collection and the control knockdown expressing the "No Target" gRNA from Ceroni et al. [2] (BW25113 carrying plasmid pAB58). Conditions are as follows: "-Ara": induction with 0% of L-arabinose, "+Ara": induction with 0.2% of L-arabinose, "-Leu": M9 medium without casamino acids is supplemented with 0 µg/mL of L-leucine, "+Leu": M9 medium without casamino acids is supplemented with 40 µg/mL of L-leucine. (C) The construct knocking down histidine production in BW25113 carrying plasmid pAB60 consists of a constitutively expressed dCas9, and an L-arabinose inducible gRNA targeting the genomic HisLp promoter. (D) OD of the histidine knockdown, the ΔHisD knockout from the Keio collection and the control knockdown expressing the "No Target" gRNA [2]. Conditions are as follows: "-Ara": induction with 0% of L-arabinose, "+Ara": induction with 0.2% of L-arabinose, "-His": M9 medium without casamino acids is supplemented with 0 µg/mL of L-histidine, "+His": M9 medium without casamino acids is supplemented with 40 µg/mL of L-histidine. OD measurements were collected in a time-course plate-reader assay. Data points represent three biological replicates, and the curves were fitted to the means of these points using a smoothing spline. Plasmids used in this figure are recorded in Supplementary Data 2 and strains in Supplementary Table 1. Source data are provided as a Source Data file.

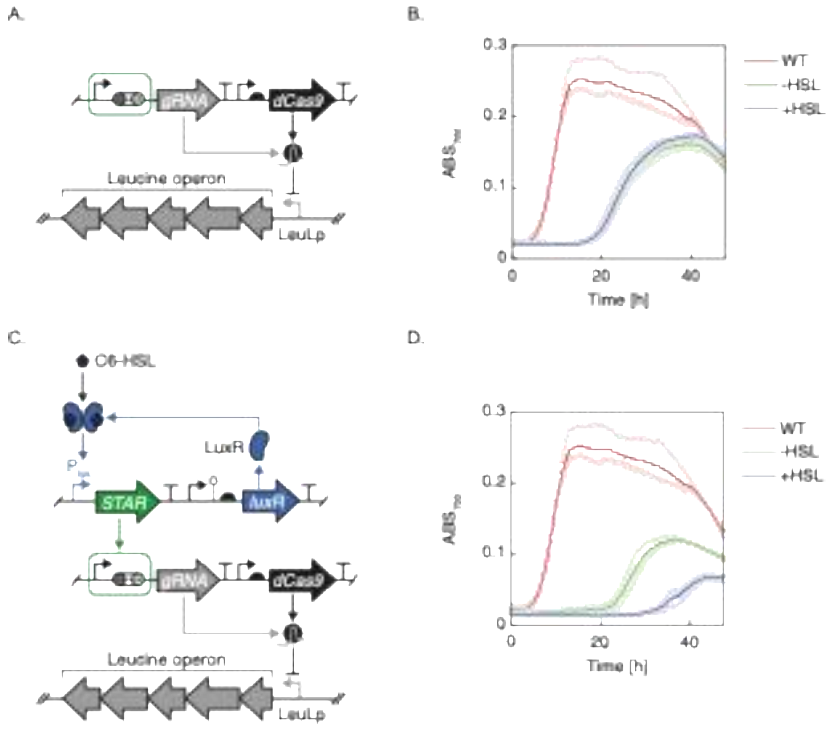

<u>Supplementary Figure 13:</u> **Using CRISPRi to knockdown the leucine operon**. (A) STAR target controls gRNA production targeting the LeuLp promoter in BW25113 carrying plasmid pAB96. In the presence of STAR, gRNA is transcribed and forms an inhibiting complex with dCas9 that targets to LeuLp promoter to repress expression of the leucine operon. (B) In the absence the plasmid expressing STAR, OD of the cells is inhibited by leaky transcription of gRNA. (C) The LLL STAR system controls gRNA production targeting the LeuLp promoter in BW25113 carrying plasmids pAB127 and pAB96. (D) In the presence of both plasmids, C6-HSL activates STAR expression, in turn increasing gRNA production, which consequently inhibits cellular growth and extends the lag-phase. "WT" shows OD of the wild-type BW25113 strain. "-HSL" and "+HSL" show growth of the cell containing the CRISPRi plasmid when 0 M and 10^{-7} M of C6-HSL is added to the M9 medium without casamino acids. Growth was monitored in a time-course plate-reader assay. Data points represent three biological replicates, and the curves were fitted to the means of these points using a smoothing spline. Plasmids used in this figure are recorded in Supplementary Data 2 and strains in Supplementary Table 1. Source data are provided as a Source Data file.

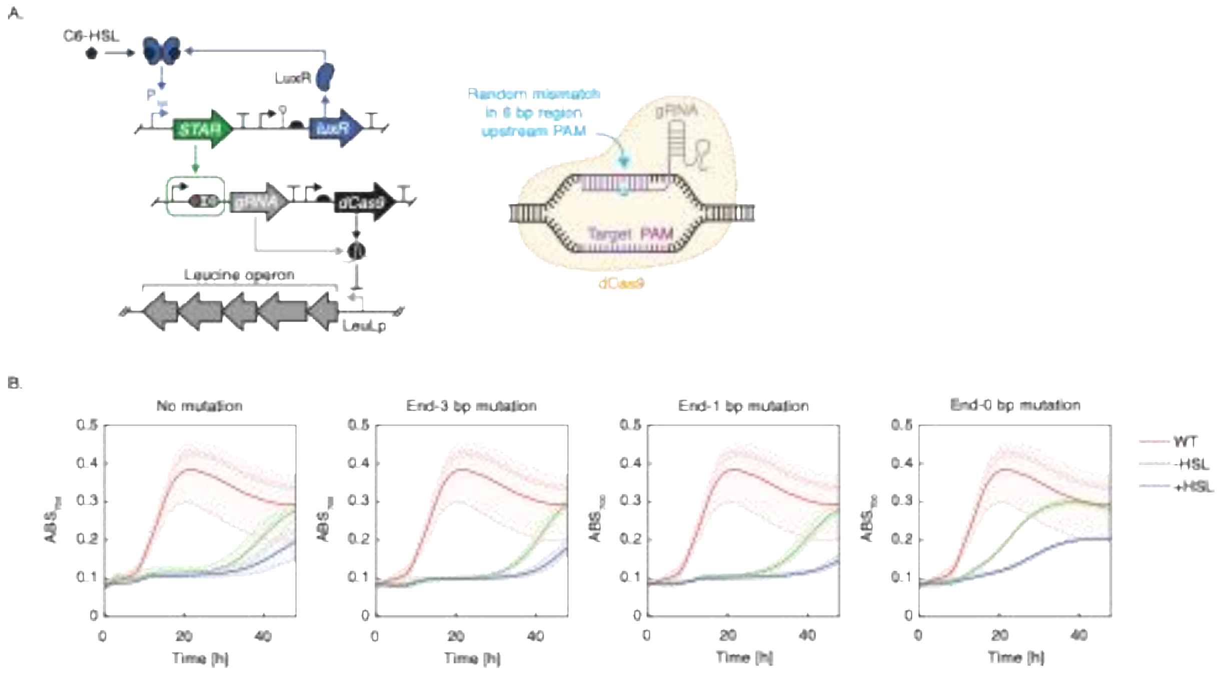

<u>Supplementary Figure 14:</u> **Mutations in the gRNA targeting LeuLp promoter.** (A) The mutated version of the gRNA targeting the LeuLp promoter is expressed under the control of STAR expressed in the presence of C6-HSL in BW25113 carrying plasmids pAB127 and either pAB96, pAB205, pAB206 or pAB208. (B) Effect of adding 0 M or 10^{-7} M of C6-HSL on cell growth for different gRNA containing either no mutation ("Original gRNA"), or mutations at 4 bp, 2 bp or directly before the PAM sequence (denoted "End-3", "End-1" and "End-0" respectively). "WT" shows the OD of the wild-type BW25113 strain. "-HSL" and "+HSL" show growth of the cell containing the CRISPRi plasmid when 0 M and 10^{-7} M of C6-HSL is added to the M9 medium without casamino acids. OD measurements were collected in a time-course plate-reader assay. Data points represent three biological replicates, and the curves were fitted to the means of these points using a smoothing spline. Plasmids used in this figure are recorded in Supplementary Data 2 and strains in Supplementary Table 1. Source data are provided as a Source Data file.

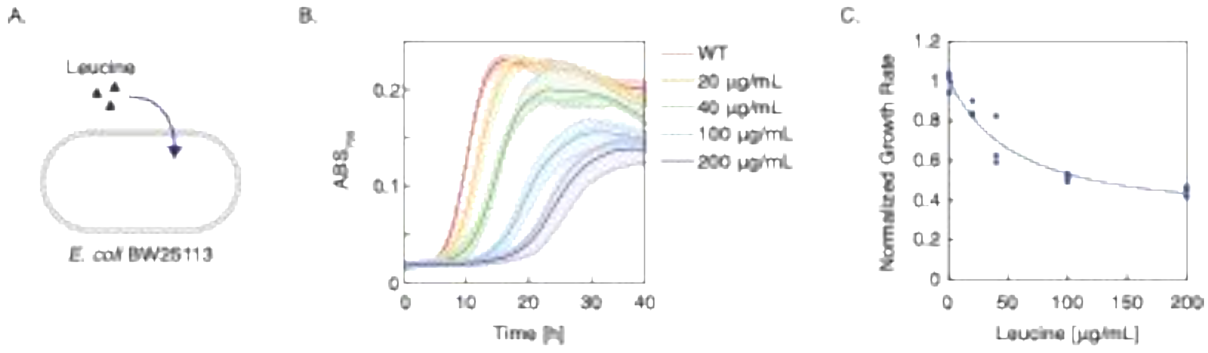

<u>Supplementary Figure 15:</u> **Growth rate modulation via external chemical control.** (A) BW25113 cells were grown in M9 medium without casamino acids and with different concentrations of L-leucine. L-leucine causes toxicity by inhibiting the expression of the ilvGM and ilvBN operons. (B) OD of *E. coli* BW25113 when different concentrations of leucine are added to the media. (C) Maximum growth rate of *E. coli* BW25113 when different concentrations of L-leucine are added to the media and normalized to the 0 μg/mL of L-leucine condition. OD measurements were collected in a time-course plate-reader assay. Data points represent three biological replicates, and the curves were fitted to the means of these points using a smoothing spline. Source data are provided as a Source Data file.

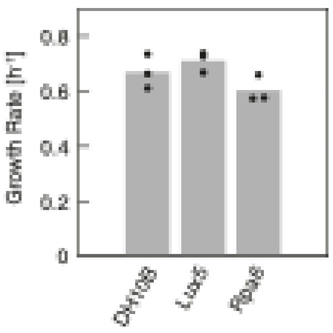

Supplementary Figure 16: **Growth of quorum-sensing-producer strains.** Maximum growth rate of the strongest C6-HSL-producing *E. coli* DH10B strain (Lux5) and the strongest pC-HSL-producing *E. coli* DH10B strain (Rpa6) compared to the growth rate of the wild-type *E. coli* DH10B strain (Supplementary Table 1). OD measurements were collected in time-course plate-reader assay. The data points represent 3 biological replicates, and the bars represent the means of these points. Strains used in this figure are recorded in Supplementary Table 1. Source data are provided as a Source Data file.

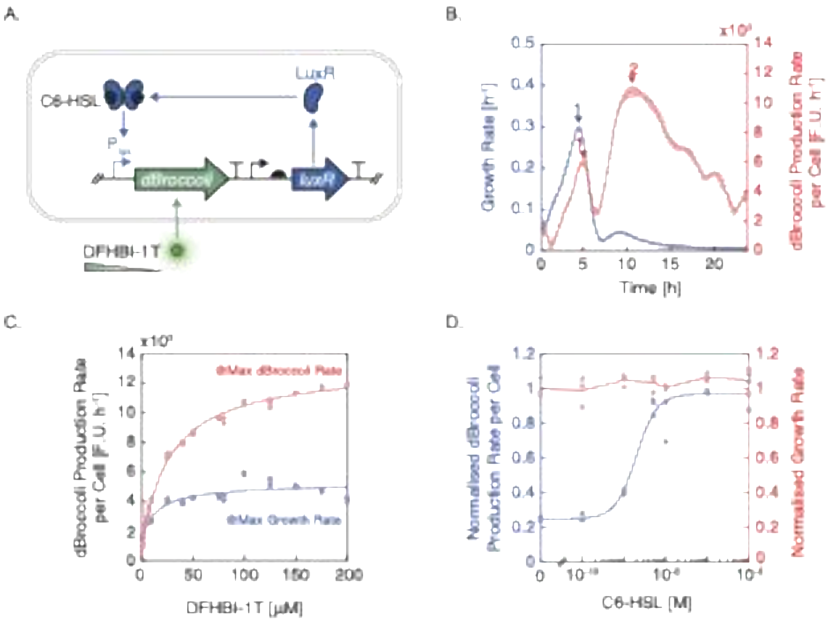

<u>Supplementary Figure 17:</u> Effect of DFHBI-1T dye concentration on dBroccoli signal strength and host growth rate. (A) Different concentrations of DFHBI-1T dye are tested to study the impact of DFHBI-1T concentration on dBroccoli signal strength. dBroccoli is expressed under the LLL quorum sensing system, with 10^{-7} M of C6-HSL in DH10B carrying plasmid pAB420. (B) dBroccoli production rate per cell has two maxima: one coinciding with the maximum growth rate of the cells during exponential phase, and the other when the culture is in stationary phase. (C) Effect of DFHBI-1T dye concentration on dBroccoli production rate per cell at both dBroccoli production maxima. Cells are induced with 10^{-7} M of C6-HSL. (D) Effect of dBroccoli expression on growth rate at the time of maximum growth rate. dBroccoli production rate per cell is normalized to the maximum C6-HSL inducer concentration condition (10^{-7} M), while the growth rate is normalized to the no C6-HSL inducer condition (0 M). Cells are mixed with 100 µM of DFHBI-1T dye. dBroccoli fluorescence and OD were monitored in a time-course plate-reader assay. Data points represent three biological replicates, and the curves were fitted to the means of these points using MATLAB four-parameter nonlinear regression fit or a smoothing spline. Plasmids used in this figure are recorded in Supplementary Data 2 and strains in Supplementary Table 1. Source data are provided as a Source Data file.

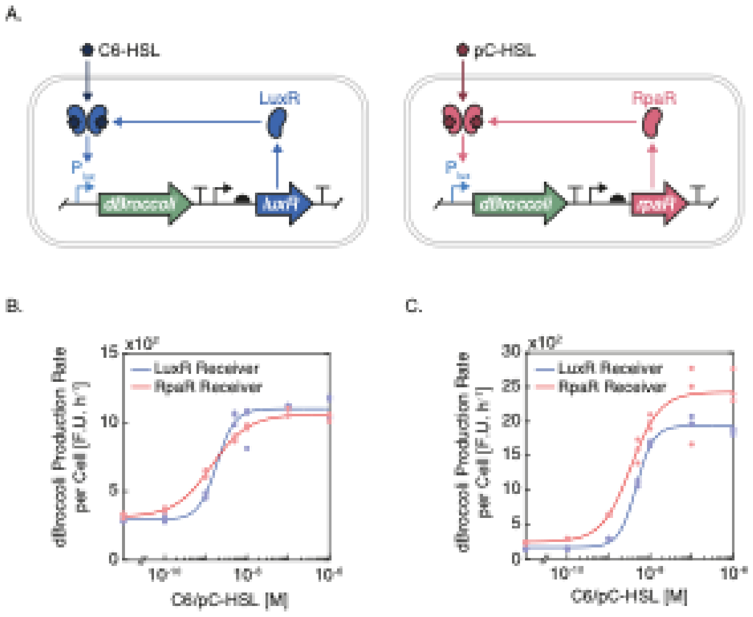

<u>Supplementary Figure 18:</u> **Response of the lux and rpa systems to HSL gradients.** (A) The LuxR Receiver circuit is composed of the LLL driving the expression of dBroccoli in DH10B carrying plasmid pAB420. The RpaR Receiver is composed of the LRR system driving the expression of dBroccoli in DH10B carrying plasmid pAB421. (B) dBroccoli production rate per cell at the time of maximum growth rate, for different concentrations of C6-HSL and pC-HSL added to the LuxR Receiver and the RpaR Receiver respectively. (C) dBroccoli production rate per cell at the time of maximum dBroccoli production, for different concentrations of C6-HSL and pC-HSL added to the LuxR Receiver and the RpaR Receiver respectively. dBroccoli fluorescence and OD were monitored in a time-course plate-reader assay. Data points represent three biological replicates, and the curves were fitted to the means of these points using MATLAB four-parameter nonlinear regression fit. Plasmids used in this figure are recorded in Supplementary Data 2 and strains in Supplementary Table 1. Source data are provided as a Source Data file.

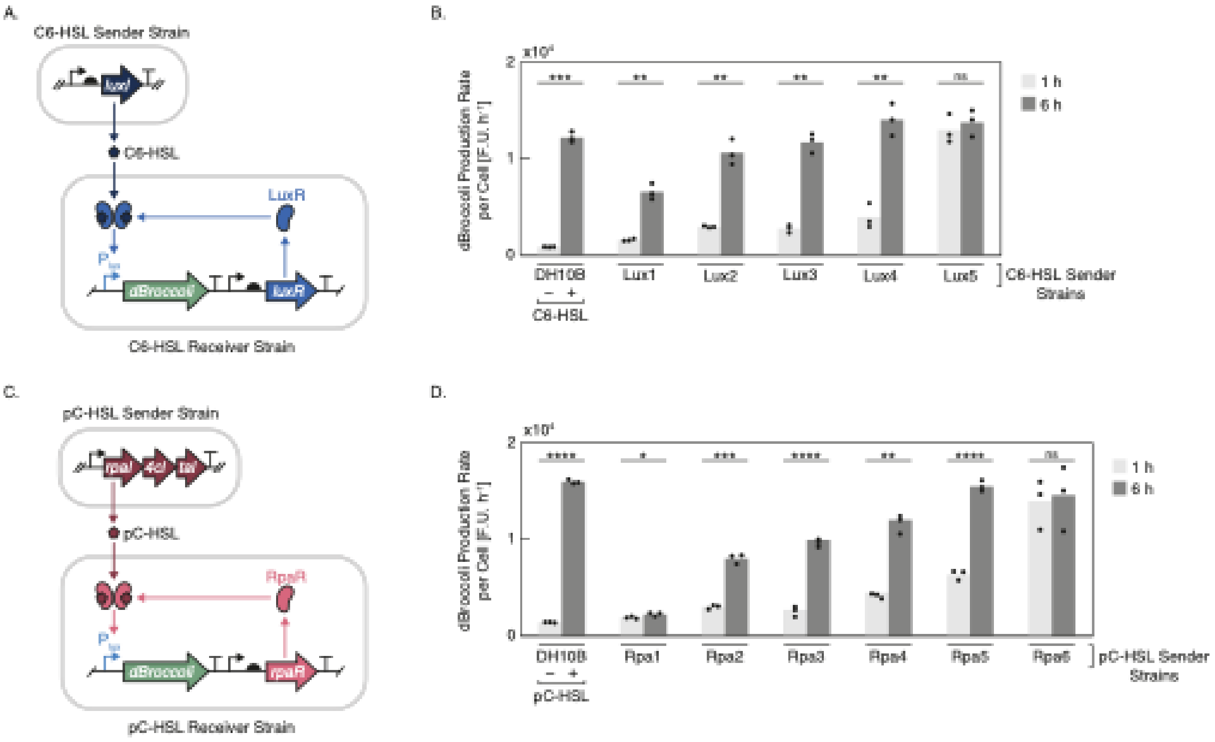

<u>Supplementary Figure 19:</u> Building of C6-HSL and pC-HSL sender/receiver strains. (A) Sender strains producing C6-HSL are incubated for a period of 1 to 6 hours before being centrifuged, their supernatants collected and mixed with the C6-HSL receiver strain. (B) dBroccoli expression per cell of the DH10B C6-HSL receiver strain carrying plasmid pAB420 in response to the C6-HSL produced by the Lux1, Lux2, Lux3, Lux4 and Lux5 C6-HSL sender strains. Control is the C6-HSL receiver strain grown with 0 M of C6-HSL ("−") or 10^{-7} M of C6-HSL ("+"). (C) Sender strains producing pC-HSL are incubated for a period of 1 to 6 hours before being centrifuged, their supernatants collected and mixed with the pC-HSL receiver strain. (D) dBroccoli expression per cell of the DH10B pC-HSL receiver strain carrying the pAB421 plasmid in response to the pC-HSL produced by the Rpa1, Rpa2, Rpa3, Rpa4, Rpa5 and Rpa6 pC-HSL sender strains. Control is the pC-HSL receiver strain grown with 0 M of pC-HSL ("−") or 10^{-7} M of pC-HSL ("+"). dBroccoli fluorescence and OD were measured in a time-course plate-reader assay. The data points represent 3 biological replicates, and the bars represent the means of these points. Statistically significant differences were determined using two-tailed Student's t-test (**** represents $p<0.0001$, *** represents $p<0.001$, ** represents $p<0.01$, * represents $p<0.1$, ns represents not significant). Plasmids used in this figure are recorded in Supplementary Data 2 and strains in Supplementary Table 1. Source data are provided as a Source Data file.

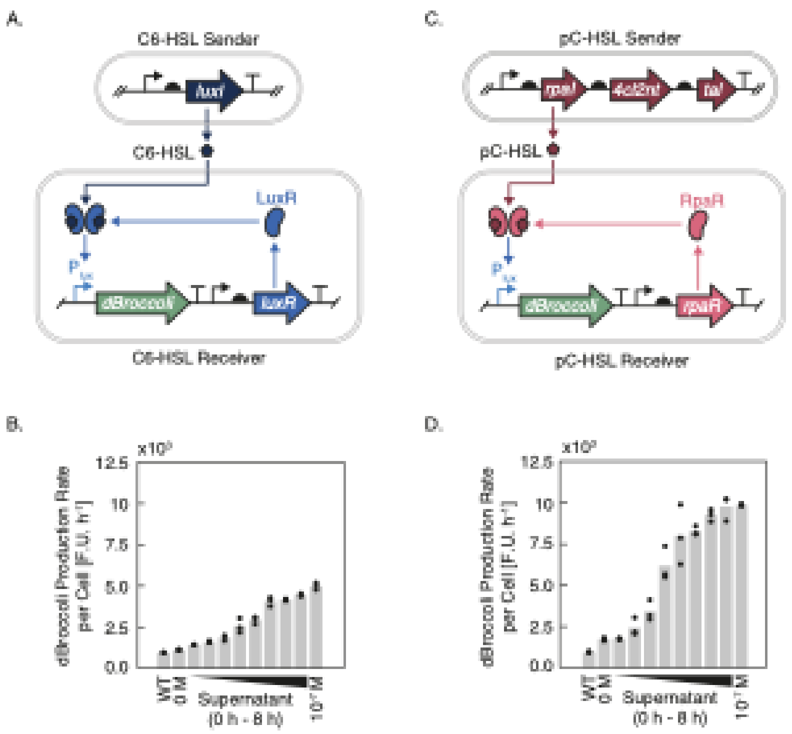

<u>Supplementary Figure 20:</u> Quorum sensing production by the pC-HSL and C6-HSL producer strains. (A) The Lux1 sender strain producing C6-HSL is incubated for a period of 0, 1, 2, 3, 4, 5, 6 or 8 hours before being centrifuged for its supernatant to be collected every hour and mixed with the DH10B C6-HSL receiver strain carrying plasmid pAB420. (B) dBroccoli expression per cell of DH10B WT or the C6-HSL receiver strain grown with 0 M, 10^{-7} M of C6-HSL or the supernatants of Lux1. (C) The Rpa5 sender strain producing pC-HSL is incubated for a period of 0, 1, 2, 3, 4, 5, 6 or 8 hours before being centrifuged for its supernatant to be collected every hour and mixed with the DH10B pC-HSL receiver strain carrying plasmid pAB421. (D) dBroccoli expression per cell of DH10B WT or the pC-HSL receiver strain grown with 0 M, 10^{-7} M of pC-HSL or the supernatants of Rpa5. dBroccoli fluorescence and OD were measured in a time-course plate-reader assay. Plasmids used in this figure are recorded in Supplementary Data 2 and strains in Supplementary Table 1. Source data are provided as a Source Data file.

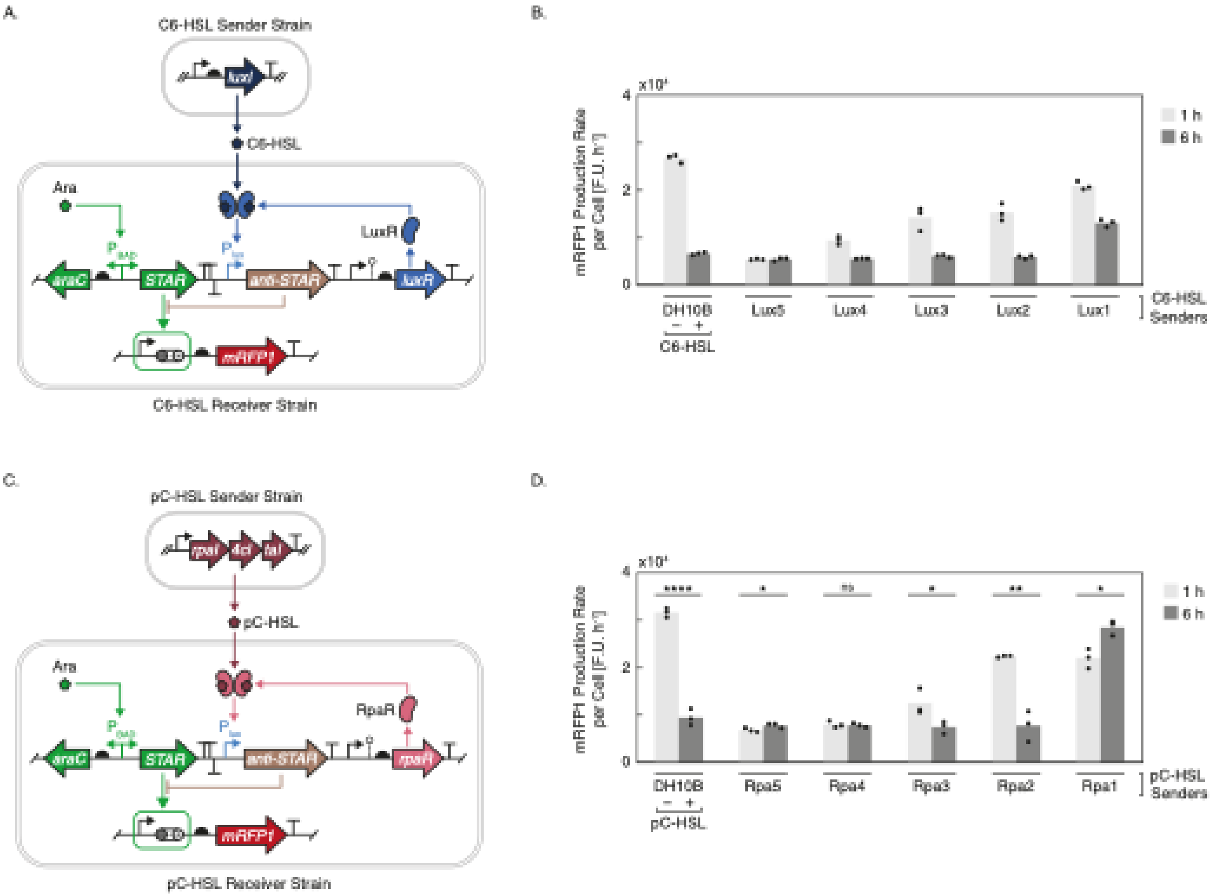

Supplementary Figure 21: **STAR comparator response to C6-HSL and pC-HSL sender strains.** (A) Sender strains producing C6-HSL are incubated for a period of 1 to 6 hours before being centrifuged, their supernatants collected and mixed with the C6-HSL receiver strain. (B) mRFP expression per cell of the DH10B C6-HSL receiver strain carrying plasmids pJBL5939 and pAB300 in response to C6-HSL produced by the Lux1, Lux2, Lux3, Lux4 and Lux5 C6-HSL sender strains. Control is the C6-HSL receiver strain grown with 0 M of C6-HSL (“–”) or 10^{-7} M of C6-HSL (“+”). (C) Sender strains producing pC-HSL are incubated for a period of 1 hour or 6 hours before being centrifuged, their supernatants collected and mixed with the pC-HSL receiver strain. (D) mRFP expression per cell of the DH10B pC-HSL receiver strain carrying plasmids pJBL5939 and pAB401 in response to pC-HSL produced by the Rpa1, Rpa2, Rpa3, Rpa4, and Rpa5 pC-HSL sender strains. Control is the pC-HSL receiver strain grown with 0 M of pC-HSL (“–”) or 10^{-7} M of pC-HSL (“+”). mRFP fluorescence and OD were measured in a time-course plate-reader assay. The data points represent 3 biological replicates, and the bars represent the means of these points. Plasmids used in this figure are recorded in Supplementary Data 2 and strains in Supplementary Table 1. Source data are provided as a Source Data file.

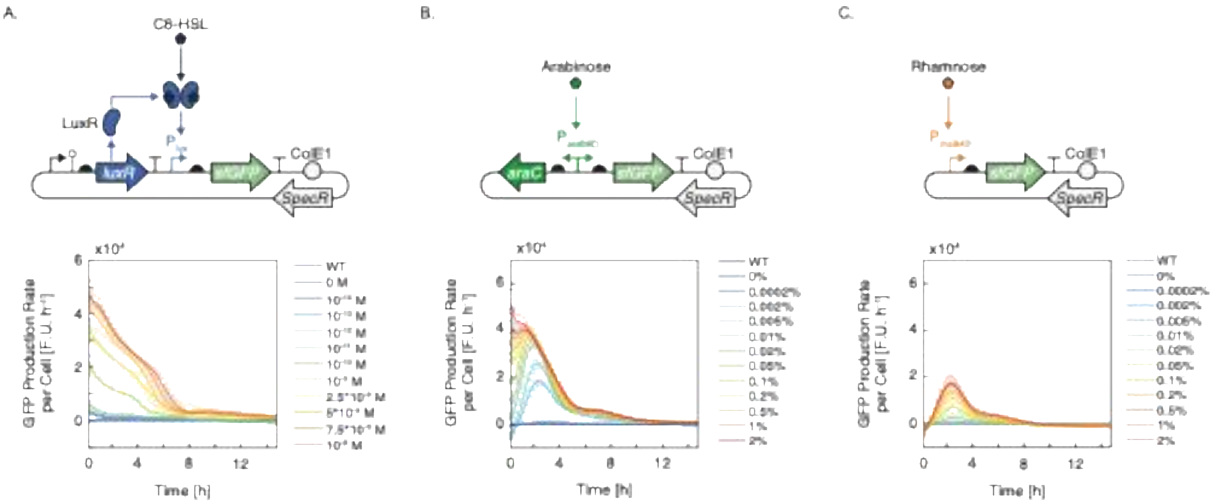

<u>Supplementary Figure 22:</u> **Comparison of the LLL, araBAD and rhaBAD inducible systems.** (A) sfGFP production rate per cell for increasing C6-HSL concentrations. The lux promoter of the LLL quorum sensing system driving expression of sfGFP was externally induced with C6-HSL in BW25113 carrying plasmid pAB252. (B) sfGFP production rate per cell as a function of increasing L-arabinose concentration. The araBAD promoter driving expression of sfGFP was externally induced with L-arabinose in BW25113 carrying pAB409. (C) sfGFP production rate per cell as a function of increasing L-arabinose concentration. The rhaBAD promoter driving expression of sfGFP was externally induced with L-rhamnose in BW25113 carrying pAB410. Fluorescence and OD measurements were collected in a plate-reader. Data points represent three biological replicates, and the curves were fitted to the means of these points using a smoothing spline. Plasmids used in this figure are recorded in Supplementary Data 2 and strains in Supplementary Table 1. Source data are provided as a Source Data file.

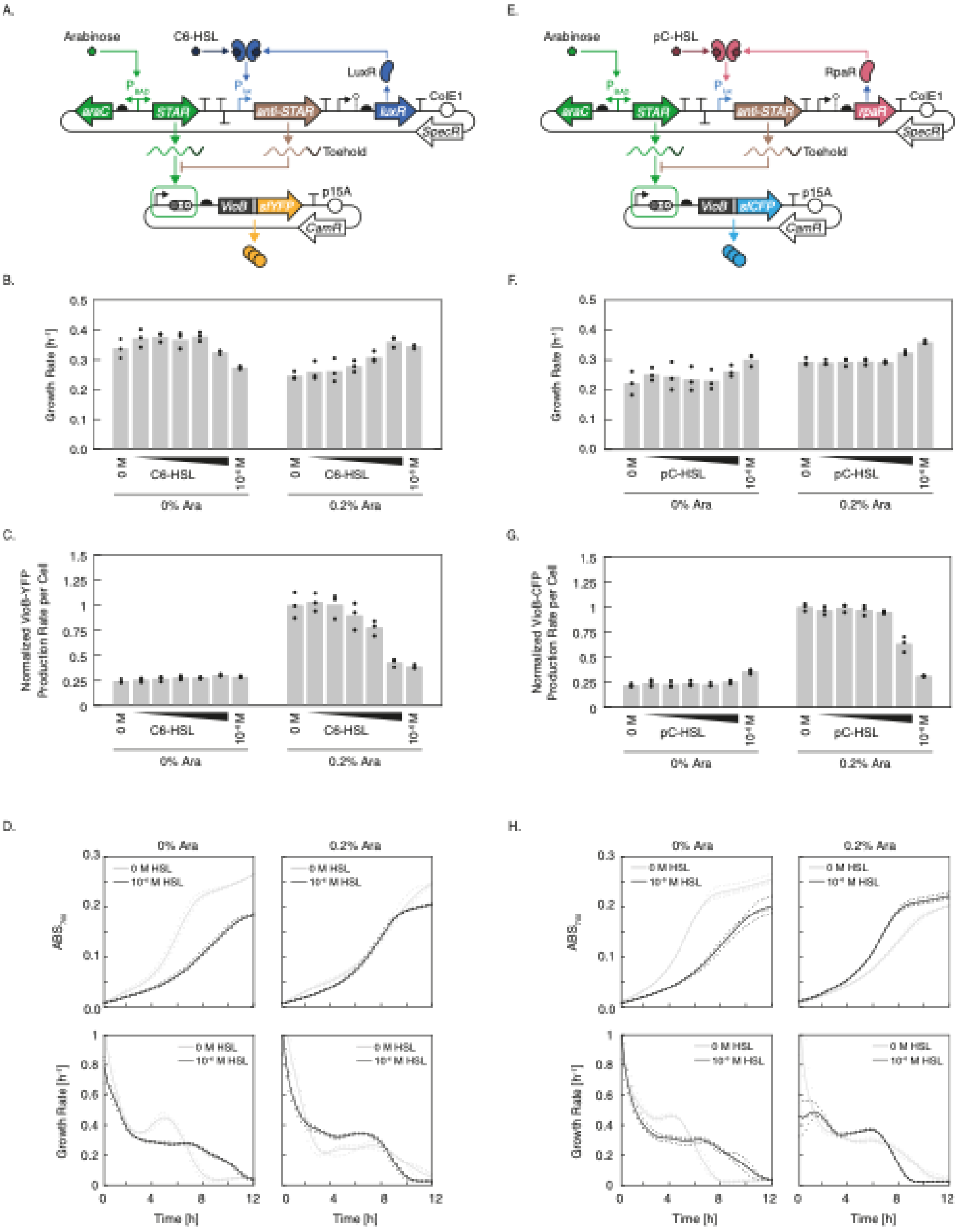

<u>Supplementary Figure 23:</u> **Building coculture strains with different burden levels.** (A) The YFP strain regulates VioB-sfYFP production under the control of the LLL STAR-based comparator with toehold T2 in DH10B carrying plasmids pAB518 and pAB300. VioB-sfYFP expression is activated by L-arabinose and inhibited with increasing concentration of C6-HSL (0, 10^{-10}, 10^{-9}, 5×10^{-9}, 10^{-8}, 10^{-7}, 10^{-6} M). (B) Growth rate at 6 h of the YFP strain. (C) VioB-sfYFP production per cell of the YFP strain at 6 h, normalized to the condition: 0 M C6-HSL, 0.2% L-arabinose. (D) OD and growth rate over 12 hours of

the YFP strain induced with either 0% or 0.2% of L-arabinose to activate VioB-sfYFP expression. (E) The CFP strain regulates VioB-sfCFP expression using the LRR STAR-based comparator with toehold T2 in DH10B carrying pAB519 and pAB401. VioB-sfCFP expression is activated by L-arabinose and inhibited with increasing concentration of pC-HSL (0, 10^{-10}, 10^{-9}, 5×10^{-9}, 10^{-8}, 10^{-7}, 10^{-6} M). (F) Growth rate at 6 h of the CFP strain. (G) VioB-sfCFP production per cell of the CFP strain at 6 h, normalized to the condition: 0 M pC-HSL, 0.2% L-arabinose. (H) OD and growth rate over 12 hours of the CFP strain induced with either 0% or 0.2% of L-arabinose to activate VioB-sfCFP expression. Fluorescence and OD measurements were collected in a plate-reader. The data points represent 3 biological replicates. The bars represent the means of these points. The curves were fitted to the means of these points using a smoothing spline. Plasmids used in this figure are recorded in Supplementary Data 2 and strains in Supplementary Table 1. Source data are provided as a Source Data file.

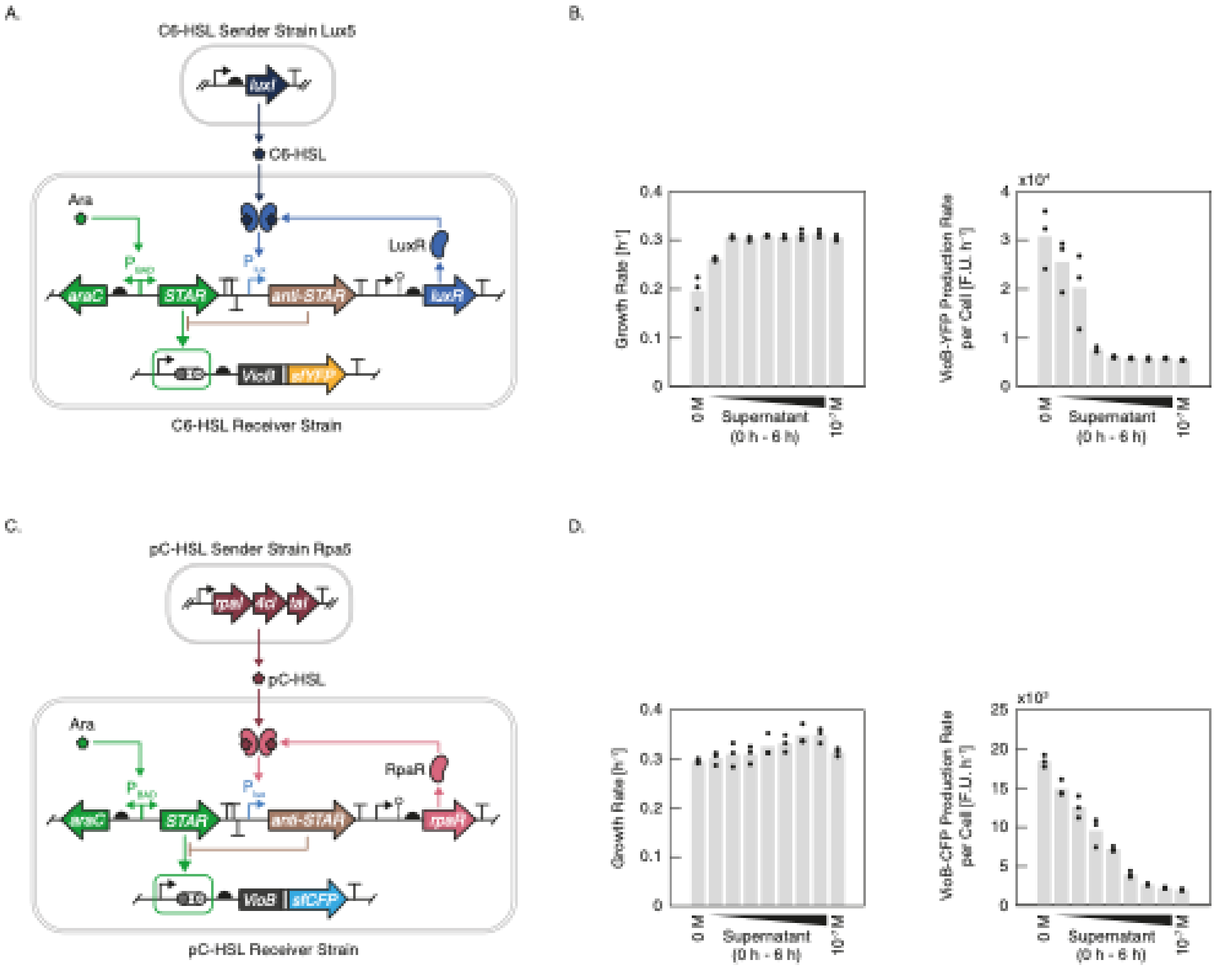

<u>Supplementary Figure 24:</u> Response of the coculture strains to HSL production by a pC-HSL and C6-HSL sender strain. (A) The Lux5 sender strain producing C6-HSL is incubated for a period of 0 to 6 hours. Supernatants collected every hour are then mixed with the DH10B C6-HSL receiver strain carrying plasmids pAB518 and pAB300. (B) Growth rate and VioB-sfYFP expression of DH10B WT or the C6-HSL receiver strain grown with 0 M, 10^{-7} M of C6-HSL or the supernatants of the Lux5 strain at the time of maximum growth rate. (C) The Rpa5 sender strain producing pC-HSL is incubated for a period of 0 to 6 hours. Supernatants collected every hour are then mixed with the DH10B pC-HSL receiver strain carrying plasmid pAB519 and pAB401. (D) Growth rate and VioB-sfCFP expression of DH10B WT or the pC-HSL receiver strain grown with 0 M, 10^{-7} M of pC-HSL or the supernatants of the Rpa5 strain at the time of maximum growth rate. Fluorescence and OD were measured in a time-course plate-reader assay. The data points represent 3 biological replicates, and the bars represent the means of these points. Plasmids used in this figure are recorded in Supplementary Data 2 and strains in Supplementary Table 1. Source data are provided as a Source Data file.

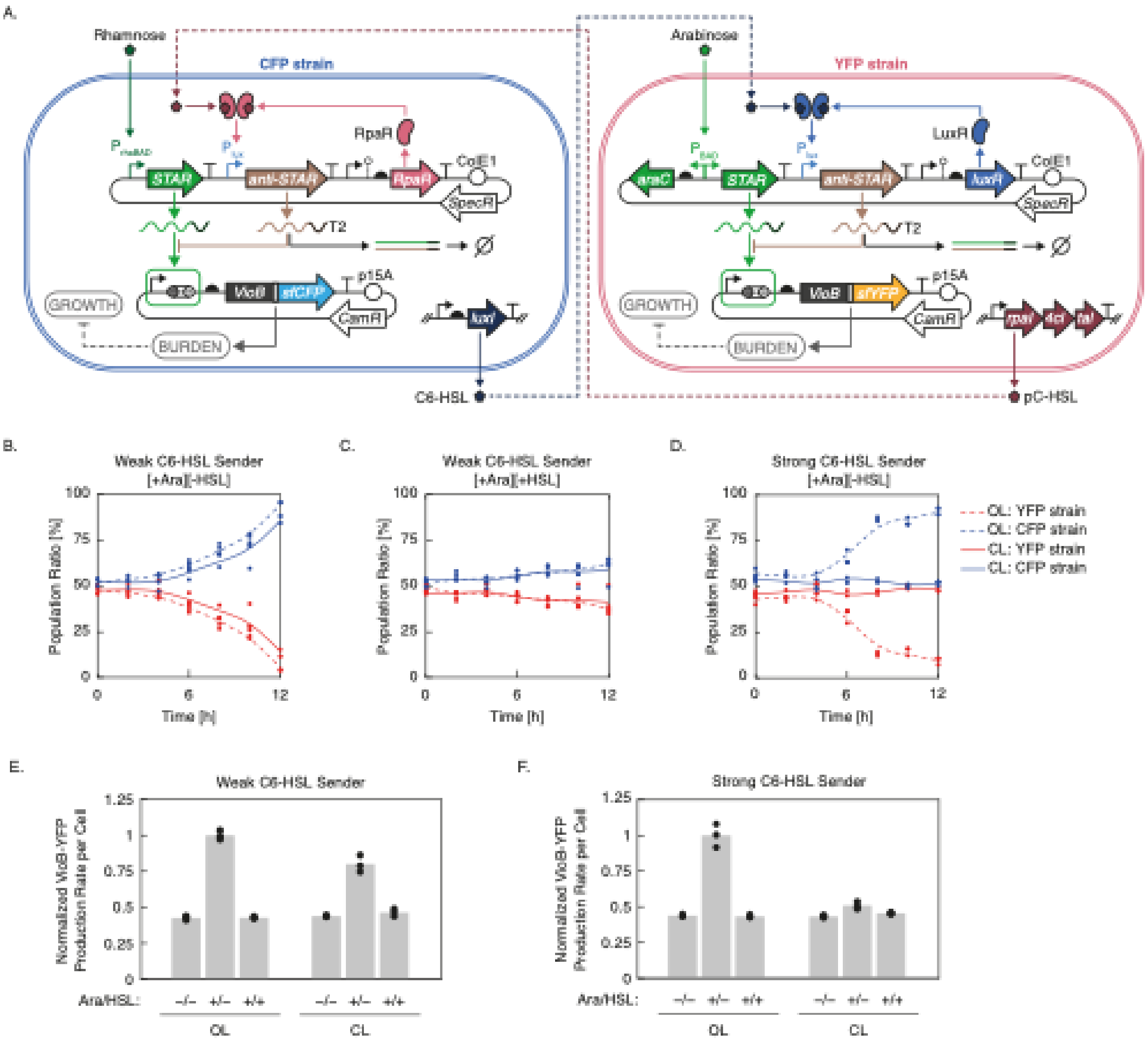

<u>**Supplementary Figure 25:**</u> **Effect of C6-HSL production on coculture composition.** (A) The Lux4 and Lux5 C6-HSL sender strains, or CFP strains, are tested to study the effect of C6-HSL signal strength on coculture composition of the closed-loop circuit ("CL"). The YFP strain produces VioB-sfYFP in the presence of L-arabinose and VioB-sfYFP production is down-regulated by C6-HSL. The YFP strain is Rpa5 carrying plasmids pAB518 and pAB300. The CFP strain produces VioB-sfCFP in the presence of L-rhamnose and VioB-sfCFP production is down-regulated by pC-HSL. The CFP strain is either Lux4 or Lux5 carrying plasmids pAB519 and pAB537. In the open-loop circuit ("OL"), to break communication between the strains, wild-type DH10B strains are used such that neither strain produces quorum sensing molecules. (B) Change in population ratio when the coculture is made up of the weak C6-HSL Lux4 sender CFP strain and the Rpa5 YFP strain. Coculture is induced with 0.2% of L-arabinose, 0% of L-rhamnose, and 0M of both C6-HSL and pC-HSL. (C) Change in population ratio when the coculture is made up of the weak C6-HSL Lux4 sender CFP strain and the Rpa5 YFP strain, when the coculture is induced with 0.2% of L-arabinose, 0% of L-rhamnose, 10^{-7} M of C6-HSL and 0 M of pC-HSL. (D) Change in population ratio when the coculture is made up of the strong C6-HSL Lux5 sender CFP strain and the Rpa5 YFP strain. The coculture is induced with 0.2% of L-arabinose, 0% L- rhamnose and 0M of both C6-HSL and pC-HSL. (E-F) Normalised VioB-sfYFP production rate per cell for the open-loop and closed-loop cocultures with the Lux4 and Lux5 C6-HSL sender strains. Three inducer conditions were tested

by adding different combinations of the four inducers. (1) "-/-": 0% of L-arabinose, 0% of L-rhamnose, 0 M of pC-HSL, 0 M of C6-HSL. (2) "+/-": 0.2% of L-arabinose, 0% of L-rhamnose, 0 M of pC-HSL, 0 M of C6-HSL. (3) "+/+": 0.2% of L-arabinose, 0% of L-rhamnose, 0 M of pC-HSL, 10^{-7} M of C6-HSL. The population ratio was measured through flow-cytometry. VioB-sfYFP fluorescence and OD were measured in a time-course plate-reader assay. The data points represent 3 biological replicates, and the bars represent the means of these points. Curves were fitted to the means of these points using a smoothing spline. Plasmids used in this figure are recorded in Supplementary Data 2 and strains in Supplementary Table 1. Source data are provided as a Source Data file.

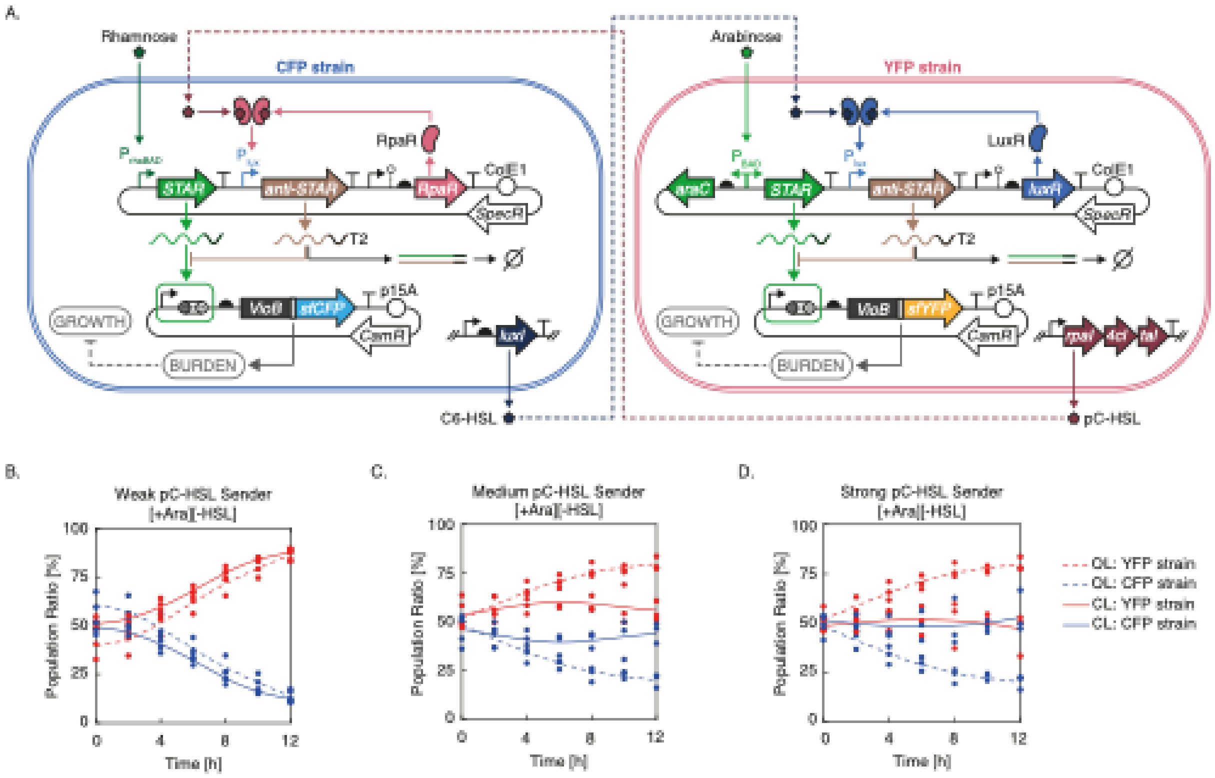

Supplementary Figure 26: **Effect of pC-HSL production on coculture composition.** (A) Rpa2,4,5 pC-HSL sender strains, or CFP strains, are tested to study the effect of pC-HSL signal strength on coculture composition of the closed-loop circuit ("CL"). The YFP strain produces VioB-sfYFP when L-arabinose is present and VioB-sfYFP production is down-regulated by pC-HSL. The YFP strain is Lux5 carrying plasmids pAB518 and pAB401. The CFP strain produces VioB-sfCFP in the presence of L-rhamnose, and VioB-sfCFP production is down-regulated by C6-HSL. The CFP strain is either Rpa2, Rpa4 or Rpa5 carrying plasmids pAB519 and pAB399. In the open-loop circuit ("OL"), to break communication between the strains, wild-type DH10B strains are used such that neither strain produces quorum sensing molecules. (B) Change in population ratio when the coculture is made up of the weak pC-HSL Rpa2 sender CFP strain and the Lux5 YFP strain. Coculture is induced with 0.2% of L-arabinose, 0% of L-rhamnose, and 0M of both C6-HSL and pC-HSL. (C) Change in population ratio when the coculture is made up of the medium-strength pC-HSL Rpa4 sender CFP strain and the Lux5 YFP strain. Coculture is induced with 0.2% of L-arabinose, 0% of L-rhamnose, and 0M of both C6-HSL and pC-HSL. (D) Change in population ratio when the coculture is made up of the strong pC-HSL Rpa5 sender CFP strain and the Lux5 YFP strain. Coculture is induced with 0.2% of L-arabinose, 0% of L-rhamnose, and 0M of both C6-HSL and pC-HSL. The population ratio was measured through flow-cytometry. Data points represent three biological replicates, and the curves were fitted to the means of these points using a smoothing spline. Plasmids used in this figure are recorded in Supplementary Data 2 and strains in Supplementary Table 1. Source data are provided as a Source Data file.

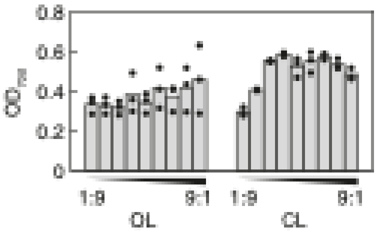

<u>Supplementary Figure 27:</u> **Final OD of Figure 4F.** Final OD taken after 24 hours of growing the cocultures from Figure 4F. OD was measured in a time-course plate-reader assay. The data points represent 3 biological replicates, and the bars represent the means of these points. Plasmids used in this figure are recorded in Supplementary Data 2 and strains in Supplementary Table 1. Source data are provided as a Source Data file.

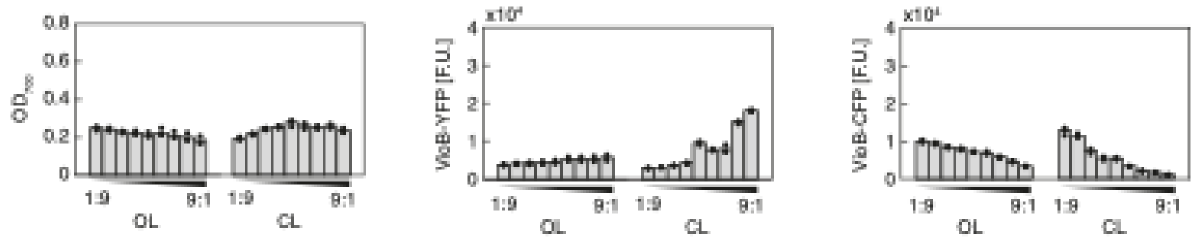

Supplementary Figure 28: **OD, VioB-YFP and VioB-CFP at 12 h of Figure 4F.** OD, VioB-sfYFP yield and VioB-sfCFP yield taken after 12 hours of growing the cocultures from Figure 4F. OD and fluorescence were measured in a time-course plate-reader assay. The data points represent 3 biological replicates, and the bars represent the means of these points. Plasmids used in this figure are recorded in Supplementary Data 2 and strains in Supplementary Table 1. Source data are provided as a Source Data file.

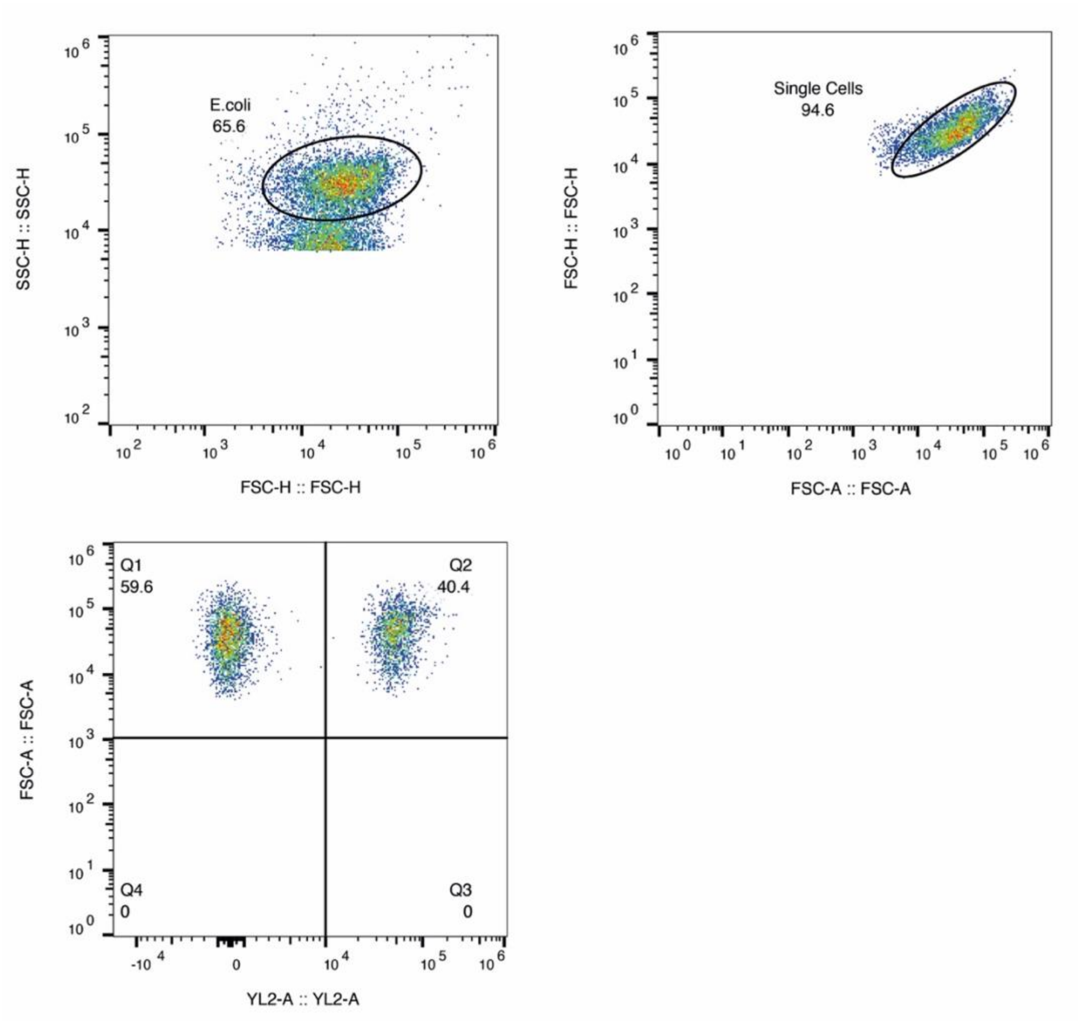

<u>Supplementary Figure 29:</u> **Gating strategy for flow cytometry.** 10,000 cells were collected for each sample and data were analysed using FlowJo. The *E. coli* population was gated using FSC-H and SSC-H to remove background noise. *E. coli* cells were then gated for singlets using FSC-H and FSC-A. To determine coculture composition, one *E. coli* strain was genomically integrated with a constitutive mScarlet-I cassette. The red-fluorescent and non-fluorescent populations were determined by gating using YL2-A and FSC-A.

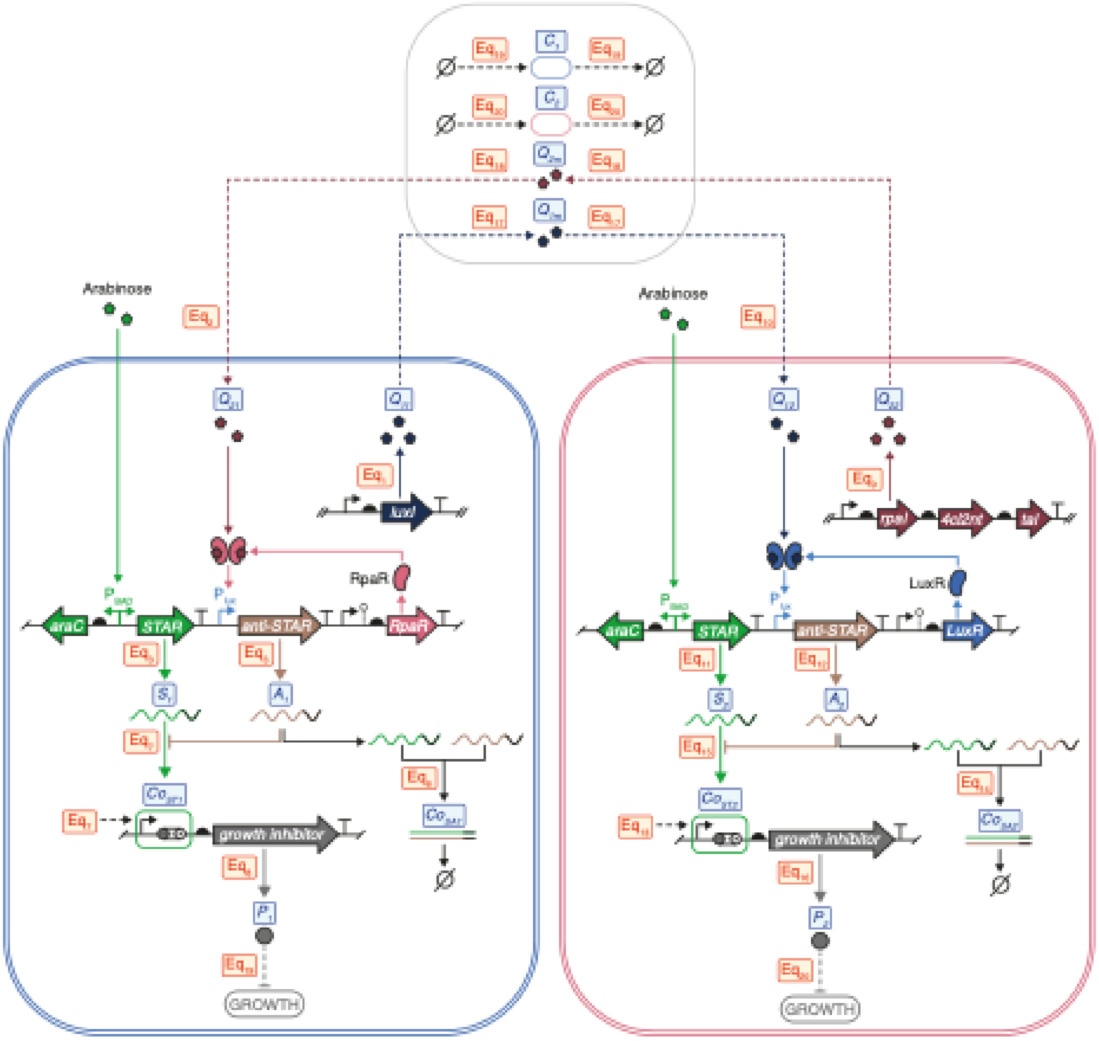

Supplementary Figure 30: **Graphical representation of the MATLAB SimBiology model.** The equations of the model are presented in Supplementary Note 1.

Supplementary Table 1: **Strains**

Name	Genotype	Antibiotic	Source/Parent
DH10B	K-12 strain, F– mcrA Δ(mrr-hsdRMS-mcrBC) ϕ80lacZΔM15 ΔlacX74 recA1 endA1 araD139 Δ(ara,leu)7697 galU galK λ–rpsL(StrR) nupG	Streptomycin	Invitrogen
BW25113	K-12 strain, acI+rrnBT14 ΔlacZWJ16 hsdR514 ΔaraBADAH33 ΔrhaBADLD78 rph-1 Δ(araB–D)567 Δ(rhaD–B)568 ΔlacZ4787(::rrnB-3) hsdR514 rph-1	None	Keio Collection
BW25113(rpsL150)	rpsL150 (strR)	Streptomycin	BW25113
PIR2 One Shot	F- Δlac169 rpoS(Am) robA1 creC510 hsdR514 endA recA1 uidA(ΔMluI)::pir	None	Invitrogen
TransforMax EC100D pir+	F- mcrA Δ(mrr-hsdRMS-mcrBC) ϕ80dlacZΔM15 ΔlacX74 recA1 endA1 araD139 Δ(ara, leu)7697 galU galK λ- rpsL nupG pir+(DHRF)	None	Lucigen
JW5807	ΔleuB	None	BW25113
DH10B-GFP	J23100–sfGFP–BBa_1002 at attλ	Kanamycin	DH10B
DH10B-mScarlet	J23100–mScarlet-I–BBa_1002 at attλ	Kanamycin	DH10B
BW25113-GFP	J23100–sfGFP–BBa_1002 at attλ	Kanamycin	BW25113
BW25113-mScarlet	J23100–mScarlet-I–BBa_1002 at attλ	Kanamycin	BW25113
Lux1	J23100–RBSc33–luxI–L3S2P55 at attλ (CRIM plasmid pAB386)	Kanamycin	DH10B
Lux2	J23115–B0030–luxI–L3S2P55 at attλ (CRIM plasmid pAB566)	Kanamycin	DH10B
Lux3	J23105–B0030–luxI–L3S2P55 at attλ (CRIM plasmid pAB564)	Kanamycin	DH10B
Lux4	J23106–B0030–luxI–L3S2P55 at attλ (CRIM plasmid pAB554)	Kanamycin	DH10B
Lux5	J23100–B0030–luxI–L3S2P55 at attλ plasmid (CRIM plasmid pAB553)	Kanamycin	DH10B
Rpa1	J23109–B0030–rpaI–B0030–4CL2nt–B0032– TAL–L3S2P55 at attλ (CRIM plasmid pAB572)	Kanamycin	DH10B
Rpa2	J23109–B0030–rpaI–B0030–4CL2nt–B0030– TAL–L3S2P55 at attλ (CRIM plasmid pAB496)	Kanamycin	DH10B
Rpa3	J23106–B0030–rpaI–B0030–4CL2nt–B0030– TAL–L3S2P55 at attλ (CRIM plasmid pAB495)	Kanamycin	DH10B
Rpa4	J23105–B0030–rpaI–B0030–4CL2nt–B0030– TAL–L3S2P55 at attλ (CRIM plasmid pAB494)	Kanamycin	DH10B
Rpa5	J23114–B0030–rpaI–B0030–4CL2nt–B0030– TAL–L3S2P55 at attλ (CRIM plasmid pAB493)	Kanamycin	DH10B
Rpa6	J23100–B0030–rpaI–B0030–4CL2nt–B0032– TAL–L3S2P55 at attλ (CRIM plasmid pAB482)	Kanamycin	DH10B

Supplementary Table 2: Model variables

Variable	Description	Units
Q_{11}	Concentration of Quorum sensing molecule 1 in cell 1	nM
Q_{21}	Concentration of Quorum sensing molecule 2 in cell 1	nM
S_1	Concentration of STAR in cell 1	tr
A_1	Concentration of anti-STAR in cell 1	tr
T_1	Concentration of the Target RNA in cell 1	tr
Co_{SA1}	Concentration of STAR-anti-STAR complex in cell 1	co
Co_{ST1}	Concentration of STAR-Target complex in cell 1	co
P_1	Concentration of Target Protein in cell 1	nM
Q_{22}	Concentration of Quorum sensing molecule 2 in cell 2	nM
Q_{12}	Concentration of Quorum sensing molecule 1 in cell 2	nM
S_2	Concentration of STAR in cell 2	tr
A_2	Concentration of anti-STAR in cell 2	tr
T_2	Concentration of the Target RNA in cell 2	tr
Co_{SA2}	Concentration of STAR-anti-STAR complex in cell 2	co
Co_{ST2}	Concentration of STAR-Target complex in cell 2	co
P_2	Concentration of Target Protein in cell 2	nM
Q_{1m}	Concentration of Quorum sensing molecule 1 in the culture medium	nM
Q_{2m}	Concentration of Quorum sensing molecule 2 in the culture medium	nM
C_1	Concentration of cell 1 in the culture medium	CFU μm^{-3}
C_2	Concentration of cell 2 in the culture medium	CFU μm^{-3}

Supplementary Table 3: Model parameters

Parameter	Description	Value	Units	Source
ρ_o	Production rate of the quorum sensing molecules	595	nM μm^3 h^{-1}	[3]
η	Diffusion rate of the quorum sensing molecules	120	μm^3 h^{-1}	[3]
D	Degradation rate due to dilution by cell division	2	h^{-1}	[3]
y	Degradation rate for the RNA species	13.86	h^{-1}	-
y_P	Degradation rate for the target protein	0	h^{-1}	-
a_{So}	Production rate of STAR	6000	tr h^{-1}	* [4]
a_{Ao}	Constant term for the production rate of anti-STAR	55.368	tr h^{-1}	* [4]
a_A	Coefficient for the quorum sensing dependent term of the production rate of anti-STAR	50000	tr h^{-1}	* [4]
a_{To}	Production rate of the Target RNA	18000	tr h^{-1}	* [4]
θ	Activation coefficient	20	nM	[3]
$k_{Co_{SA}+}$	Complex binding rate for C_A complex	15	co tr^{-1} h^{-1}	-
$k_{Co_{ST}+}$	Complex binding rate for C_T complex	1.54	co tr^{-1} h^{-1}	[3]
k_{Co-}	Spontaneous unbinding rate of CA and CT complexes	0.4032	co^{-1} tr h^{-1}	[3]
k_P	Production rate of the Target protein	2.8448	co^{-1} nM h^{-1}	[6]
k_{C1}	Growth rate of cell 1	0.5	h^{-1}	-
k_{C2}	Growth rate of cell 2	0.5	h^{-1}	-
d_{B1}	Burden produced by target protein P in cell 1	0.001	h nM^{-1}	-
d_{B2}	Burden produced by target protein P in cell 2	0.0034	h nM^{-1}	-
D_C	Death rate for cell 1 and cell 2	0	h^{-1}	-
V	Volume of culture medium	2.0×10^{11}	μm^3	-
C_{max}	Maximum supported cell concentration for the medium	0.145	CFU μM^{-3}	[3]

* *values adjusted from Gorochowski et al. 2020 to compensate for different copy numbers of the plasmids used for the expression of STAR, anti-STAR and the Target RNA.*

Supplementary Note 1: **Modelling and simulations**

The mathematical model developed to represent the system is described by the following set of ordinary differential equations. The equations can be divided into three sets based on the three distinct compartments, namely, within cell 1, within cell 2 and in the culture media. The variables are described in Supplementary Table 2 and the parameters are described in Supplementary Table 3. A graphical representation of the model is presented in Supplementary Figure 30.

In cell 1:

$$\frac{dQ_{11}}{dt} = \rho_o Q_{11} - \frac{\eta(VC_1 Q_{11} - Q_{1m})}{VC_1} - DQ_{11} \quad \text{(Eq}_1\text{)}$$

$$\frac{dQ_{21}}{dt} = \frac{\eta(Q_{2m} - VC_1 Q_{21})}{VC_1} - DQ_{21} \quad \text{(Eq}_2\text{)}$$

$$\frac{dS_1}{dt} = a_{So} + k_{C-}Co_{ST1} + k_{C-}Co_{SA1} - k_{Co_{ST}+}S_1T_1 - k_{Co_{SA}+}S_1A_1 - (y + D)S_1 \quad (3)$$

$$\frac{dA_1}{dt} = a_{Ao} + \frac{a_A Q_{21}^2}{Q_{21}^2 + \theta^2} + k_{Co-}Co_{SA1} - k_{Co_{SA}+}S_1A_1 - (y + D)A_1 \quad (4)$$

$$\frac{dT_1}{dt} = a_{To} + k_{Co-}Co_{T1} - k_{Co_{ST}+}S_1T_1 - (y + D)T_1 \quad (5)$$

$$\frac{dCA_1}{dt} = k_{Co_{SA}+}S_1A_1 - k_{Co-}Co_{SA1} - (y + D)Co_{SA1} \quad (6)$$

$$\frac{dCT_1}{dt} = k_{Co_{ST}+}S_1T_1 - k_{Co-}Co_{ST1} - k_P Co_{ST1} - (y + D)Co_{ST1} \quad (7)$$

$$\frac{dP_1}{dt} = k_P Co_{ST1} - (y_P + D)Co_{ST1} \quad (8)$$

In cell 2:

$$\frac{dQ_{22}}{dt} = \rho_o Q_{22} - \frac{\eta(VC_2 Q_{22} - Q_{2m})}{VC_2} - DQ_{22} \quad (9)$$

$$\frac{dQ_{12}}{dt} = \frac{\eta(Q_{1m} - VC_2 Q_{12})}{VC_2} - DQ_{12} \quad (10)$$

$$\frac{dS_2}{dt} = a_{So} + k_{Co-}Co_{ST2} + k_{Co-}Co_{SA2} - k_{Co_{ST}+}S_2T_2 - k_{Co_{SA}+}S_2A_2 - (y + D)S_2 \quad (11)$$

$$\frac{dA_2}{dt} = a_{Ao} + \frac{a_A Q_{12}^2}{Q_{12}^2 + \theta^2} + k_{Co-}Co_{SA2} - k_{Co_{SA}+}S_2A_2 - (y + D)A_2 \quad (12)$$

$$\frac{dT_2}{dt} = a_{To} + k_{Co-}Co_{ST2} - k_{Co_{ST}+}S_2T_2 - (y + D)T_2 \quad (13)$$

$$\frac{dCA_2}{dt} = k_{Co_{SA}+}S_2A_2 - k_{Co-}Co_{SA2} - (y + D)Co_{SA2} \quad (14)$$

$$\frac{dCT_2}{dt} = k_{Co_{ST}+}S_2T_2 - k_{Co-}Co_{ST2} - k_PCo_{ST2} - (y + D)Co_{ST2} \quad (15)$$

$$\frac{dP_2}{dt} = k_PCo_{ST2} - (y_P + D)Co_{ST2} \quad (16)$$

In the culture media:

$$\frac{dQ_{1m}}{dt} = \eta(VC_1Q_{11} + VC_2Q_{12} - 2Q_{1m}) - DQ_{1m} \quad (17)$$

$$\frac{dQ_{2m}}{dt} = \eta(VC_2Q_{22} + VC_1Q_{21} - 2Q_{2m}) - DQ_{2m} \quad (18)$$

$$\frac{dC_1}{dt} = \frac{k_{C1}C_1\left(1-\frac{C_1+C_2}{C_{max}}\right)}{1+d_{B1}k_PP_1} - D_CC_1 \quad (19)$$

$$\frac{dC_2}{dt} = \frac{k_{C2}C_2\left(1-\frac{C_1+C_2}{C_{max}}\right)}{1+d_{B2}k_PP_2} - D_CC_2 \quad (20)$$

Equations (1), (2), (9) and (10) describe the production and diffusion of the quorum sensing molecules within the cells. Equations (3) and (11) describe the STAR production within the cells. Due to the difference in the hybridization energies of the STAR-anti-Star complex and the STAR-Target RNA complex the rates of binding are taken to be different, while the rate of unbinding of the complex is assumed to be the same for simplicity. Equations (4) and (12) describe the production of anti-STAR within the cells dependent on the quorum sensing molecules. The Hill coefficient for the activation of anti-STAR production by the quorum sensing molecule is assumed to be 2. Equations (5) and (13) describe the production of the Target RNA within the cells. Equations (6), (7), (14) and (15) describe the STAR-anti-STAR and STAR-Target RNA complex formation. Equations (8) and (16) describe the production of the burdensome target protein within the cell. Equations (17) and (18) describe the diffusion of the quorum sensing molecules into and out of the culture media. Equations (19) and (20) describe the cell concentrations within the culture medium.

While the experiments in this study were conducted in batch culture, the model structure is general and can be extended to simulate continuous culture conditions. In that case, the dilution rate D would represent the flow rate of fresh media into the bioreactor rather than the effective dilution from cell

division. Other parameters that would require adjustment include ρ_0, to account for steady-state quorum sensing signal production under constant media flow; η, to reflect altered diffusion dynamics; and degradation rates such as γ and γ_p, which become especially relevant under continuous replenishment. This flexibility allows the model to serve not only as a tool to interpret batch behavior but also as a predictive framework for future implementation of the comparator in continuous bioprocess settings.

The model was constructed using parameter values available from the literature recorded in Supplementary Table 3. Other parameters such as the production rates of STAR (*aS0*), anti-STAR (*aA*) and target RNA (*aT0*) were fitted to better represent biological circuit behaviour. The scales of some of these parameters are varied due to the nature of the equations.

Supplementary References

1. Borkowski, O. *et al.* Cell-free prediction of protein expression costs for growing cells. *Nat. Commun.* **9**, (2018).
2. Ceroni, F. *et al.* Burden-driven feedback control of gene expression. *Nat. Methods* **15**, (2018).
3. Fusco, V., Salzano, D., Fiore, D. & di Bernardo, M. Embedded control of cell growth using tunable genetic systems. *International Journal of Robust and Nonlinear Control* **33**, 4893–4907 (2023).
4. Bartoli, V., Meaker, G. A., di Bernardo, M. & Gorochowski, T. E. Tunable genetic devices through simultaneous control of transcription and translation. *Nature Communications 2020 11:1* **11**, 2095- (2020).
5. You, L., Cox, R. S., Weiss, R. & Arnold, F. H. Programmed population control by cell-cell communication and regulated killing. *Nature* **428**, 868–871 (2004).
