# Supplementary material for: Burden-aware feedback control of microbial consortia
Source: Nat Commun. 2026 May 6;17:6100. doi: 10.1038/s41467-026-72389-6 (PMC13357841; doi:10.1038/s41467-026-72389-6)
Supplement: Supplementary file 2 — Description of Additional Supplementary Files [file 41467_2026_72389_MOESM2_ESM.pdf]

Description of Additional Supplementary Files for:

Burden-aware feedback control of microbial consortia

Alice Boo, Harman Mehta, Rodrigo Ledesma-Amaro, Guy-Bart Stan

Supplementary Data 1 “genetic-parts.doc”.

Bacterial genetic parts are available in the Supplementary File “genetic-parts.pdf”.

Supplementary Data 2 “plasmids.xlsx”.

Plasmids used in each Main and Supplementary Figure are recorded in the Supplementary File “plasmids.pdf”.
