# Supplementary material for: Burden-aware feedback control of microbial consortia
Source: Nat Commun. 2026 May 6;17:6100. doi: 10.1038/s41467-026-72389-6 (PMC13357841; doi:10.1038/s41467-026-72389-6)
Supplement: Supplementary file 3 — Supplementary Data 1 [file 41467_2026_72389_MOESM3_ESM.pdf]

Supplementary Data 1: Genetic parts

Part	Type	DNA Sequence (5’ to 3’)	Source
J23119	Promoter	TTGACAGCTAGCTCAGTCCTAGGTATAATGCTAGC	iGEM J23119
J23100	Promoter	TTGACGGCTAGCTCAGTCCTAGGTACAGTGCTAGC	iGEM J23100
J23106	Promoter	TTTACGGCTAGCTCAGTCCTAGGTATAGTGCTAGC	iGEM J23106
J23105	Promoter	TTTACGGCTAGCTCAGTCCTAGGTACTATGCTAGC	iGEM J23105
J23115	Promoter	TTTATAGCTAGCTCAGCCCTTGGTACAATGCTAGC	iGEM J23115
J23114	Promoter	TTTATGGCTAGCTCAGTCCTAGGTACAATGCTAGC	iGEM J23114
J23109	Promoter	TTTACAGCTAGCTCAGTCCTAGGGACTGTGCTAGC	iGEM J23109
P_{RAND14}	Promoter	TTTACGGCTAGCTCAGTCCTAGGTATTGTGCTAGC	4
P_{lux}	Promoter	ACCTGTAGGATCGTACAGGTTTACGCAAGAAAATGGTTTGTTATAGTCGAATAAA	5
P_{luxA}	Promoter	ACCTGTAGGATCGTACAGGTTTACGCAAGAAAATGGTTTGTTATTGTCGAATAAA	5
P_{luxB}	Promoter	ACCTGTAGGATCGTACAGGTTTACGCAAGAAAATGGTTTGTTACAGTCGAATAAA	5
P_{rpa}	Promoter	GCACCTGTCCGATCGGACAGTATTACGCAAGAAAATGGTTTGTTATAGTCGAATAT	6
P_{rhaBAD}	Promoter	CCACAATTCAGCAAATTGTGAACATCATCACGTTCATCTTTCCCTGGTTGCCAATGGCCCATTTTCCTGTCAGT<br>AACGAGAAGGTCGCGTATTCAGGCGCTTTTTAGACTGGTCGTAATGAA	4
AraC-P_{araBAD}	Promoter	TTATGACAACTTGACGGCTACATCATTCACTTTTTCTTCACAACCGGCACGGAACTCGCTCGGGCTGGCCCCG<br>GTGCATTTTTTAAATACCCGCGAGAAATAGAGTTGATCGTCAAAACCAACATTGCGACCGACGGTGGCGATA<br>GGCATCCGGGTGGTGCTCAAAAGCAGCTTCGCCTGGCTGATACGTTGGTCCTCGCGCCAGCTTAAGACGCTA<br>ATCCCTAACTGCTGGCGGAAAAGATGTGACAGACGCGACGGCGACAAGCAAACATGCTGTGCGACGCTGGC<br>GATATCAAAATTGCTGTCTGCCAGGTGATCGCTGATGTACTGACAAGCCTCGCGTACCCGATTATCCATCGGT<br>GGATGGAGCGACTCGTTAATCGCTTCCATGCGCCGCAGTAACAATTGCTCAAGCAGATTTATCGCCAGCAGC<br>TCCGAATAGCGCCCTTCCCCTTGCCCGGCGTTAATGATTTGCCCAAACAGGTCGCTGAAATGCGGCTGGTGC<br>GCTTCATCCGGGCGAAAGAACCCCGTATTGGCAAATATTGACGGCCAGTTAAGCCATTCATGCCAGTAGGCG<br>CGCGGACGAAAGTAAACCCACTGGTGATACCATTCGCGAGCCTCCGGATGACGACCGTAGTGATGAATCTCT<br>CCTGGCGGGAACAGCAAAATATCACCCGGTCGGCAAACAAATTCTCGTCCCTGATTTTTCACCACCCCCTGAC<br>CGCGAATGGTGAGATTGAGAATATAACCTTTCATTCCCAGCGGTCGGTCGATAAAAAAATCGAGATAACCGT<br>TGGCCTCAATCGGCGTTAAACCCGCCACCAGATGGGCATTAAACGAGTATCCCGGCAGCAGGGGATCATTTT<br>GCGCTTCAGCCATACTTTTCATACTCCCGCCATTCAGAGAAGAAACCAATTGTCCATATTGCATCAGACATTG<br>CCGTCACTGCGTCTTTTACTGGCTCTTCTCGCTAACCAAACCGGTAACCCCGCTTATTAAAAGCATTCTGTAAC<br>AAAGCGGGACCAAAGCCATGACAAAAACGCGTAACAAAAGTGTCTATAATCACGGCAGAAAAGTCCACATT<br>GATTATTTGCACGGCGTCACACTTTGCTATGCCATAGCATTTTTATCCATAAGATTAGCGGATCCTACCTGAC<br>GCTTTTTATCGCAACTCTCTACTGTTTCTCCATACCCGTTTTTTTGGGCTAGC	4
B0030	RBS	TCTAGAGTCACACAGGAAACCTACTAG	iGEM B0030
B0031	RBS	TCTAGAGTCACACAGGAAACCTACTAG	iGEM B0031
B0032	RBS	TCTAGAGTCACACAGGAAAGTACTAG	iGEM B0032
B0033	RBS	TCTAGAGTCACACAGGACTACTAG	iGEM B0033
B0034	RBS	TACTAGAGAAAGAGGAGAAATACTAG	iGEM B0034
RBS8B	RBS	AAGAATTCAAAAGCTCTACAGAGGAGAAAGGATAT	4
RBSc33	RBS	CTACGTTTTTTAGAAAAAGGAGGTATGCGAG	7
RBSc44	RBS	ATCGGATTGGATCCAAAGGAGGTTATACCG	7
RBS_{Bujard}	RBS	GAATTCATTAAAGAGGAGAAAGGTACC	8
RBS_{VioB}	RBS	AGGCATCTTTCAACTAAAGTAAGAGGTAATAATT	4
$RBS_{mCherry}$	RBS	AGGCTGCAGCGAAAGCG	4
RBS_{RFP}	RBS	ATGGCGAGTAGCG	8
RBS_{ctrE}	RBS	TAAGGAGGCTCCTA	1
RBS_{ctrB}	RBS	AGAGGATACATATA	1
RBS_{ctrI}	RBS	TCAGGATTTTTGTA	1
RBS_{ctrY}	RBS	CGAGGAGGTAATAA	1
HH	Ribozyme	ATCTGGCTGATGAGTCCGTGAGGACGAAACGAGTAAGCTCGTCCC	9
HDV	Ribozyme	GGCCGGCATGGTCCCAGCCTCCTCGCTGGCGCCGGCTGGGCAACACCTTCGGGTGGCGAATGG GACT	9
RiboJ	Ribozyme	AGCTGTCACCGGATGTGCTTTCCGGTCTGATGAGTCCGTGAGGACGAAACAGCCTCTACAAAT<br>AATTTTGTTTAA	5
L3S3P21	Terminator	CCAATTATTGAAGGCCTCCCTAACGGGGGGCCTTTTTTTGTTTCTGGTCTCCC	10
ECK120010793	Terminator	TACGTAAAAACCCGCTTCGGCGGGTTTTTACTTT	10

L3S2P55	Terminator	CTCGGTACCAAAGACGAACAATAAGACGCTGAAAAGCGTCTTTTTT	10
L3S2P21	Terminator	CTCGGTACCAAATTCCAGAAAAGAGGCCTCCCGAAAGGGGGGCCTTTTTTCGTTTTGGTCC	10
ECK120033737	Terminator	GGAAACACAGAAAAAAGCCCGCACCTGACAGTGCGGGCTTTTTTTTTCGACCAAAGG	10
ECK120029600	Terminator	TTCAGCCAAAAAACTTAAGACCGCCGGTCTTGTCCACTACCTTGCAGTAATGCGGTGGACAGGATCGGCGGT<br>TTTCTTTTCTCTTCTCAA	10
t500	Terminator	CAAAGCCCGCCGAAAGGCGGGCTTTTTTTT	11
B1001	Terminator	AAAAAAAAACCCCGCTTCGGCGGGGTTTTTTTTT	iGEM B1001
B1002	Terminator	CGCAAAAAACCCCGCTTCGGCGGGGTTTTTTCGC	iGEM B1002
B0015	Terminator	CCAGGCATCAAATAAAACGAAAGGCTCAGTCGAAAGACTGGGCCTTTCGTTTTATCTGTTGTTTGTCGGTGA<br>ACGCTCTCTACTAGAGTCACA CTGGCTCACCTTCGGGTGGGCCTTTCTGCGTTTATA	iGEM B0015
tL3	Terminator	AATGGCGATGACGCATCCTCACGATAATATCCGGGTAGGCGCAATCACTTTCGTCTCTACTCCGTTACAAAGC<br>GAGGCTGGGTATTTCCCGGCCTTTCTGTTATCCGAAATCCACTGAAAGCACAGCGGCTGGCTGAGGAGATAA<br>ATAATAAACGAGGGGCTGTATGCACAAAGCATCTTCTGTTGAGTTAAGAACGAGTATCGAGATGGCACATA<br>GCCTTGCTCAAATTGGAATCAGGTTTGTGCCAATACCAGTAGAAACAGACGAAGAA	12
rgnB	Terminator	GATGGTAGTGTGGGGTCTCCCCATGCGAGAGTAGGGAACTGCCAGGCATCAAATAAAACGAAAGGCTCAGT<br>CGAAAGACTGGGCCTTTCGTTTTATCTGTTGTTTGTCGGTGAACGCTCTCCTGAGTAGGACAAATCCGCCGG<br>GAGCGGATTTGAACGTTGCGAAGCAACGGCCCGGAGGGTGGCGGGCAGGACGCCCGCCATAAACTGCCAG<br>GCATCAAATTAAGCAGAAGGCCATCCTGACGGATGGCCTTTTTGCGT	12
rrnB T1	Terminator	ATTTGTCCTACTCAGGAGAGCGTTCACCGACAAACAACAGATAAAACGAAAGGCCCAGTCTTTCGACTGAGC<br>CTTTCGTTTTATTTG	7
Lambda t0	Terminator	GACTCCTGTTGATAGATCCAGTAATGACCTCAGAACTCCATCTGGATTTGTTCAGAACGCTCGGTTGCCGCCG<br>GGCGTTTTTTATTGGTGAGAAT	7
His operon terminator	Terminator	TCCGGCAAAAAAGGGCAAGGTGTCACCACCCTGCCCTTTTTCTTTAAAACCGAAAAGA	13
LuxR	Gene	ATGAAAAACATAAATGCCGACGACACATACAGAATAATTAATAAAATTAAAGCTTGTAGAAGCAATAATGAT<br>ATTAATCAATGCTTATCTGATATGACTAAAATGGTACATTGTGAATATTATTTACTCGCGATCATTTATCCTCA<br>TTCTATGGTTAAATCTGATATTTCAATCCTAGATAATTACCCTAAAAAATGGAGGCAATATTATGATGACGCT<br>AATTTAATAAAATATGATCCTATAGTAGATTATTCTAACTCCAATCATTCACCAATTAATTGGAATATATTTGA<br>AAACAATGCTGTAAATAAAAAATCTCCAAATGTAATTAAAGAAGCGAAAACATCAGGTCTTATCACTGGGTT<br>TAGTTTCCCTATTCATACGGCTAACAATGGCTTCGGAATGCTTAGTTTTGCACATTCAGAAAAAGACAACTAT<br>ATAGATAGTTTATTTTTACATGCGTGTATGAACATACCATTAATTGTTCCTTCTCTAGTTGATAATTATCGAAA<br>AATAAATATAGCAAATAATAAATCAAACAACGATTTAACCAAAAGAGAAAAAGAATGTTTAGCGTGGGCAT<br>GCGAAGGAAAAAGCTCTTGGGATATTTCAAAAATATTAGGTTGCAGTGAGCGTACTGTCACTTTCCATTTAA<br>CCAATGCGCAAATGAAACTCAATACAACAAACCGCTGCCAAAGTATTTCTAAAGCAATTTTAACAGGAGCAA<br>TTGATTGCCCATACTTTAAAAATTAA	8
RpaR	Gene	ATGATCGTCGGCGAAGATCAGCTTTGGGGACGGCGTGCGCTGGAATTCGTCGATTCCGTCGAACGGCTCGA<br>GGCGCCGGCGCTGATCAGCCGGTTCGAATCGCTGATCGCGAGCTGCGGATTTACCGCCTACATCATGGCCG<br>GCCTGCCGTCGCGCAATGCCGGACTACCGGAGCTGACGCTGGCCAATGGCTGGCCGCGAGACTGGTTCGAT<br>CTGTATGTCAGCGAAAACTTCAGCGCGGTCGATCCGGTGCCGCGCCACGGCGCTACCACGGTTCATCCTTTC<br>GTATGGTCCGATGCACCCTACGACCGCGACCGTGATCCGGCCGCCCACCGGGTCATGACCCGGGCGGCGGA<br>ATTCGGACTGGTCGAGGGTTACTGCATTCCGCTGCACTACGACGACGGTAGCGCCGCGATCAGCATGGCCG<br>GCAAGGATCCGGACCTCAGCCCGGCCGCGCGCGGCGCGATGCAGCTGGTCAGCATCTACGCGCATAGTCGC<br>CTGCGCGCACTCAGCCGGCCAAAGCCGATCCGGCGCAACCGGCTCACGCCGCGCGAGTGCGAGATCCTGCA<br>ATGGGCAGCGCAGGGCAAGACCGCCTGGGAAATCTCGGTAATCCTCTGCATCACCGAACGCACGGTGAAAT<br>TCCATCTGATCGAAGCCGCCCGCAAGCTCGACGCCGCCAACCGCACCGCGGCGGTTGCCAAGGCATTGACG<br>CTCGGATTGATCCGTTTGTAA	6
LuxI	Gene	ATGACTATAATGATAAAAAAATCGGATTTTTTGGCAATTCCATCGGAGGAGTATAAAGGTATTCTAAGTCTTC<br>GTTATCAAGTGTTTAAGCAAAGACTTGAGTGGGACTTAGTTGTAGAAAATAACCTTGAATCAGATGAGTATG<br>ATAACTCAAATGCAGAATATATTTATGCTTGTGATGATACTGAAAATGTAAGTGGATGCTGGCGTTTATTACC<br>TACAACAGGTGATTATATGCTGAAAAGTGTTTTTCCTGAATTGCTTGGTCAACAGAGTGCTCCCAAAGATCCT<br>AATATAGTCGAATTAAGTCGTTTTGCTGTAGGTAAAAATAGCTCAAAGATAAATAACTCTGCTAGTGAAATTA<br>CAATGAAACTATTTGAAGCTATATATAAACACGCTGTTAGTCAAGGTATTACAGAATATGTAACAGTAACATC<br>AACAGCAATAGAGCGATTTTTAAAGCGTATTAAAGTTCCTTGTCATCGTATTGGAGACAAAGAAATTCATGT<br>ATTAGGTGATACTAAATCGGTTGTATTGTCTATGCCTATTAATGAACAGTTTAAAAAAGCAGTCTTAA	iGEM C0061
RpaI	Gene	ATGCAGGTTCATGTCATCCGTCGAGAGAACCGCGCGCTCTATGCCGGTCTGCTCGAAAAGTACTTCCGCATC<br>CGTCACCAGATCTACGTCGTCGAGCGCGGCTGGAAGGAGCTCGATCGGCCGGATGGCCGCGAGATCGATCA<br>GTTCGACACCGAAGACGCCGTGTATCTGCTCGGCGTCGACAATGACGACATCGTCGCCGGCATGCGGATGG<br>TGCCGACCACGTCACCGACGCTCCTCAGCGACGTCTTCCCGCAGCTTGCGCTGGCAGGCCCGGTGCGGCGG<br>CCGGATGCCTACGAGCTGTCGCGGATCTTCGTGGTACCGCGCAAGCGCGGCGAGCATGGCGGCCCGCGCGC<br>CGAAGCCGTGATCCAGGCGGCCGCGATGGAGTACGGCCTGTCGATCGGTCTGTCGGCCTTCACCATCGTGC<br>TGGAGACCTGGTGGCTGCCGCGACTGGTGGACCAGGGCTGGAAGGCAAAGCCGCTCGGCCTGCCTCAGGA<br>CATCAACGGATTCTCGACCACCGCAGTGATCGTCGACGTCGACGACGACGCCTGGGTCGGCATCTGCAATCG<br>CCGCTCGGTGCCCGGACCCACGCTGGAATGGCGCGGGCTCGAAGCCATCCGCCGTCATTCGCTTCCGGAATT<br>CCAGGTGATTTCATAA	CIDAR MoClo Extension[1]
TAL	Gene	ATGACCCAGGTGGTTGAACGCCAGGCCGATCGCCTGAGTAGTCGTGAATACTTAGCTCGCGTCGTTCGTAGT<br>GCCGGCTGGGATGCGGGCCTAACCTCTTGTACAGATGAAGAAATTGTTCGCATGGGCGCGTCAGCCCGCAC<br>CATCGAGGAATATTTAAAAAGTGATAAACCGATTTATGGTTTAACCCAAGGCTTCGGCCCGCTGGTACTGTTT<br>GATGCGGATAGCGAATTAGAACAGGGTGGTAGCCTGATTAGCCATCTGGGCACCGGTCAGGGCGCGCCGC<br>TGGCGCCGGAAGTGAGTCGTTTAATTCTGTGGCTGCGTATTCAAAACATGCGCAAAGGTTATAGCGCCGTTA<br>GCCCGGTTTTCTGGCAAAAACTGGCAGACCTATGGAATAAAGGCTTTACCCCGGCAATTCCGCGTCATGGTA<br>CCGTTTCCGCCTCGGGTGATCTGCAACCGCTGGCGCATGCCGCGCTGGCATTCACCGGCGTGGGTGAAGCG<br>TGGACCCGCGATGCAGATGGCCGCTGGAGCACCGTTCCGGCTGTTGATGCCCTGGCAGCGCTGGGTGCCGA<br>ACCGTTTGATTGGCCTGTCCGCGAAGCACTGGCGTTTGTTAATGGCACCGGAGCCAGCCTGGCGGTTGCTGT<br>TTTAAATCATCGTTCTGCCCTGCGCCTGGTTCGCGCGTGTGCGGTACTGAGCGCACGCCTGGCGACCCTGCT<br>GGGCGCAAATCCGGAACATTATGACGTTGGTCATGGCGTTGCCCGCGGTCAGGTTGGCCAGCTGACCGCGG<br>CGGAATGGATTCGTCAGGGCCTGCCACGTGGTATGGTGCGCGATGGAAGCCGTCCGTTGCAGGAACCTTAT<br>AGCCTTCGCTGCGCTCCGCAGGTTCTAGGCGCTGTTCTGGATCAGCTGGACGGTGCGGGTGACGTGCTGGC	14

[1] CIDAR MoClo Extension, Volume I was a gift from Richard Murray (Addgene kit #1000000161).

		CCGCGAAGTTGATGGTTGCCAGGATAACCCTATTACCTACGAAGGTGAATTGCTGCATGGCGGTAACTTCCA<br>TGCCATGCCGGTTGGTTTTGCAAGTGATCAGATTGGTCTGGCGATGCACATGGCGGCCTACCTGGCTGAACG<br>CCAGCTGGGCCTGCTGGTTAGCCCGGTAACCAATGGTGATTTACCACCGATGCTGACCCCGCGTGCCGGCCG<br>TGGTGCGGGTCTTGCTGGCGTCCAGATTTCTGCCACCAGCTTCGTTTCTCGTATTCGCCAACTGGTTTTCCCG<br>GCGTCTCTGACCACCCTGCCGACCAACGGTTGGAATCAAGACCATGTACCGATGGCACTGAATGGCGCTAAT<br>AGCGTTTTCGAAGCACTGGAACTGGGTTGGTTAACCGTTGGAAGCCTGGCGGTGGGCGTTGCACAGCTGGC<br>GGCGATGACCGGTCATGCGGCTGAAGGGGTTTGGGCAGAACTGGCAGGCATTTGCCCGCCGTTAGATGCC<br>GACCGTCCGCTGGGTGCGGAAGTTCGCGCAGCCCGTGATCTGCTGAGCGCGCACGCTGATCAGCTGTTGGT<br>GGACGAAGCCGATGGTAAAGACTTTGGCTAA	
4CL2nt	Gene	ATGGAGAAAGACACGAAGCAAGTTGACATCATTTTTCGCTCGAAACTGCCGGACATTTACATTCCGAATCAT<br>CTGCCGCTGCATAGCTACTGCTTCGAGAACATTTCTGAATTTTCTAGCCGTCCGTGTCTGATTAACGGTGCCA<br>ATAAACAGATCTATACGTACGCGGACGTCGAGTTGAACAGCCGTAAGGTCGCAGCGGGTCTGCACAAGCAA<br>GGCATCCAGCCTAAAGATACCATCATGATTCTGTTGCCAAATTCTCCGGAGTTTGTGTTTGCGTTTATCGGCG<br>CAAGCTACCTGGGTGCGATTAGCACGATGGCAAATCCGCTGTTTACCCCGGCTGAGGTTGTTAAACAAGCAA<br>AAGCCAGCAGCGCGAAGATCATCGTGACCCAAGCATGCCACGTCAACAAAGTTAAGGACTATGCCTTCGAA<br>AATGACGTCAAGATCATTTGCATCGATAGCGCGCCTGAAGGTTGTCTGCATTTCAGCGTTCTGACGCAGGCT<br>AACGAACACGATATTCCGGAAGTTGAGATTCAGCCGGACGATGTGGTGGCCCTGCCGTACTCCAGCGGTAC<br>CACCGGCCTGCCGAAAGGCGTTATGCTGACCCACAAGGGCCTGGTGACGAGCGTCGCCCAGCAGGTCGATG<br>GTGAAAACCCGAACCTGTACATCCACAGCGAAGATGTTATGCTGTGTGTTCTGCCACTGTTCCACATCTATTC<br>CCTGAACAGCGTCCTGCTGTGCGGCCTGCGTGTGGGCGCTGCCATTTTGATTATGCAGAAGTTTGACATTGT<br>CAGCTTCTTGGAACTGATCCAACGCTACAAGGTGACGATCGGTCCGTTCGTCCCGCCGATTGTTTTGGCCATT<br>GCAAAAAGCCCAATGGTGGATGACTATGACCTGTCGAGCGTGCGTACCGTGATGTCCGGTGCAGCGCCGCT<br>GGGCAAAGAGCTGGAGGATACCGTTCGTGCGAAGTTTCCGAATGCGAAACTGGGTCAAGGCTACGGTATG<br>ACTGAAGCAGGTCCGGTGCTGGCGATGTGCTTGGCGTTCGCGAAAGAGCCGTTCGAAATCAAAAGCGGTGC<br>GTGCGGTACCGTGGTGCGTAATGCTGAAATGAAAATTGTGGATCCGAAAACCGGCAACAGCCTGCCGCGCA<br>ACCAGAGCGGTGAGATTTGTATTCGCGGTGACCAGATTATGAAGGGCTACCTGAATGACCCGGAGGCCACT<br>GCGCGTACGATCGACAAAGAGGGTTGGCTGTATACCGGCGACATCGGTTATATCGATGACGACGACGAGCT<br>GTTCATCGTTGATCGCCTGAAAGAGTTGATTAAGTACAAGGGTTTCCAAGTTGCGCCTGCGGAACTGGAGGC<br>TCTGCTGTTGAATCATCCGAACATTAGCGATGCAGCAGTCGTTCCGATGAAGGATGAGCAGGCGGGTGAAG<br>TTCCGGTCGCGTTTGTTGTGCGTAGCAACGGCAGCACGATCACCGAGGATGAGGTAAAGGATTTCATTTCCA<br>AACAAGTCATCTTCTATAAGCGTATCAAGCGTGTGTTTTTCGTCGATGCAATCCCGAAAAGCCCGTCCGGTAA<br>GATCCTGCGCAAAGACTTGCGTGGAAGCTGGCGGCAGGTCTGCCGAATTAGTAA	14
dCas9	Gene	ATGGATAAGAAATACTCAATAGGCTTAGCTATCGGCACAAATAGCGTCGGATGGGCGGTGATCACTGATGA<br>ATATAAGGTTCCGTCTAAAAAGTTCAAGGTTCTGGGAAATACAGACCGCCACAGTATCAAAAAAAATCTTAT<br>AGGGGCTCTTTTATTTGACAGTGGAGAGACAGCGGAAGCGACTCGTCTCAAACGGACAGCTCGTAGAAGGT<br>ATACACGTCGGAAGAATCGTATTTGTTATCTACAGGAGATTTTTTCAAATGAGATGGCGAAAGTAGATGATA<br>GTTTCTTTCATCGACTTGAAGAGTCTTTTTTGGTGGAAGAAGACAAGAAGCATGAACGTCATCCTATTTTTGG<br>AAATATAGTAGATGAAGTTGCTTATCATGAGAAATATCCAACTATCTATCATCTGCGAAAAAAATTGGTAGAT<br>TCTACTGATAAAGCGGATTTGCGCTTAATCTATTTGGCCTTAGCGCATATGATTAAGTTTCGTGGTCATTTTTT<br>GATTGAGGGAGATTTAAATCCTGATAATAGTGATGTGGACAAACTATTTATCCAGTTGGTACAAACCTACAA<br>TCAATTATTTGAAGAAAACCCTATTAACGCAAGTGGAGTAGATGCTAAAGCGATTCTTTCTGCACGATTGAGT<br>AAATCAAGACGATTAGAAAATCTCATTGCTCAGCTCCCCGGTGAGAAGAAAAATGGCTTATTTGGGAATCTC<br>ATTGCTTTGTCATTGGGTTTGACCCCTAATTTTAAATCAAATTTTGATTTGGCAGAAGATGCTAAATTACAGCT<br>TTCAAAAGATACTTACGATGATGATTTAGATAATTTATTGGCGCAAATTGGAGATCAATATGCTGATTTGTTT<br>TTGGCAGCTAAGAATTTATCAGATGCTATTTTACTTTCAGATATCCTAAGAGTAAATACTGAAATAACTAAGG<br>CTCCCCTATCAGCTTCAATGATTAAACGCTACGATGAACATCATCAAGACTTGACTCTTTTAAAAGCTTTAGTT<br>CGACAACAACTTCCAGAAAAGTATAAAGAAATCTTTTTTGATCAATCAAAAAACGGATATGCAGGTTATATT<br>GATGGGGGAGCTAGCCAAGAAGAATTTTATAAATTTATCAAACCAATTTTAGAAAAAATGGATGGTACTGA<br>GGAATTATTGGTGAAACTAAATCGTGAAGATTTGCTGCGCAAGCAACGGACCTTTGACAACGGCTCTATTCC<br>CCATCAAATTCACTTGGGTGAGCTGCATGCTATTTTGAGAAGACAAGAAGACTTTTATCCATTTTTAAAAGAC<br>AATCGTGAGAAGATTGAAAAAATCTTGACTTTTCGAATTCCTTATTATGTTGGTCCATTGGCGCGTGGCAATA<br>GTCGTTTTGCATGGATGACTCGGAAGTCTGAAGAAACAATTACCCCATGGAATTTTGAAGAAGTTGTCGATA<br>AAGGTGCTTCAGCTCAATCATTTATTGAACGCATGACAAACTTTGATAAAAATCTTCCAAATGAAAAAGTACT<br>ACCAAAACATAGTTTGCTTTATGAGTATTTTACGGTTTATAACGAATTGACAAAGGTCAAATATGTTACTGAA<br>GGAATGCGAAAACCAGCATTTCTTTCAGGTGAACAGAAGAAAGCCATTGTTGATTTACTCTTCAAAACAAAT<br>CGAAAAGTAACCGTTAAGCAATTAAAAGAAGATTATTTCAAAAAAATAGAATGTTTTGATAGTGTTGAAATT<br>TCAGGAGTTGAAGATAGATTTAATGCTTCATTAGGTACCTACCATGATTTGCTAAAAATTATTAAAGATAAAG<br>ATTTTTTGGATAATGAAGAAAATGAAGATATCTTAGAGGATATTGTTTTAACATTGACCTTATTTGAAGATAG<br>GGAGATGATTGAGGAAAGACTTAAAACATATGCTCACCTCTTTGATGATAAGGTGATGAAACAGCTTAAACG<br>TCGCCGTTATACTGGTTGGGGACGTTTGTCTCGAAAATTGATTAATGGTATTAGGGATAAGCAATCTGGCAA<br>AACAATATTAGATTTTTTGAAATCAGATGGTTTTGCCAATCGCAATTTTATGCAGCTGATCCATGATGATAGT<br>TTGACATTTAAAGAAGACATTCAAAAAGCACAAGTGTCTGGACAAGGCGATAGTTTACATGAACATATTGCA<br>AATTTAGCTGGTAGCCCTGCTATTAAAAAAGGTATTTTACAGACTGTAAAAGTTGTTGATGAATTGGTCAAA<br>GTAATGGGGCGGCATAAGCCAGAAAATATCGTTATTGAAATGGCACGTGAAAATCAGACAACTCAAAAGGG<br>CCAGAAAAATTCGCGAGAGCGTATGAAACGAATCGAAGAAGGTATCAAAGAATTAGGAAGTCAGATTCTTA<br>AAGAGCATCCTGTTGAAAATACTCAATTGCAAAATGAAAAGCTCTATCTCTATTATCTCCAAAATGGAAGAG<br>ACATGTATGTGGACCAAGAATTAGATATTAATCGTTTAAGTGATTATGATGTCGATGCCATTGTTCCACAAAG<br>TTTCCTTAAAGACGATTCAATAGACAATAAGGTCTTAACGCGTTCTGATAAAAATCGTGGTAAATCGGATAAC<br>GTTCCAAGTGAAGAAGTAGTCAAAAAGATGAAAAACTATTGGAGACAACTTCTAAACGCCAAGTTAATCACT<br>CAACGTAAGTTTGATAATTTAACGAAAGCTGAACGTGGAGGTTTGAGTGAACTTGATAAAGCTGGTTTTATC<br>AAACGCCAATTGGTTGAAACTCGCCAAATCACTAAGCATGTGGCACAAATTTTGGATAGTCGCATGAATACT<br>AAATACGATGAAAATGATAAACTTATTCGAGAGGTTAAAGTGATTACCTTAAAATCTAAATTAGTTTCTGACT<br>TCCGAAAAGATTTCCAATTCTATAAAGTACGTGAGATTAACAATTACCATCATGCCCATGATGCGTATCTAAA<br>TGCCGTCGTTGGAACTGCTTTGATTAAGAAATATCCAAAACTTGAATCGGAGTTTGTCTATGGTGATTATAAA<br>GTTTATGATGTTCGTAAAATGATTGCTAAGTCTGAGCAAGAAATAGGCAAAGCAACCGCAAAATATTTCTTTT<br>ACTCTAATATCATGAACTTCTTCAAAACAGAAATTACACTTGCAAATGGAGAGATTCGCAAACGCCCTCTAAT<br>CGAAACTAATGGGGAAACTGGAGAAATTGTCTGGGATAAAGGGCGAGATTTTGCCACAGTGCGCAAAGTAT<br>TGTCCATGCCCCAAGTCAATATTGTCAAGAAAACAGAAGTACAGACAGGCGGATTCTCCAAGGAGTCAATTT<br>TACCAAAAAGAAATTCGGACAAGCTTATTGCTCGTAAAAAAGACTGGGATCCAAAAAAATATGGTGGTTTTG<br>ATAGTCCAACGGTAGCTTATTCAGTCCTAGTGGTTGCTAAGGTGGAAAAAGGGAAATCGAAGAAGTTAAAA<br>TCCGTTAAAGAGTTACTAGGGATCACAATTATGGAAAGAAGTTCCTTTGAAAAAAATCCGATTGACTTTTTAG<br>AAGCTAAAGGATATAAGGAAGTTAAAAAAGACTTAATCATTAAACTACCTAAATATAGTCTTTTTGAGTTAG<br>AAAACGGTCGTAAACGGATGCTGGCTAGTGCCGGAGAATTACAAAAAGGAAATGAGCTGGCTCTGCCAAGC<br>AAATATGTGAATTTTTTATATTTAGCTAGTCATTATGAAAAGTTGAAGGGTAGTCCAGAAGATAACGAACAA<br>AAACAATTGTTTGTGGAGCAGCATAAGCATTATTTAGATGAGATTATTGAGCAAATCAGTGAATTTTCTAAGC<br>GTGTTATTTTAGCAGATGCCAATTTAGATAAAGTTCTTAGTGCATATAACAAACATAGAGACAAACCAATACG<br>TGAACAAGCAGAAAATATTATTCATTTATTTACGTTGACGAATCTTGGAGCTCCCGCTGCTTTTAAATATTTTG<br>ATACAACAATTGATCGTAAACGATATACGTCTACAAAAGAAGTTTTAGATGCCACTCTTATCCATCAATCCAT<br>CACTGGTCTTTATGAAACACGCATTGATTTGAGTCAGCTAGGAGGTGACTAA	4
VioB	Gene	ATGAGCATTCTGGATTTCCCGCGTATCCACTTCCGTGGCTGGGCCCGTGTCAATGCGCCGACCGCGAACCGC<br>GATCCGCACGGCCACATCGATATGGCCAGCAATACCGTGGCGATGGCGGGTGAGCCGTTCGACCTGGCACG<br>CCATCCTACGGAGTTCCACCGTCACCTGCGCTCCCTGGGTCCGCGCTTCGGCTTGGATGGTCGTGCTGACCC	13

		GGAAGGCCCGTTCAGCCTGGCCGAGGGCTACAACGCTGCCGGTAACAACCACTTTTCGTGGGAGAGCGCAA<br>CCGTTAGCCACGTGCAATGGGATGGCGGTGAGGCGGATCGTGGTGACGGTCTGGTCGGTGCTCGTTTGGCA<br>CTGTGGGGTCACTACAATGATTATCTGCGTACCACCTTCAATCGTGCTCGTTGGGTCGACAGCGACCCGACG<br>CGCCGTGACGCTGCACAAATCTATGCGGGCCAATTCACCATTAGCCCGGCTGGTGCCGGTCCGGGTACGCC<br>GTGGCTGTTTACGGCAGACATTGATGATAGCCATGGTGCACGTTGGACGCGTGGCGGCCACATTGCAGAGC<br>GTGGCGGCCACTTCTTGGATGAAGAGTTTGGTCTGGCACGCCTGTTTCAGTTCTCTGTGCCGAAAGATCACC<br>CACATTTTCTGTTTCACCCGGGTCCGTTTGATTCCGAGGCCTGGCGTCGTCTGCAATTGGCTCTGGAGGATGA<br>CGACGTTCTGGGTCTGACCGTGCAATATGCGTTGTTCAATATGAGCACCCCGCCTCAGCCGAACAGCCCGGT<br>TTTTCACGATATGGTCGGTGTTGTCGGTCTGTGGCGTCGTGGTGAACTGGCGAGCTACCCGGCTGGTCGTCT<br>GCTGCGTCCGCGTCAACCGGGTCTGGGTGACCTGACCCTGCGCGTCAACGGTGGTCGCGTTGCGCTGAATTT<br>GGCGTGTGCCATTCCGTTCAGCACTCGTGCCGCGCAGCCAAGCGCACCGGACCGCCTGACCCCGGACCTGG<br>GTGCCAAACTGCCGCTGGGCGATCTGCTGCTGCGTGATGAGGACGGCGCACTGTTGGCACGTGTGCCGCAG<br>GCTCTGTACCAAGACTATTGGACGAATCACGGTATTGTGGACCTGCCGCTGCTGCGCGAACCGCGTGGTAGC<br>TTGACCCTGAGCAGCGAACTGGCGGAGTGGCGTGAGCAAGACTGGGTCACCCAAAGCGACGCGTCTAACCT<br>GTACCTGGAGGCACCGGATCGCCGTCACGGTCGCTTTTTCCCTGAGAGCATCGCGCTGCGCAGCTACTTTCG<br>CGGTGAAGCGCGTGCGCGTCCGGATATCCCGCATCGTATCGAGGGCATGGGCCTGGTCGGCGTCGAATCTC<br>GTCAGGATGGCGACGCTGCGGAATGGCGTCTGACGGGTCTGCGTCCGGGTCCGGCACGCATTGTTCTGGAC<br>GATGGTGCCGAGGCGATCCCTCTGCGTGTTCTGCCTGACGATTGGGCGCTGGATGACGCGACCGTCGAAGA<br>AGTGGATTACGCCTTTTTGTACCGCCACGTTATGGCGTATTACGAGCTGGTGTATCCATTCATGAGCGACAA<br>GGTGTTTTCCCTGGCTGATCGTTGCAAATGTGAAACGTACGCACGTCTGATGTGGCAGATGTGTGATCCGCA<br>GAACCGCAACAAGTCCTATTACATGCCGAGCACCCGCGAACTGTCGGCACCGAAAGCTCGTTTGTTCTTGAA<br>GTATCTGGCCCACGTGGAAGGCCAGGCACGCCTGCAAGCACCTCCGCCAGCGGGTCCGGCACGCATTGAAT<br>CTAAAGCCCAGTTGGCGGCAGAGCTGCGTAAAGCCGTCGACCTGGAGCTGTCTGTGATGCTGCAATACCTG<br>TACGCGGCGTATAGCATTCCGAACTATGCACAGGGCCAACAACGTGTTCGTGACGGTGCGTGGACCGCCGA<br>GCAGCTGCAACTGGCGTGCGGTAGCGGTGACCGTCGCCGTGATGGCGGTATTCGTGCAGCACTGCTGGAAA<br>TTGCTCATGAAGAAATGATTCATTACCTGGTCGTTAACAACCTGCTGATGGCCCTGGGCGAGCCGTTCTACG<br>CGGGTGTCCCGCTGATGGGCGAAGCGGCACGTCAGGCGTTTGGCCTGGACACCGAGTTCGCTCTGGAACCG<br>TTTAGCGAAAGCACGCTGGCACGTTTTGTTCGTCTGGAATGGCCGCACTTTATCCCAGCACCGGGCAAATCC<br>ATCGCGGACTGCTATGCCGCCATTCGTCAGGCGTTTTTGGATCTGCCGGACTTGTTTGGTGGCGAGGCAGGT<br>AAGCGTGGCGGTGAACACCACCTGTTCCTGAATGAGCTGACCAACCGTGCGCATCCGGGTTATCAACTGGA<br>AGTTTTCGATCGCGACTCGGCGCTGTTTGGTATTGCATTTGTGACCGATCAGGGCGAAGGTGGCGCTCTGGA<br>CAGCCCGCACTACGAACATAGCCATTTTCAACGTCTGCGTGAAATGAGCGCGCGTATCATGGCTCAAAGCGC<br>ACCGTTCGAACCGGCGCTGCCGGCGTTGCGTAATCCGGTTCTGGATGAGAGCCCGGGTTGCCAACGTGTCG<br>CAGACGGTCGTGCGCGTGCGCTGATGGCATTGTACCAAGGCGTTTATGAGCTGATGTTTGCGATGATGGCG<br>CAGCACTTCGCCGTGAAACCGCTGGGTAGCTTGCGTCGCAGCCGCCTGATGAACGCAGCAATCGATCTGAT<br>GACCGGTCTGTTGCGTCCGCTGAGCTGCGCGCTGATGAACCTGCCAAGCGGCATCGCCGGTCGCACGGCCG<br>GTCCGCCGCTGCCGGGTCCGGTTGACACCCGTAGCTATGACGACTACGCGCTGGGCTGTCGCATGCTGGCA<br>CGCCGTTGCGAGCGTCTGCTGGAGCAGGCGAGCATGCTGGAACCGGGTTGGCTGCCGGATGCGCAGATGG<br>AGCTGCTGGATTTCTATCGTCGCCAAATGCTGGACTTGGCGTGCGGCAAACTGA	
mScarlet-I	Gene	ATGGTTAGCAAAGGCGAGGCGGTTATCAAGGAGTTTATGCGTTTTAAGGTTCACATGGAGGGTAGCATGAA<br>TGGTCACGAGTTCGAGATCGAGGGTGAAGGCGAGGGTCGTCCGTACGAAGGCACCCAGACCGCGAAGCTG<br>AAAGTGACCAAGGGTGGCCCGCTGCCGTTCAGCTGGGACATCCTGAGCCCGCAGTTCATGTATGGCAGCCG<br>TGCGTTTATCAAACACCCGGCGGACATTCCGGATTACTATAAGCAAAGCTTCCCGGAAGGTTTTAAATGGGA<br>GCGTGTTATGAACTTCGAAGATGGTGGCGCGGTGACCGTTACCCAGGACACCAGCCTGGAGGATGGCACCC<br>TGATTTACAAGGTGAAACTGCGTGGCACCAACTTTCCGCCGGATGGTCCGGTTATGCAGAAGAAAACGATG<br>GGTTGGGAAGCGAGCACCGAGCGTCTGTATCCGGAAGATGGCGTGCTGAAGGGTGATATCAAAATGGCGC<br>TGCGTCTGAAGGACGGTGGCCGTTACCTGGCGGATTTTAAGACCACCTATAAAGCGAAGAAACCGGTGCAA<br>ATGCCGGGTGCGTACAACGTTGACCGTAAACTGGATATTACCAGCCACAACGAGGATTATACCGTGGTTGA<br>GCAATATGAGCGTAGCGAGGGTCGCCACAGCACCGGCGGCATGGACGAACTGTATAAGTGA	CIDAR MoClo Extension
mCherry	Gene	ATGGTGAGCAAGGGCGAGGAGGATAACATGGCCATCATCAAGGAGTTCATGCGCTTCAAGGTTCACATGGA<br>GGGCTCCGTGAACGGCCACGAGTTCGAGATCGAGGGCGAGGGCGAGGGCCGCCCCTACGAGGGCACCCAG<br>ACCGCCAAGCTGAAGGTGACCAAGGGTGGCCCCCTGCCCTTCGCCTGGGACATCCTGTCCCCTCAGTTCATG<br>TACGGCTCCAAGGCCTACGTGAAGCACCCCGCCGACATCCCCGACTACTTGAAGCTGTCCTTCCCCGAGGGC<br>TTCAAGTGGGAGCGCGTGATGAACTTCGAGGACGGCGGCGTGGTGACCGTGACCCAGGACTCCTCCCTGCA<br>AGACGGCGAGTTCATCTACAAGGTGAAGCTGCGCGGCACCAACTTCCCCTCCGACGGCCCCGTAATGCAGA<br>AGAAGACTATGGGCTGGGAGGCCTCCTCCGAGCGGATGTACCCCGAGGACGGCGCGCTGAAGGGCGAGAT<br>CAAGCAGAGGCTGAAGCTGAAGGACGGCGGCCACTACGACGCTGAGGTCAAGACCACCTACAAGGCCAAG<br>AAGCCCGTGCAACTGCCCGGCGCGTACAACGTCAACATCAAGTTGGACATCACCTCCCACAACGAGGACTAC<br>ACCATCGTGGAACAGTACGAACGCGCCGAGGGCCGCCACTCCACCGGCGGCATGGACGAGCTGTATAAGTA<br>A	4
mRFP1	Gene	ATGGTGAGCAAGGGCGAGGAGGATAACATGGCCATCATCAAGGAGTTCATGCGCTTCAAGGTTCACATGGA<br>GGGCTCCGTGAACGGCCACGAGTTCGAGATCGAGGGCGAGGGCGAGGGCCGCCCCTACGAGGGCACCCAG<br>ACCGCCAAGCTGAAGGTGACCAAGGGTGGCCCCCTGCCCTTCGCCTGGGACATCCTGTCCCCTCAGTTCATG<br>TACGGCTCCAAGGCCTACGTGAAGCACCCCGCCGACATCCCCGACTACTTGAAGCTGTCCTTCCCCGAGGGC<br>TTCAAGTGGGAGCGCGTGATGAACTTCGAGGACGGCGGCGTGGTGACCGTGACCCAGGACTCCTCCCTGCA<br>AGACGGCGAGTTCATCTACAAGGTGAAGCTGCGCGGCACCAACTTCCCCTCCGACGGCCCCGTAATGCAGA<br>AGAAGACTATGGGCTGGGAGGCCTCCTCCGAGCGGATGTACCCCGAGGACGGCGCGCTGAAGGGCGAGAT<br>CAAGCAGAGGCTGAAGCTGAAGGACGGCGGCCACTACGACGCTGAGGTCAAGACCACCTACAAGGCCAAG<br>AAGCCCGTGCAACTGCCCGGCGCGTACAACGTCAACATCAAGTTGGACATCACCTCCCACAACGAGGACTAC<br>ACCATCGTGGAACAGTACGAACGCGCCGAGGGCCGCCACTCCACCGGCGGCATGGACGAGCTGTATAAGTA<br>A	8
mKate	Gene	ATGGAACTGATTAAAGAAAACATGCATATGAAACTGTATATGGAAGGCACCGTGAACAACCATCACTTTAAA<br>TGTACCAGCGAAGGTGAAGGTAAACCGTATGAAGGCACCCAGACCATGCGTATTAAAGCAGTTGAAGGTGG<br>TCCGCTGCCGTTTGCATTTGATATTCTGGCAACCAGCTTTATGTATGGCAGCAAAACCTTTATTAACCATACCC<br>AGGGTATCCCGGATTTTTTCAAACAGAGCTTTCCGGAAGGTTTTACCTGGGAACGTGTTACCACCTATGAAG<br>ATGGTGGTGTTCTGACCGCAACCCAGGATACCAGTCTGCAGGATGGTTGTCTGATTTATAATGTGAAAATTC<br>GCGGTGTGAACTTTCCGAGCAATGGTCCGGTTATGCAGAAAAAAACCCTGGGTTGGGAAGCAAGCACCGAA<br>ACCCTGTATCCGGCAGATGGTGGCCTGGAAGGTCGTGCAGATATGGCACTGAAACTGGTTGGTGGTGGTCA<br>TCTGATTTGCAATCTGAAAACCACCTATCGTAGCAAAAAACCTGCCAAAAACCTGAAAATGCCTGGCGTTTAT<br>TATGTTGATCGTCGTCTGGAACGTATCAAAGAGGCAGATAAAGAAACCTATGTGGAACAGCATGAAGTTGC<br>AGTTGCACGTTATTGTGATCTGCCGAGCAAACTGGGTCATCGTTAATAA	1
sfGFP	Gene	ATGCGTAAAGGCGAGGAGCTGTTCACTGGTGTCGTCCCTATTCTGGTGGAACTGGATGGTGATGTCAACGG<br>TCATAAGTTTTCCGTGCGTGGCGAGGGTGAAGGTGACGCAACTAATGGTAAACTGACGCTGAAGTTCATCT<br>GTACTACTGGTAAACTGCCGGTACCTTGGCCGACTCTGGTAACGACGCTGACTTATGGTGTTCAGTGCTTTGC<br>TCGTTATCCGGACCATATGAAGCAGCATGACTTCTTCAAGTCCGCCATGCCGGAAGGCTATGTGCAGGAACG<br>CACGATTTCCTTTAAGGATGACGGCACGTACAAAACGCGTGCGGAAGTGAAATTTGAAGGCGATACCCTGG<br>TAAACCGCATTGAGCTGAAAGGCATTGACTTTAAAGAAGACGGCAATATCCTGGGCCATAAGCTGGAATAC<br>AATTTTAACAGCCACAATGTTTACATCACCGCCGATAAACAAAAAAATGGCATTAAAGCGAATTTTAAAATTC<br>GCCACAACGTGGAGGATGGCAGCGTGCAGCTGGCTGATCACTACCAGCAAAACACTCCAATCGGTGATGGT	13

		CCTGTTCTGCTGCCAGACAATCACTATCTGAGCACGCAAAGCGTTCTGTCTAAAGATCCGAACGAGAAACGC<br>GATCATATGGTTCTGCTGGAGTTCGTAACCGCAGCGGGCATCACGCATGGTATGGATGAACTGTACAAATAA	
sfYFP	Gene	ATGCGTAAAGGCGAAGAGCTGTTCACTGGTGTCGTCCCTATTCTGGTGGAACTGGATGGTGATGTCAACGG<br>TCATAAGTTTTCCGTGCGTGGCGAGGGTGAAGGTGACGCAACTAATGGTAAACTGACGCTGAAGTTCATCT<br>GTACTACTGGTAAACTGCCGGTACCTTGGCCGACTCTGGTAACGACGCTGACTTATGGTGTTCAGTGCTTTGC<br>TCGTTATCCGGACCATATGAAGCAGCATGACTTCTTCAAGTCCGCCATGCCGGAAGGCTATGTGCAGGAACG<br>CACGATTTCCTTTAAGGATGACGGCACGTACAAAACGCGTGCGGAAGTGAAATTTGAAGGCGATACCCTGG<br>TAAACCGCATTGAGCTGAAAGGCATTGACTTTAAAGAAGACGGCAATATCCTGGGCCATAAGCTGGAATAC<br>AATTTTAACAGCCACAATGTTTACATCACCGCCGATAAACAAAAAAATGGCATTAAAGCGAATTTTAAAATTC<br>GCCACAACGTGGAGGATGGCAGCGTGCAGCTGGCTGATCACTACCAGCAAAACACTCCAATCGGTGATGGT<br>CCTGTTCTGCTGCCAGACAATCACTATCTGAGCTACCAAAGCGTTCTGTCTAAAGATCCGAACGAGAAACGC<br>GATCATATGGTTCTGCTGGAGTTCGTAACCGCAGCGGGCATCACGCATGGTATGGATGAACTGTACAAATAA	CIDAR MoClo Extension
sfCFP	Gene	ATGCGTAAAGGCGAAGAGCTGTTCACTGGTGTCGTCCCTATTCTGGTGGAACTGGATGGTGATGTCAACGG<br>TCATAAGTTTTCCGTGCGTGGCGAGGGTGAAGGTGACGCAACTAATGGTAAACTGACGCTGAAGTTCATCT<br>GTACTACTGGTAAACTGCCGGTACCTTGGCCGACTCTGGTAACGACGCTGACTTGGGGTGTTCAGTGCTTTG<br>CTCGTTATCCGGACCATATGAAGCAGCATGACTTCTTCAAGTCCGCCATGCCGGAAGGCTATGTGCAGGAAC<br>GCACGATTTCCTTTAAGGATGACGGCACGTACAAAACGCGTGCGGAAGTGAAATTTGAAGGCGATACCCTG<br>GTAAACCGCATTGAGCTGAAAGGCATTGACTTTAAAGAAGACGGCAATATCCTGGGCCATAAGCTGGAATA<br>CAATTACATCAGCGACAATGTTTACATCACCGCCGATAAACAAAAAAATGGCATTAAAGCGAATTTTAAAATT<br>CGCCACAACGTGGAGGATGGCAGCGTGCAGCTGGCTGATCACTACCAGCAAAACACTCCAATCGGTGATGG<br>TCCTGTTCTGCTGCCAGACAATCACTATCTGAGCACGCAAAGCGTTCTGTCTAAAGATCCGAACGAGAAACG<br>CGATCATATGGTTCTGCTGGAGTTCGTAACCGCAGCGGGCATCACGCATGGTATGGATGAACTGTACAAATA<br>A	CIDAR MoClo Extension
gfasPurple	Gene	ATGTCGGTGATTGCTAAACAGATGACCTACAAAGTCTATATGTCGGGTACGGTGAACGGCCATTATTTTGAA<br>GTTGAAGGTGACGGTAAAGGCAAGCCGTATGAAGGCGAACAGACCGTTAAACTGACCGTCACGAAGGGCG<br>GTCCGCTGCCGTTTGCATGGGATATTCTGAGTCCGCAGTCCCAATATGGCAGCATCCCGTTCACGAAATATCC<br>GGAAGATATCCCGGACTACGTGAAGCAGTCTTTTCCGGAAGGTTACACCTGGGAACGTATCATGAACTTCGA<br>AGATGGCGCCGTCTGCACCGTGAGTAACGACAGCTCTATTCAAGGTAATTGTTTCATCTACCATGTCAAGTTC<br>TCAGGTCTGAACTTCCCGCCGAATGGCCCGGTGATGCAGAAAAAGACCCAAGGCTGGGAACCGAATACGGA<br>ACGTCTGTTTGCACGCGATGGTATGCTGATTGGCAACAATTTCATGGCTCTGAAACTGGAAGGCGGTGGCCA<br>CTATCTGTGCGAATTTAAAAGCACCTACAAGGCGAAAAAGCCGGTTAAAATGCCGGGCTATCATTACGTGGA<br>TCGTAAACTGGACGTTACCAACCACAATAAGGACTATACGTCCGTCGAACAGTGTGAAATTTCAATCGCGCG<br>CAAATCGGTGGTTGCCTAATAA	iGEM K1033919
eforRed	Gene	ATGTCAGTGATTAAGCAGGTAATGAAGACCAAGTTGCACCTTGAGGGCACTGTCAATGGCCATGATTTTACG<br>ATCGAGGGTAAAGGTGAAGGCAAGCCGTACGAAGGGTTACAGCACATGAAAATGACAGTCACCAAAGGCG<br>CGCCTCTGCCGTTTTCCGTTCATATTCTTACACCTAGCCACATGTATGGAAGCAAACCGTTTAATAAGTATCCA<br>GCGGATATCCCAGACTACCACAAACAGTCTTTTCCCGAAGGTATGTCTTGGGAGCGGTCGATGATTTTTGAA<br>GATGGTGGCGTATGCACCGCCAGTAATCACTCCAGCATAAACTTGCAAGAGAACTGTTTCATCTATGATGTT<br>AAATTTCATGGTGTGAACCTGCCTCCGGATGGGCCCGTAATGCAAAAAACCATTGCTGGATGGGAGCCGAG<br>CGTGGAAACACTGTACGTGCGTGACGGGATGTTAAAAAGTGACACTGCAATGGTTTTTAAACTGAAAGGAG<br>GCGGTCATCATCGTGTTGATTTCAAAACGACGTATAAAGCCAAAAAACCTGTCAAGCTGCCAGAATTTCATTT<br>CGTTGAACATCGCCTGGAACTGACCAAACACGATAAAGATTTCACAACTTGGGACCAGCAGGAGGCAGCCG<br>AAGGCCATTTCTCACCGCTGCCGAAGGCTCTCCCA	iGEM K592012
cjBlue	Gene	ATGGCTTCCAAAATAAGCGACAACGTACGTATCAAACTGTATATGGAGGGCACGGTTAATAATCACCACTTC<br>ATGTGTGAAGCGGAGGGTGAGGGCAAGCCATACGAAGGAACGCAGATGGAAAACATTAAAGTGACCAAA<br>GGAGGCCCGCTGCCGTTCTCTTTTGATATCCTGACGCCGAACTGCCAATATGGTTCTGTAGCCATAACCAAGT<br>ACACGTCGGGGATTCCGGACTATTTTAAACAGTCATTCCCTGAAGGTTTTACCTGGGAAAGAACCACCATTTA<br>TGAAGATGGGGCTTATCTGACAACTCAGCAGGAAACCAAACTTGATGGAAATTGCTTAGTCTACAATATTAA<br>AATCCTCGGCTGCAATTTTCCCCCAATGGTCCTGTTATGCAGAAAAAAACGCAAGGCTGGGAACCATGTTG<br>CGAGATGCGCTATACACGTGATGGTGTCTTGTGCGGTCAGACATTAATGGCACTGAAATGTGCCGATGGGA<br>ACCATCTGACTTGTCATCTGCGGACTACTTACCGATCCAAAAAGGCAGCGAAGGCGTTGCAAATGCCACCTT<br>TCCATTTTTCAGACCATCGTCCGGAAATTGTGAAGGTTAGCGAGAACGGCACACTGTTTGAGCAGCACGAAA<br>GTAGTGTGGCACGCTATTGTCAGACATGCCCGAGCAAACTTGGTCATAATTAATAA	iGEM K592011
fwYellow	Gene	ATGACGGCACTGACTGAAGGCGCAAAACTGTTCGAGAAAGAAATCCCATATATCACTGAGCTGGAAGGTGA<br>CGTTGAAGGTATGAAGTTTATCATCAAGGGTGAAGGTACCGGTGACGCGAGCGTCGGTAAAGTGGATGCTC<br>AGTTCATTTGTACCACGGGCGACGTTCCGGTTCCGTGGAGCACGCTGGTCACCACGCTGACGTATGGTGCTC<br>AGTGCTTTGCCAAGTATCCGCGCCACATTGCGGATTTCTTCAAAAGCTGCATGCCGGAAGGTTACGTCCAAG<br>AGCGCACCATCACCTTTGAGGGTGATGGCGTGTTCAAGACCCGTGCGGAAGTCACCTTTGAAAATGGCAGC<br>GTGTACAACCGTGTAAAACTGAACGGCCAGGGTTTCAAGAAGGACGGCCACGTGCTGGGCAAAAATCTGGA<br>GTTTAACTTTACCCCTCATTGTTTGTACATTTGGGGTGACCAAGCGAATCATGGCCTGAAGAGCGCGTTCAAA<br>ATCATGCATGAGATCACCGGCTCCAAAGAGGATTTCATTGTTGCCGATCACACCCAAATGAATACCCCGATT<br>GGTGGTGGTCCGGTGCACGTGCCGGAGTACCACCACATTACGTATCATGTTACCCTGTCTAAAGACGTCACC<br>GATCACCGTGACCATTTGAACATTGTTGAGGTGATCAAGGCAGTTGACCTGGAGACGTACCGTTAATAA	iGEM K1033910
CrtE	Gene	ATGACGGTCTGCGCAAAAAAACACGTTCATCTCACTCGCGATGCTGCGGAGCAGTTACTGGCTGATATTGAT<br>CGACGCCTTGATCAGTTATTGCCCGTGGAGGGAGAACGGGATGTTGTGGGTGCCGCGATGCGTGAAGGTG<br>CGCTGGCACCGGGAAAACGTATTCGCCCCATGTTGCTGTTGCTGACCGCCCGCGATCTGGGTTGCGCTGTCA<br>GCCATGACGGATTACTGGATTTGGCCTGTGCGGTGGAAATGGTCCACGCGGCTTCGCTGATCCTTGACGATA<br>TGCCCTGCATGGACGATGCGAAGCTGCGGCGCGGACGCCCTACCATTCATTCTCATTACGGAGAGCATGTG<br>GCAATACTGGCGGCGGTTGCCTTGCTGAGTAAAGCCTTTGGCGTAATTGCCGATGCAGATGGCCTCACGCCG<br>CTGGCAAAAAATCGGGCGGTTTCTGAACTGTCAAACGCCATCGGCATGCAAGGATTGGTTCAGGGTCAGTT<br>CAAGGATCTGTCTGAAGGGGATAAGCCGCGCAGCGCTGAAGCTATTTTGATGACGAATCACTTTAAAACCA<br>GCACGCTGTTTTGTGCCTCCATGCAGATGGCCTCGATTGTTGCGAATGCCTCCAGCGAAGCGCGTGATTGCC<br>TGCATCGTTTTTCACTTGATCTTGGTCAGGCATTTCAACTGCTGGACGATTTGACCGATGGCATGACCGACAC<br>CGGTAAGGATAGCAATCAGGACGCCGGTAAATCGACGCTGGTCAATCTGTTAGGCCCGAGGGCGGTTGAA<br>GAACGTCTGAGACAACATCTTCAGCTTGCCAGTGAGCATCTCTCTGCGGCCTGCCAACACGGGCACGCCACT<br>CAACATTTTATTCAGGCCTGGTTTGACAAAAAACTCGCTGCCGTCAGTTAA	[1]

CrtB	Gene	ATGAATAATCCGTCGTTACTCAATCATGCGGTCGAAACGATGGCAGTTGGCTCGAAAAGTTTTGCGACAGCC<br>TCAAAGTTATTTGATGCAAAAACCCGGCGCAGCGTACTGATGCTCTACGCCTGGTGCCGCCATTGTGACGAT<br>GTTATTGACGATCAGACGCTGGGCTTTCAGGCCCGGCAGCCTGCCTTACAAACGCCCGAACAACGTCTGATG<br>CAACTTGAGATGAAAACGCGCCAGGCCTATGCAGGATCGCAGATGCACGAACCGGCGTTTGCGGCTTTTCA<br>GGAAGTGGCTATGGCTCATGATATCGCCCCGGCTTACGCGTTTGATCATCTGGAAGGCTTCGCCATGGATGT<br>ACGCGAAGCGCAATACAGCCAACTGGATGATACGCTGCGCTATTGCTATCACGTTGCAGGCGTTGTCGGCTT<br>GATGATGGCGCAAATCATGGGCGTGCGGGATAACGCCACGCTGGACCGCGCCTGTGACCTTGGGCTGGCAT<br>TTCAGTTGACCAATATTGCTCGCGATATTGTGGACGATGCGCATGCGGGCCGCTGTTATCTGCCGGCAAGCT<br>GGCTGGAGCATGAAGGTCTGAACAAAGAGAATTATGCGGCACCTGAAAACCGTCAGGCGCTGAGCCGTATC<br>GCCCGTCGTTTGGTGCAGGAAGCAGAACCTTACTATTTGTCTGCCACAGCCGGCCTGGCAGGGTTGCCCCTG<br>CGTTCCGCCTGGGCAATCGCTACGGCGAAGCAGGTTTACCGGAAAATAGGTGTCAAAGTTGAACAGGCCGG<br>TCAGCAAGCCTGGGATCAGCGGCAGTCAACGACCACGCCCGAAAAATTAACGCTGCTGCTGGCCGCCTCTG<br>GTCAGGCCCTTACTTCCCGGATGCGGGCTCATCCTCCCCGCCCTGCGCATCTCTGGCAGCGCCCGCTCTAG	1
CrtI	Gene	ATGAAACCAACTACGGTAATTGGTGCAGGCTTCGGTGGCCTGGCACTGGCAATTCGTCTACAAGCTGCGGG<br>GATCCCCGTCTTACTGCTTGAACAACGTGATAAACCCGGCGGTCGGGCTTATGTCTACGAGGATCAGGGGTT<br>TACCTTTGATGCAGGCCCGACGGTTATCACCGATCCCAGTGCCATTGAAGAACTGTTTGCACTGGCAGGAAA<br>ACAGTTAAAAGAGTATGTCGAACTGCTGCCGGTTACGCCGTTTTACCGCCTGTGTTGGGAGTCAGGGAAGG<br>TCTTTAATTACGATAACGATCAAACCCGGCTCGAAGCGCAGATTCAGCAGTTTAATCCCCGCGATGTCGAAG<br>GTTATCGTCAGTTTCTGGACTATTCACGCGCGGTGTTTAAAGAAGGCTATCTAAAGCTCGGTACTGTCCCTTT<br>TTTATCGTTCAGAGACATGCTTCGCGCCGCACCTCAACTGGCGAAACTGCAGGCATGGAGAAGCGTTTACAG<br>TAAGGTTGCCAGTTACATCGAAGATGAACATCTGCGCCAGGCGTTTTCTTTCCACTCGCTGTTGGTGGGCGG<br>CAATCCCTTCGCCACCTCATCCATTTATACGTTGATACACGCGCTGGAGCGTGAGTGGGGCGTCTGGTTTCCG<br>CGTGGCGGCACCGGCGCATTAGTTCAGGGGATGATAAAGCTGTTTCAGGATCTGGGTGGCGAAGTCGTGTT<br>AAACGCCAGAGTCAGCCATATGGAAACGACAGGAAACAAGATTGAAGCCGTGCATTTAGAGGACGGTCGC<br>AGGTTCCTGACGCAAGCCGTCGCGTCAAATGCAGATGTGGTTCATACCTATCGCGACCTGTTAAGCCAGCAC<br>CCTGCCGCGGTTAAGCAGTCCAACAAACTGCAGACTAAGCGCATGAGTAACTCTCTGTTTGTGCTCTATTTTG<br>GTTTGAATCACCATCATGATCAGCTCGCGCATCACACGGTTTGTTTCGGCCCGCGTTACCGCGAGCTGATTGA<br>CGAAATTTTTAATCATGATGGCCTCGCAGAGGACTTCTCACTTTATCTGCACGCGCCCTGTGTCACGGATTCG<br>TCACTGGCGCCTGAAGGTTGCGGCAGTTACTATGTGTTGGCGCCGGTGCCGCATTTAGGCACCGCGAACCTC<br>GACTGGACGGTTGAGGGGCCAAAACTACGCGACCGTATTTTTGCGTACCTTGAGCAGCATTACATGCCTGGC<br>TTACGGAGTCAGCTGGTCACGCACCGGATGTTTACGCCGTTTGATTTTCGCGACCAGCTTAATGCCTATCATG<br>GCTCAGCCTTTTCTGTGGAGCCCGTTCTTACCCAGAGCGCCTGGTTTCGGCCGCATAACCGCGATAAAACCAT<br>TACTAATCTCTACCTGGTCGGCGCAGGCACGCATCCCGGCGCAGGCATTCCTGGCGTCATCGGCTCGGCAAA<br>AGCGACAGCAGGTTTGATGCTGGAGGATCTGATATGA	1
CrtY	Gene	ATGCAACCGCATTATGATCTGATTCTCGTGGGGGCTGGACTCGCGAATGGCCTTATCGCCCTGCGACTTCAG<br>CAGCAGCAACCTGATATGCGTATTTTGCTTATCGACGCCGCACCCCAGGCGGGCGGGAATCATACGTGGTCA<br>TTTCACCACGATGATTTGACTGAGAGCCAACATCGTTGGATAGCTCCGCTGGTGGTTCATCACTGGCCCGACT<br>ATCAGGTACGCTTTCCCACACGCCGTCGTAAGCTGAACAGCGGCTACTTTTGTATTACTTCTCAGCGTTTCGC<br>TGAGGTTTTACAGCGACAGTTTGGCCCGCACTTGTGGATGGATACCGCGGTCGCAGAGGTTAATGCGGAAT<br>CTGTTCGGTTGAAAAAGGGTCAGGTTATCGGTGCCCGCGCGGTGATTGACGGGCGGGGTTATGCGGCAAAT<br>TCAGCACTGAGCGTGGGCTTCCAGGCGTTTATTGGCCAGGAATGGCGATTGAGCCACCCGCATGGTTTATCA<br>TCTCCCATTATCATGGATGCCACGGTCGATCAGCAAAATGGTTATCGCTTCGTGTACAGCCTGCCGCTCTCGC<br>CGACCAGATTGTTAATTGAAGATACGCACTATATTGATAATGCGACATTAGATCCTGAATGCGCGCGGCAAA<br>ATATTTGCGACTATGCCGCGCAACAGGGTTGGCAGCTTCAGACACTGCTGCGAGAAGAACAGGGCGCCTTA<br>CCCATTACTCTGTCGGGCAATGCCGACGCATTCTGGCAGCAGCGCCCCCTGGCCTGTAGTGGATTACGTGCC<br>GGTCTGTTCCATCCTACCACCGGCTATTCACTGCCGCTGGCGGTTGCCGTGGCCGACCGCCTGAGTGCACTT<br>GATGTCTTTACGTCGGCCTCAATTCACCATGCCATTACGCATTTTGCCCGCGAGCGCTGGCAGCAGCAGGGC<br>TTTTTCCGCATGCTGAATCGCATGCTGTTTTTAGCCGGACCCGCCGATTCACGCTGGCGGGTTATGCAGCGTT<br>TTTATGGTTTACCTGAAGATTTAATTGCCCGTTTTTATGCGGGAAAACTCACGCTGACCGATCGGCTACGTAT<br>TCTGAGCGGCAAGCCGCCTGTTCCGGTATTAGCAGCATTGCAAGCCATTATGACGACTCATCGTTAA	1
dBroccoli	RNA Aptamer	TTGCCATGTGTATGTGGGAGACGGTCGGGTCCATCTGAGACGGTCGGGTCCAGATATTCGTATCTGTCGAGT<br>AGAGTGTGGGCTCAGATGTCGAGTAGAGTGTGGGCTCCCACATACTCTGATGATCCAGACGGTCGGGTCCA<br>TCTGAGACGGTCGGGTCCAGATATTCGTATCTGTCGAGTAGAGTGTGGGCTCAGATGTCGAGTAGAGTGTG<br>GGCTGGATCATTCATGGCAA	15
STAR	Regulatory RNA	TGAACTGTATACATTCCCCGCTGAACGACGGAAACTTTGACTGGACTGACTTGATGACTGG	8
STAR toehold 0	Regulatory RNA	TGAACTGTATACATTCCCCGCTGAACGACGGAAACTTTGACTGGACTGACTTGATGACTGGTAACTCCATTCC<br>ATC	This study
STAR toehold 1	Regulatory RNA	TGAACTGTATACATTCCCCGCTGAACGACGGAAACTTTGACTGGACTGACTTGATGACTGGTCTTATCTTATC<br>TA	This study
STAR toehold 2	Regulatory RNA	TGAACTGTATACATTCCCCGCTGAACGACGGAAACTTTGACTGGACTGACTTGATGACTGGAGTTTGATTAC<br>ATT	This study
STAR toehold 3	Regulatory RNA	TGAACTGTATACATTCCCCGCTGAACGACGGAAACTTTGACTGGACTGACTTGATGACTGGATCTATTACTAC<br>TT	This study
STAR toehold 4	Regulatory RNA	TGAACTGTATACATTCCCCGCTGAACGACGGAAACTTTGACTGGACTGACTTGATGACTGGCGATTATGGAT<br>TAG	This study
STAR toehold 5	Regulatory RNA	TGAACTGTATACATTCCCCGCTGAACGACGGAAACTTTGACTGGACTGACTTGATGACTGGTATGTAATTGA<br>TTT	This study
Anti-STAR	Regulatory RNA	CCAGTCATCAAGTCAGTCCAGTCAAAGTTTCCGTCGTTCAGCGGGGAATGTATACAGTTCA	This study
Anti-STAR toehold 0	Regulatory RNA	GATGGAATGGAGTTACCAGTCATCAAGTCAGTCCAGTCAAAGTTTCCGTCGTTCAGCGGGGAATGTATACAG<br>TTCA	This study
Anti-STAR toehold 1	Regulatory RNA	TAGATAAGATAAGACCAGTCATCAAGTCAGTCCAGTCAAAGTTTCCGTCGTTCAGCGGGGAATGTATACAGT<br>TCA	This study
Anti-STAR toehold 2	Regulatory RNA	AATGTAATCAAACTCCAGTCATCAAGTCAGTCCAGTCAAAGTTTCCGTCGTTCAGCGGGGAATGTATACAGTT<br>CA	This study
Anti-STAR toehold 3	Regulatory RNA	AAGTAGTAATAGATCCAGTCATCAAGTCAGTCCAGTCAAAGTTTCCGTCGTTCAGCGGGGAATGTATACAGT<br>TCA	This study

Anti-STAR toehold 4	Regulatory RNA	CTAATCCATAATCGCCAGTCATCAAGTCAGTCCAGTCAAAGTTTCCGTCGTTCAGCGGGGAATGTATACAGTTCA	This study
Anti-STAR toehold 5	Regulatory RNA	AAATCAATTACATACCAGTCATCAAGTCAGTCCAGTCAAAGTTTCCGTCGTTCAGCGGGGAATGTATACAGTTCA	This study
STAR Target	Regulatory RNA	CCAGTCATCAAGTCAGTCCAGTCAAAGTTTCCGTCGTTCAGCGGGGAATGTATACAGTTCATGTATATATTCCCCGCTTTTTTTTT	8
Deoptimised STAR Target	Regulatory RNA	CCAGTCATCAAGTCAGTCCAGTCAAAGTTTCCCTGGTTCAGCGGGGAATGTATACAGTTCATGTATATATTCCCCGCTTTTTTTTT	This study
Buffer 3	Non-coding region	GGATCCTTACTCGAGAAAAAAAAACCCCGCTTCGGCGGGGTTTTTTTTTCTGGACTGCAGGCTTCCTCGCTCAC	This study
