# Supplementary material for: Burden-aware feedback control of microbial consortia
Source: Nat Commun. 2026 May 6;17:6100. doi: 10.1038/s41467-026-72389-6 (PMC13357841; doi:10.1038/s41467-026-72389-6)
Supplement: Supplementary file 4 — Supplementary Data 2 [file 41467_2026_72389_MOESM4_ESM.pdf]

Supplementary Data 2: Plasmids used in this study

Figure	Plasmid	Description	Source
Figure 2B	pJBL5939	J23119 – STAR Target – RBS_{RFP} – mRFP1 – TrrnB – p15A – CamR	[1]
	pAB300	« LLL_1 » : P_{tet} – B0034 – luxR – B0015 – AraC – P_{araBAD} – STAR_toehold2 – t500 – Buffer 3 – P_{lux} – anti-STAR_toehold2 – t500 – ColE1 – SpecR	This study
	pAB317	« LLL_2 » : P_{tet} – B0034 – luxR – B0015 – AraC – P_{araBAD} – STAR – t500 – Buffer 3 – P_{lux} – anti-STAR – t500 – ColE1 – SpecR	This study
	pAB545	« LLL_3 » : P_{tet} – B0030 – luxR – B0015 – AraC – P_{araBAD} – STAR_toehold2 – t500 – Buffer 3 – P_{lux} – anti-STAR_toehold2 – t500 – ColE1 – SpecR	This study
	pAB401	« LRR » : P_{tet} – B0034 – rpaR – B0015 – AraC – P_{araBAD} – STAR_toehold2 – t500 – Buffer 3 – P_{lux} – anti-STAR_toehold2 – t500 – ColE1 – SpecR	This study
Figure 2C	pAB300	« LLL_1 » : P_{tet} – B0034 – luxR – B0015 – AraC – P_{araBAD} – STAR_toehold2 – t500 – Buffer 3 – P_{lux} – anti-STAR_toehold2 – t500 – ColE1 – SpecR	This study
	pAB368	J23119 – STAR Target – RBS_{Bujard} – eforRed – TrrnB – p15A – CmR	This study
Figure 2D	pAB300	« LLL_1 » : P_{tet} – B0034 – luxR – B0015 – AraC – P_{araBAD} – STAR_toehold2 – t500 – Buffer 3 – P_{lux} – anti-STAR_toehold2 – t500 – ColE1 – SpecR	This study
	pAB517	J23100 – STAR Target – RBS_{Bujard} – VioB-mCherry – L3S2P55 – p15A – CamR	This study
Figure 2E	pAB401	P_{tet} – B0034 – rpaR – B0015 – AraC – P_{araBAD} – STAR_toehold2 – t500 – Buffer 3 – P_{lux} – anti-STAR_toehold2 – t500 – ColE1 – SpecR	This study
	pAB550	J23114 – STAR Target – RBS_{CrtE} – CrtE – RBS_{CrtB} – CrtB – RBS_{CrtI} – CrtI – RBS_{CrtY} – CrtY – L3S2P55 – p15A – CamR	This study
Figure 2F	pAB300	« LLL_1 » : P_{tet} – B0034 – luxR – B0015 – AraC – P_{araBAD} – STAR_toehold2 – t500 – Buffer 3 – P_{lux} – anti-STAR_toehold2 – t500 – ColE1 – SpecR	This study
	pAB205	P_{RAND14} – RBS8B – dCas9 – rrnB T1 – J23119 – STAR Target 6 – HH – gRNA(LeuLp, End-0: A→C) – HDV – p15A – AmpR	This study
Figure 3B	pAB317	« LLL_2 » : P_{tet} – B0034 – luxR – B0015 – AraC – P_{araBAD} – STAR – t500 – Buffer 3 – P_{lux} – anti-STAR – t500 – ColE1 – SpecR	This study
	pAB537	P_{tet} – B0034 – rpaR – B0015 – AraC – P_{rhaBAD} – STAR_toehold2 – t500 – Buffer 3 – P_{lux} – anti-STAR_toehold2 – t500 – ColE1 – SpecR	This study
	pAB518	J23100 – STAR Target 6 – RBS_{Bujard} – VioB-sfYFP – L3S2P55 – p15A – CamR	This study
	pAB519	J23100 – STAR Target 6 – RBS_{Bujard} – VioB-sfCFP – L3S2P55 – p15A – CamR	This study
Figure 4B1-2, Figure 4C	pAB300	« LLL_1 » : P_{tet} – B0034 – luxR – B0015 – AraC – P_{araBAD} – STAR_toehold2 – t500 – Buffer 3 – P_{lux} – anti-STAR_toehold2 – t500 – ColE1 – SpecR	This study
	pAB537	P_{tet} – B0034 – rpaR – B0015 – AraC – P_{rhaBAD} – STAR_toehold2 – t500 – Buffer 3 – P_{lux} – anti-STAR_toehold2 – t500 – ColE1 – SpecR	This study
	pAB518	J23100 – STAR Target 6 – RBS_{Bujard} – VioB-sfYFP – L3S2P55 – p15A – CamR	This study
	pAB519	J23100 – STAR Target 6 – RBS_{Bujard} – VioB-sfCFP – L3S2P55 – p15A – CamR	This study
Figure 4B3, Figure 4C	pAB317	« LLL_2 » : P_{tet} – B0034 – luxR – B0015 – AraC – P_{araBAD} – STAR – t500 – Buffer 3 – P_{lux} – anti-STAR – t500 – ColE1 – SpecR	This study
	pAB537	P_{tet} – B0034 – rpaR – B0015 – AraC – P_{rhaBAD} – STAR_toehold2 – t500 – Buffer 3 – P_{lux} – anti-STAR_toehold2 – t500 – ColE1 – SpecR	This study
	pAB518	J23100 – STAR Target 6 – RBS_{Bujard} – VioB-sfYFP – L3S2P55 – p15A – CamR	This study
	pAB519	J23100 – STAR Target 6 – RBS_{Bujard} – VioB-sfCFP – L3S2P55 – p15A – CamR	This study
Figure 4B4, Figure 4C, Figure 4D, Figure 4E, Figure 4F, Figure 4G,	pAB317	« LLL_2 » : P_{tet} – B0034 – luxR – B0015 – AraC – P_{araBAD} – STAR – t500 – Buffer 3 – P_{lux} – anti-STAR – t500 – ColE1 – SpecR	This study
	pAB401	« LRR » : P_{tet} – B0034 – rpaR – B0015 – AraC – P_{araBAD} – STAR_toehold2 – t500 – Buffer 3 – P_{lux} – anti-STAR_toehold2 – t500 – ColE1 – SpecR	This study
	pAB518	J23100 – STAR Target 6 – RBS_{Bujard} – VioB-sfYFP – L3S2P55 – p15A – CamR	This study
	pAB519	J23100 – STAR Target 6 – RBS_{Bujard} – VioB-sfCFP – L3S2P55 – p15A – CamR	This study
Supplementary Figure 2B-D	pJBL5939	J23119 – STAR Target – RBS_{RFP} – mRFP1 – TrrnB – p15A – CamR	[1]
	pAB161	P_{tet} – B0034 – luxR – B0015 – J23100 – STAR toehold0 – t500 – P_{lux} – anti-STAR toehold0 – t500 – ColE1 – SpecR	This study
	pAB232	P_{tet} – B0034 – luxR – B0015 – J23100 – STAR toehold1 – t500 – P_{lux} – anti-STAR toehold1 – t500 – ColE1 – SpecR	This study

	pAB233	P_{tet} – B0034 – luxR – B0015 – J23100 – STAR toehold2 – t500 – P_{lux} – anti-STAR toehold2 – t500 – ColE1 – SpecR	This study
	pAB234	P_{tet} – B0034 – luxR – B0015 – J23100 – STAR toehold3 – t500 – P_{lux} – anti-STAR toehold3– t500 – ColE1 – SpecR	This study
	pAB235	P_{tet} – B0034 – luxR – B0015 – J23100 – STAR toehold4– t500 – P_{lux} – anti-STAR toehold4 – t500 – ColE1 – SpecR	This study
	pAB236	P_{tet} – B0034 – luxR – B0015 – J23100 – STAR toehold5 – t500 – P_{lux} – anti-STAR toehold5 – t500 – ColE1 – SpecR	This study
Supplementary Figure 3B-D	pJBL5939	J23119 – STAR Target – RBS_{RFP} – mRFP1 – TrrnB – p15A – CamR	[1]
	pAB271	AraC – P_{araBAD} – STAR toehold2 – t500 – ColE1 – SpecR	This study
	pAB298	P_{tet} – B0034 – luxR – B0015 – P_{lux} – anti-STAR toehold2 – t500 – ColE1 – SpecR	This study
	pAB300	« LLL_1 » : P_{tet} – B0034 – luxR – B0015 – AraC – P_{araBAD} – STAR_toehold2 – t500 – Buffer 3 – P_{lux} – anti-STAR_toehold2 – t500 – ColE1 – SpecR	This study
	pAB317	« LLL_2 » : P_{tet} – B0034 – luxR – B0015 – AraC – P_{araBAD} – STAR – t500 – Buffer 3 – P_{lux} – anti-STAR – t500 – ColE1 – SpecR	This study
	pAB303	AraC – P_{araBAD} – STAR – t500 – ColE1 – SpecR	This study
	pAB304	P_{tet} – B0034 – luxR – B0015 – P_{lux} – anti-STAR – t500 – ColE1 – SpecR	This study
Supplementary Figure 4B	pJBL5939	J23119 – STAR Target – RBS_{RFP} – mRFP1 – TrrnB – p15A – CamR	[1]
	pAB262	J23119 – STAR Target – STAR Target – RBS_{RFP} – mRFP1 – TrrnB – p15A – CamR	This study
	pAB127	P_{tet} – B0034 – luxR – B0015 – P_{lux} – STAR toehold0 – t500 – ColE1 – SpecR	This study
Supplementary Figure 4D	pJBL5939	J23119 – STAR Target – RBS_{RFP} – mRFP1 – TrrnB – p15A – CamR	[1]
	pAB262	J23119 – STAR Target – STAR Target – RBS_{RFP} – mRFP1 – TrrnB – p15A – CamR	This study
	pAB161	P_{tet} – B0034 – luxR – B0015 – J23100 – STAR toehold0 – t500 – P_{lux} – anti-STAR toehold0 – t500 – ColE1 – SpecR	This study
Supplementary Figure 6B	pJBL5939	J23119 – STAR Target – RBS_{RFP} – mRFP1 – TrrnB – p15A – CamR	[1]
	pAB300	« LLL_1 » : P_{tet} – B0034 – luxR – B0015 – AraC – P_{araBAD} – STAR_toehold2 – t500 – Buffer 3 – P_{lux} – anti-STAR_toehold2 – t500 – ColE1 – SpecR	This study
	pAB401	« LRR » : P_{tet} – B0034 – rpaR – B0015 – AraC – P_{araBAD} – STAR_toehold2 – t500 – Buffer 3 – P_{lux} – anti-STAR_toehold2 – t500 – ColE1 – SpecR	This study
Supplementary Figure 7B	pAB300	« LLL_1 » : P_{tet} – B0034 – luxR – B0015 – AraC – P_{araBAD} – STAR_toehold2 – t500 – Buffer 3 – P_{lux} – anti-STAR_toehold2 – t500 – ColE1 – SpecR	This study
	pAB367	J23119 – STAR Target – RBS_{Bujard} – gfasPurple – TrrnB – p15A – CmR	This study
	pAB368	J23119 – STAR Target – RBS_{Bujard} – eforRed – TrrnB – p15A – CmR	This study
	pAB369	J23119 – STAR Target – RBS_{Bujard} – cjBlue – TrrnB – p15A – CmR	This study
	pAB370	J23119 – STAR Target – RBS_{Bujard} – fwYellow – TrrnB – p15A – CmR	This study
Supplementary Figure 8B-C	pAB300	« LLL_1 » : P_{tet} – B0034 – luxR – B0015 – AraC – P_{araBAD} – STAR_toehold2 – t500 – Buffer 3 – P_{lux} – anti-STAR_toehold2 – t500 – ColE1 – SpecR	This study
	pAB317	« LLL_2 » : P_{tet} – B0034 – luxR – B0015 – AraC – P_{araBAD} – STAR – t500 – Buffer 3 – P_{lux} – anti-STAR – t500 – ColE1 – SpecR	This study
Supplementary Figure 9B-D	pAB300	« LLL_1 » : P_{tet} – B0034 – luxR – B0015 – AraC – P_{araBAD} – STAR_toehold2 – t500 – Buffer 3 – P_{lux} – anti-STAR_toehold2 – t500 – ColE1 – SpecR	This study
	pAB517	J23100 – STAR Target – RBS_{Bujard} – VioB-mCherry – L3S2P55 – p15A – CamR	This study
Supplementary Figure 10B-E	pAB401	« LRR » : P_{tet} – B0034 – rpaR – B0015 – AraC – P_{araBAD} – STAR_toehold2 – t500 – Buffer 3 – P_{lux} – anti-STAR_toehold2 – t500 – ColE1 – SpecR	This study
	pAB550	J23114 – STAR Target – RBS_{CrtE} – CrtE – RBS_{CrtB} – CrtB – RBS_{CrtI} – CrtI – RBS_{CrtY} – CrtY – L3S2P55 – p15A – CamR	This study
Supplementary Figure 11C	B0034_mKate	J23106 – B0034 – mKate – B0015 – pMB1 – CamR	[2]
	pAB81	P_{RAND14} – RBS8B – dCas9 – rrnB T1 – P_{araBAD} – HH – gRNA(LeuLp) – HDV – p15A – AmpR	This study
Supplementary Figure 12B,D	pAB58	P_{RAND14} – RBS8B – dCas9 – rrnB T1 – AraC - P_{araBAD} – No Target gRNA – p15A – AmpR	This study

	pAB60	P_{RAND14} – RBS8B – dCas9 – rrnB T1 – AraC - P_{araBAD} – gRNA(HisLp) – p15A – AmpR	This study
	pAB61	P_{RAND14} – RBS8B – dCas9 – rrnB T1 – AraC – P_{araBAD} – gRNA(LeuLp) – p15A – AmpR	This study
Supplementary Figure 13B,D	pAB58	P_{RAND14} – RBS8B – dCas9 – rrnB T1 – AraC - P_{araBAD} – No Target gRNA – p15A – AmpR	This study
	pAB96	P_{RAND14} – RBS8B – dCas9 – rrnB T1 – J23119 – STAR Target 6 – HH – gRNA(LeuLp) – HDV – p15A – AmpR	This study
	pAB205	P_{RAND14} – RBS8B – dCas9 – rrnB T1 – J23119 – STAR Target 6 – HH – gRNA(LeuLp, End-0: A→C) – HDV – p15A – AmpR	This study
Supplementary Figure 14B	pAB96	P_{RAND14} – RBS8B – dCas9 – rrnB T1 – J23119 – STAR Target 6 – HH – gRNA(LeuLp) – HDV – p15A – AmpR	This study
	pAB205	P_{RAND14} – RBS8B – dCas9 – rrnB T1 – J23119 – STAR Target 6 – HH – gRNA(LeuLp, End-0 mutation) – HDV – p15A – AmpR	This study
	pAB206	P_{RAND14} – RBS8B – dCas9 – rrnB T1 – J23119 – STAR Target 6 – HH – gRNA(LeuLp, End-1 mutation) – HDV – p15A – AmpR	This study
	pAB208	P_{RAND14} – RBS8B – dCas9 – rrnB T1 – J23119 – STAR Target 6 – HH – gRNA(LeuLp, End-3 mutation) – HDV – p15A – AmpR	This study
	pAB127	P_{tet} – B0034 – luxR – B0015 – AraC – P_{araBAD} – STAR_toehold0 – t500 – ColE1 – SpecR	This study
Supplementary Figure 17B-D	pAB420	P_{tet} – B0034 – luxR – L3S2P55 – P_{lux} – dBroccoli – L3S2P21 – ColE1 – SpecR	This study
Supplementary Figure 18B	pAB420	« Lux Receiver » : P_{tet} – B0034 – luxR – L3S2P55 – P_{lux} – dBroccoli – L3S2P21 – ColE1 – SpecR	This study
Supplementary Figure 18D	pAB421	« Rpa Receiver » : P_{tet} – B0034 – rpaR – L3S2P55 – P_{lux} – dBroccoli – L3S2P21 – ColE1 – SpecR	This study
Supplementary Figure 19B,D	pAB420	« Lux Receiver » : P_{tet} – B0034 – luxR – L3S2P55 – P_{lux} – dBroccoli – L3S2P21 – ColE1 – SpecR	This study
	pAB421	« Rpa Receiver » : P_{tet} – B0034 – rpaR – L3S2P55 – P_{lux} – dBroccoli – L3S2P21 – ColE1 – SpecR	This study
Supplementary Figure 20B	pAB420	« Lux Receiver » : P_{tet} – B0034 – luxR – L3S2P55 – P_{lux} – dBroccoli – L3S2P21 – ColE1 – SpecR	This study
	pAB421	« Rpa Receiver » : P_{tet} – B0034 – rpaR – L3S2P55 – P_{lux} – dBroccoli – L3S2P21 – ColE1 – SpecR	This study
Supplementary Figure 21B,D	pJBL5939	J23119 – STAR Target – RBS_{RFP} – mRFP1 – TrrnB – p15A – CamR	[1]
	pAB300	« LLL_1 » : P_{tet} – B0034 – luxR – B0015 – AraC – P_{araBAD} – STAR_toehold2 – t500 – Buffer 3 – P_{lux} – anti-STAR_toehold2 – t500 – ColE1 – SpecR	This study
	pAB401	« LRR » : P_{tet} – B0034 – rpaR – B0015 – AraC – P_{araBAD} – STAR_toehold2 – t500 – Buffer 3 – P_{lux} – anti-STAR_toehold2 – t500 – ColE1 – SpecR	This study
Supplementary Figure 22A-C	pAB252	P_{tet} – B0034 – luxR – L3S2P55 – P_{lux} – RBSc33 – sfGFP – L3S2P21 – ColE1 – SpecR	This study
	pAB409	AraC – P_{araBAD} – RBSc33 – sfGFP – L3S2P21 – ColE1 – SpecR	This study
	pAB410	P_{rhaBAD} – RBSc33 – sfGFP – L3S2P21 – ColE1 – SpecR	This study
Supplementary Figure 23B-C	pAB300	« LLL_1 » : P_{tet} – B0034 – luxR – B0015 – AraC – P_{araBAD} – STAR_toehold2 – t500 – Buffer 3 – P_{lux} – anti-STAR_toehold2 – t500 – ColE1 – SpecR	This study
	pAB518	J23100 – STAR Target 6 – RBS_{Bujard} – VioB-sfYFP – L3S2P55 – p15A – CamR	This study
Supplementary Figure 23E-F	pAB401	« LRR » : P_{tet} – B0034 – rpaR – B0015 – AraC – P_{araBAD} – STAR_toehold2 – t500 – Buffer 3 – P_{lux} – anti-STAR_toehold2 – t500 – ColE1 – SpecR	This study
	pAB519	J23100 – STAR Target 6 – RBS_{Bujard} – VioB-sfCFP – L3S2P55 – p15A – CamR	This study
Supplementary Figure 24B-F	pAB300	« LLL_1 » : P_{tet} – B0034 – luxR – B0015 – AraC – P_{araBAD} – STAR_toehold2 – t500 – Buffer 3 – P_{lux} – anti-STAR_toehold2 – t500 – ColE1 – SpecR	This study
	pAB537	P_{tet} – B0034 – rpaR – B0015 – AraC – P_{rhaBAD} – STAR_toehold2 – t500 – Buffer 3 – P_{lux} – anti-STAR_toehold2 – t500 – ColE1 – SpecR	This study
	pAB518	J23100 – STAR Target 6 – RBS_{Bujard} – VioB-sfYFP – L3S2P55 – p15A – CamR	This study
	pAB519	J23100 – STAR Target 6 – RBS_{Bujard} – VioB-sfCFP – L3S2P55 – p15A – CamR	This study
Supplementary Figure 25B-D	pAB399	P_{tet} – B0034 – luxR – B0015 – P_{rhaBAD} – STAR_toehold2 – t500 – Buffer 3 – P_{lux} – anti-STAR_toehold2 – t500 – ColE1 – SpecR	This study

	pAB401	« LRR » : P_{tet} – B0034 – rpaR – B0015 – AraC – P_{araBAD} – STAR_toehold2 – t500 – Buffer 3 – P_{lux} – anti-STAR_toehold2 – t500 – ColE1 – SpecR	This study
	pAB518	J23100 – STAR Target 6 – RBS_{Bujard} – VioB-sfYFP – L3S2P55 – p15A – CamR	This study
	pAB519	J23100 – STAR Target 6 – RBS_{Bujard} – VioB-sfCFP – L3S2P55 – p15A – CamR	This study
Supplementary Figure 26	pAB317	« LLL_2 » : P_{tet} – B0034 – luxR – B0015 – AraC – P_{araBAD} – STAR – t500 – Buffer 3 – P_{lux} – anti-STAR – t500 – ColE1 – SpecR	This study
	pAB401	« LRR » : P_{tet} – B0034 – rpaR – B0015 – AraC – P_{araBAD} – STAR_toehold2 – t500 – Buffer 3 – P_{lux} – anti-STAR_toehold2 – t500 – ColE1 – SpecR	This study
	pAB518	J23100 – STAR Target 6 – RBS_{Bujard} – VioB-sfYFP – L3S2P55 – p15A – CamR	This study
	pAB519	J23100 – STAR Target 6 – RBS_{Bujard} – VioB-sfCFP – L3S2P55 – p15A – CamR	This study

All plasmid maps are available on Zenodo: https://doi.org/10.5281/zenodo.7757475

1. Chappell, J., Westbrook, A., Verosloff, M. & Lucks, J. B. Computational design of small transcription activating RNAs for versatile and dynamic gene regulation. *Nat. Commun.* **8**, 1–11 (2017).
2. Borkowski, O. *et al.* Cell-free prediction of protein expression costs for growing cells. *Nat. Commun.* **9**, (2018).
